# Mitochondrial Ceramide-Rich Macrodomains Functionalize Bax upon Irradiation

**DOI:** 10.1371/journal.pone.0019783

**Published:** 2011-06-13

**Authors:** Hyunmi Lee, Jimmy A. Rotolo, Judith Mesicek, Tuula Penate-Medina, Andreas Rimner, Wen-Chieh Liao, Xianglei Yin, Govind Ragupathi, Desiree Ehleiter, Erich Gulbins, Dayong Zhai, John C. Reed, Adriana Haimovitz-Friedman, Zvi Fuks, Richard Kolesnick

**Affiliations:** 1 Laboratory of Signal Transduction, Memorial Sloan-Kettering Cancer Center, New York, New York, United States of America; 2 Department of Radiation Oncology, Memorial Sloan-Kettering Cancer Center, New York, New York, United States of America; 3 Laboratory of Tumor Vaccinology, Memorial Sloan-Kettering Cancer Center, New York, New York, United States of America; 4 Department of Molecular Biology, University of Duisburg-Essen, Essen, Germany; 5 Sanford-Burnham Medical Research Institute, La Jolla, California, United States of America; Cleveland Clinic, United States of America

## Abstract

**Background:**

Evidence indicates that Bax functions as a “lipidic” pore to regulate mitochondrial outer membrane permeabilization (MOMP), the apoptosis commitment step, through unknown membrane elements. Here we show mitochondrial ceramide elevation facilitates MOMP-mediated cytochrome *c* release in HeLa cells by generating a previously-unrecognized mitochondrial ceramide-rich macrodomain (MCRM), which we visualize and isolate, into which Bax integrates.

**Methodology/Principal Findings:**

MCRMs, virtually non-existent in resting cells, form upon irradiation coupled to ceramide synthase-mediated ceramide elevation, optimizing Bax insertion/oligomerization and MOMP. MCRMs are detected by confocal microscopy in intact HeLa cells and isolated biophysically as a light membrane fraction from HeLa cell lysates. Inhibiting ceramide generation using a well-defined natural ceramide synthase inhibitor, Fumonisin B1, prevented radiation-induced Bax insertion, oligomerization and MOMP. MCRM deconstruction using purified mouse hepatic mitochondria revealed ceramide alone is non-apoptogenic. Rather Bax integrates into MCRMs, oligomerizing therein, conferring 1–2 log enhanced cytochrome *c* release. Consistent with this mechanism, MCRM Bax isolates as high molecular weight “pore-forming” oligomers, while non-MCRM membrane contains exclusively MOMP-incompatible monomeric Bax.

**Conclusions/Significance:**

Our recent studies in the *C. elegans* germline indicate that mitochondrial ceramide generation is obligate for radiation-induced apoptosis, although a mechanism for ceramide action was not delineated. Here we demonstrate that ceramide, generated in the mitochondrial outer membrane of mammalian cells upon irradiation, forms a platform into which Bax inserts, oligomerizes and functionalizes as a pore. We posit conceptualization of ceramide as a membrane-based stress calibrator, driving membrane macrodomain organization, which in mitochondria regulates intensity of Bax-induced MOMP, and is pharmacologically tractable *in vitro* and *in vivo*.

## Introduction

We recently reported that the ceramide is an essential element in the mitochondrial phase of apoptosis in the *C. elegans* germline [1]. Ionizing radiation activated the *de novo* ceramide synthetic pathway via the *C. elegans* ceramide synthases (CSs) HYL-1 and LAGR-1, increasing ceramide concentration in germ cell mitochondrial membranes. Mitochondrial ceramide regulated EGL-1 (BH3 ortholog)-mediated displacement of CED-4 (APAF-1 ortholog) from the CED-9 (Bcl-2 ortholog)/CED-4 complex, thus activating CED-3 caspase, conferring the apoptotic effector phase [1]. While genetic CS depletion proved ceramide obligate for mitochondrial CED-4 release, a mechanism for ceramide function remains unknown.

In mammalian cells, a complementary pathway involving mitochondrial outer membrane permeabilization (MOMP) initiates the commitment phase of the apoptotic response. MOMP is regulated either by opening of the inner mitochondrial membrane permeability transition pore or by insertion of pro-apoptotic Bcl-2 family members into the MOM. The principal mammalian pro-apoptotic Bcl-2 protein is α-helical Bax, which undergoes a conscripted sequence of events *en route* to MOMP [2]. Bax contains three Bcl-2 homology domains (BH1-3) and a C-terminal transmembrane (TM) domain, arranged in 9 α-helices [3], [4]. This spatial configuration is reminiscent of the structure of α-helical pore-forming toxins, including diphtheria toxin, colicins and δ-endotoxin [4]. Inactive Bax resides as a 21 KD monomer in cytosol where the amphiphatic helices α1–4 and α7–8 provide a hydration shell for the α5–α6-helical hydrophobic hairpin core in an arrangement that generates an elongated hydrophobic cleft occupied by the hydrophobic α9-helix, thereby constraining the TM domain. Upon stimulation by pro-apoptotic signals, Bax undergoes conformational changes that free up the TM domain, which eventually inserts through the MOM to tether Bax to mitochondria. X-ray and NMR structure evidence, and model membrane studies, suggest the α5–α6-helical anti-parallel duplex of mitochondrial-bound Bax thereafter inserts through the MOM to trigger MOMP [2], [3], [5].

Emerging evidence suggests that Bax insertion into the MOM by itself may be insufficient for pore activation [6], [7], but that homodimerization through BH domain interaction [3], [8] and higher order oligomerization with other membrane resident proteins are required. Furthermore, studies with pore-forming toxins, and with His-Bax [7], indicate the pore once assembled requires formation of a toroidal structure, where pore walls interact directly with membrane lipids, such that non-bilayer structures are generated, enabling pore opening [7], [9]. Mitochondrial membrane components that might undergo rearrangement to generate a functional Bax “lipidic” pore remain largely unknown. Hence, at least two lipid events requiring distinct Bax domains are involved in insertional activation of Bax at the MOM.

A lipid candidate potentially involved in Bax-mediated MOMP is the sphingolipid second messenger ceramide. A substantive literature identifies mitochondrial ceramide elevation preceding MOMP for a variety of distinct stresses [10], [11], [12], [13], [14]. Furthermore, in isolated mitochondria ceramide and recombinant Bax act coordinately to release cytochrome C [15], [16], [17]. Consistent with this latter observation ceramide is capable of inducing an activating conformational change in Bax in isolated mitochondrial membranes [17]. Lastly, synergism was detected upon addition of exogenous ceramide and recombinant Bax to isolated yeast mitochondria which are devoid of Bcl-2 family proteins, suggesting a direct lipid-protein interaction [15].

Furthermore, recent studies of α-helical and β-strand toxin activation indicate an initial step often involves targeting plasma membrane cholesterol- and glycosphingolipid-enriched microdomains (GEMs, also known as rafts), and macrodomain platforms derived thereof [18]. GEMs exist constitutively in the outer plasma membrane, phase-separated from the bulk plasma membrane because the sphingosine backbone of sphingolipids forms a network of hydrogen bonds that stabilize a liquid ordered phase within the liquid disordered bilayer [19]. GEMs segregate specific proteins and lipids that operate as “receptors” for antigens, growth factors and cytokines, promoting protein oligomerization and signal transmission [20]. Consistent with this model, α-helical and β-strand pore-forming toxins recognize “receptors” within GEMs. Aerolysin and Cry proteins target GPI-linked GEM proteins, lysenin targets sphingomyelin, and some β-strand pore-forming toxins are cholesterol-binding cytolysins [21], [22], [23]. Pharmacologic GEM disruption dramatically affects toxin pore-forming capability [23], [24], [25], [26]. A large literature now reports that specific stresses (i.e. oxidative, heat, ionizing radiation, UV-C, microbial pathogens and TNF-superfamily members) may induce structural re-organization of plasma membrane GEMs to form large (1–5 microns in diameter) sphingolipid- and cholesterol-rich platforms into which select proteins traffic, oligomerize and signal [for review see [27]]. This process is initiated by acid sphingomyelinase-mediated sphingomyelin hydrolysis to ceramide, which drives GEM coalescence based largely on hydrophobic forces [19], [28], [29]. In contrast to GEMs, which are too small to visualize by conventional microscopy, these ceramide-rich macrodomains (CRMs) can be detected by confocal microscopy [30], [31]. In at least one instance such CRMs regulate channel properties. *Pseudomonas aeruginosa* binds asialoGM1 within GEMs, stimulating formation of ceramide-rich platforms into which the cystic fibrosis transmembrane conductance regulator (CFTR) chloride channel inserts, oligomerizes and becomes functional. This response was abrogated in acid sphingomyelinase-deficient cells [28].

Here we visualize and isolate a previously-unrecognized mitochondrial ceramide-rich macrodomain (MCRM) into which Bax integrates. MCRM formation is coupled to radiation-induced activation of CS and ceramide elevation within HeLa cell MOM, optimizing Bax insertion/oligomerization therein, triggering MOMP. Whereas pharmacologic inhibition of CS abrogates MCRM formation, Bax insertion/oligomerization and MOMP, MCRMs appear to bestow Bax pore-forming capability. We posit conceptualization of ceramide as a membrane-based stress calibrator, driving membrane macrodomain organization, which in mitochondria regulates intensity of Bax-induced MOMP.

## Materials and Methods

### Cell culture, irradiation and FB_1_-treatment

HeLa cells (ATCC) were cultured in low glucose Dulbecco's Modified Eagle's Medium (DMEM from Gibco BRL) containing 10% fetal bovine serum (FBS), penicillin (50 units/ml), streptomycin (50 µg/ml) and 2 mM glutamine. 1 h prior to irradiation, HeLa cells were changed to medium containing 0.2% FBS. BAEC were cultured until confluent in low glucose DMEM supplemented with 10% normal calf serum, 1 ng/ml basic FGF (Scios Inc.), penicillin (50 units/ml), streptomycin (50 µg/ml) and 2 mM glutamine. After confluence, BAEC were maintained in DMEM with 5% heat inactivated normal calf serum. At 24 h before irradiation, medium was changed to DMEM with 0.2% human albumin (HA) containing 50 µg/ml pentosan polysulfate (PPS; Sigma). FB_1_ (Sigma or Biomol) was solubilized in phosphate-buffered saline (PBS; 10 mM KH_2_PO_4_, 150 mM NaCl, 3 mM NaH_2_PO_4_·7H_2_O) at a concentration of 5 mM and added to cells at final concentration of 10–50 µM FB_1_. Note commercially-available FB_1_ is a biologic isolate from *Fusarium verticillioides* and *Fusarium proliferatum* that displays batch-to-batch variation. Hence, each batch must be tested empirically for effectiveness. Irradiation was carried out at 22°C using a Cs-137 irradiator (Shepherd Mark-I, model 68, SN 643) at a dose rate of 240 cGy/min.

### Antibodies and Immunoblotting

Anti-Bax (N20, Santa Cruz; 0.4 µg/ml or YTH-6A7, Trevigen; 0.2 µg/ml), anti-Bak (NT, Upstate Biotechnology; 2 µg/ml), anti-Bcl-xL (Transduction Laboratories; 0.5 µg/ml), anti-BID (AF860 R&D; 1 µg/ml), anti-BIM (Calbiochem; 1∶1000), anti-PUMA (N-terminal, Sigma; 1 µg/ml), anti-GST (Z-5, Santa Cruz; 0.2 µg/ml), anti-Fis1 (Imgenex; 1∶1000), anti-COX II (12C4, Molecular Probes; 0.2 µg/ml), anti-COX IV (20E8, Molecular Probes; 0.2 µg/ml), anti-PDH-E1α (9H9, Molecular Probes; 0.2 µg/ml), anti-Hsp60 (LK-1, Calbiochem; 0.05 µg/ml), anti-KDEL (10C3, Abcam; 1∶50), anti-VDAC [31HL(Ab-3), Calbiochem; 2 µg/ml], anti-Climp63 (G1/296, Alexis; 1 µg/ml), anti-Calnexin (H-70, Santa Cruz; 0.2 µg/ml) or anti-metaxin (Transduction laboratories; 0.5 µg/ml) was used as indicated. Proteins were separated on a 12–15% SDS-PAGE gel and transferred to 0.2 µm PVDF membrane (Biorad). Then the blot was incubated with 5% Blotto (Biorad) in TBST (20 mM Tris–HCl, pH 7.5, 500 mM NaCl, 0.1% Tween 20) for 1 h at room temperature. After brief washing with TBST, the blot was incubated with primary antibodies in TBST containing 2.5% BSA for 16 h at 4°C. This was followed by extensive washing with TBST and incubation with the appropriate secondary antibodies conjugated to HRP (Amersham) in TBST containing 5% Blotto for 1 h at room temperature. After extensive washing with TBST, immunolabeled proteins were revealed using ECL kit (Amersham).

### Apoptosis assay

Apoptotic HeLa cells were stained with *bis-*benzimide Trihydrochloride (Hoechst #33258; Sigma) and quantified by fluorescence microscopy, as described [32].

### Caspase assay

Caspase 3 activity was measured fluorospectrometrically using Z-DEVD-AFC as substrate as described [33].

### Cytochrome *c* release in intact cells

Cytosolic cytochrome *c* content was measured according to Newmeyer [34] by either immunoblotting using mouse monoclonal anti-cytochrome *c* antibody (7H8.2C12, PharMingen; dilution 1∶500) or by ELISA (Human Cytochrome *c* Quantikine ELISA Kit, R&D).

### Isolation of subcellular fractions from HeLa cells and BAEC

Mitochondria and endoplasmic reticulum from HeLa cells and BAEC were isolated by differential centrifugation via a discontinuous sucrose gradient as described [35], with modification. All procedures were conducted at 4°C. For these studies, cell monolayers were washed twice and scraped into ice-cold PBS. Cells were collected by centrifugation at 500×*g* for 10 min, then washed and resuspended in SHE buffer [250 mM sucrose, 10 mM HEPES-KOH pH 7.4, 1 mM EGTA, protease inhibitor cocktail (Roche)], and homogenized twice by 20 strokes in a loose-fitting Dounce homogenizer. Cell debris and nuclei were pelleted by centrifugation at 800×*g* for 5 min. The postnuclear supernatant was collected and centrifuged at 10,000×*g* for 10 min and the pellet containing heavy membrane fractions (P_10_) was resuspended in 20 ml SHE buffer and centrifuged through an underlayed cushion of 5 ml HE medium containing 750 mM sucrose at 10,000×*g* for 30 min. The resulting mitochondrial-enriched pellet was resuspended in SHE buffer and further purified by a second discontinuous sucrose gradient centrifugation as above. MAM-free mitochondria were isolated by Percoll gradient according to the method of Vance [14] with slight modification. For these studies, heavy membrane fractions (P_10_) were layered on top of 20 ml of medium consisting of 2.2 ml of 2.5 M sucrose, 6.55 ml of Percoll and 12.25 ml of 10 mM HEPES, pH 7.4, 1 mM EGTA, and centrifuged at 100,000×*g* for 1 h in a Beckman Ti-55.2 rotor. Two bands were recovered from the gradient; a lower band corresponding to mitochondria (R_f_: 0.48) and an upper band containing MAM (R_f_: 0.23) [36]. Mitochondria were collected using a Pasteur pipette, diluted 10-fold with SHE buffer and washed twice by centrifugation at 10,000×*g* for 10 min to remove the Percoll. The MAM fraction was diluted with SHE buffer and pelleted by centrifugation at 100,000×*g* for 1 h in a Beckman Ti-55.2 rotor. To isolate the ER-enriched membrane fraction, the supernatant of the P_10_ fraction was centrifuged at 100,000×*g* for 1 h. Fractionation was monitored by immunoblotting using mouse monoclonal anti-COX II (mitochondrial marker) and rabbit polyclonal anti-Climp63 or anti-Calnexin (ER Markers).

### Bax insertion status by alkali extraction of the mitochondria

For measurement of Bax insertion, mitochondria, subjected to alkali extraction by incubation in freshly prepared 0.1 M Na_2_CO_3_ (pH 11.5) for 60 min at 4°C, were pelleted by centrifugation at 100,000×*g* for 30 min at 4°C [37], [38]. Supernatant (alkaline-sensitive fraction) and mitochondrial pellet (alkaline-resistant fraction) were separated, mitochondrial membranes were lysed in RIPA buffer, and fractions were analyzed by immunoblotting using anti-Bax antibody.

### Bax oligomerization status by gel filtration

Gel filtrations were performed at 4°C on a 1.5×55 cm Sephacryl 200 column equilibrated in 25 mM HEPES-KOH pH 7.4, 137 mM NaCl, 2 mM EDTA, 10% Glycerol, 1% or 2% (w/v) CHAPS and calibrated with thyroglobulin (669 KD), ferritin (440 KD), aldolase (158 KD), bovine serum albumin (67 KD), ovalbumin (43 KD), chymotrypsinogen A (25 KD), and ribonuclease A (13.7 KD) (Amersham Biosciences). A 1.5 ml sample was loaded onto the column and fractions of 1 ml were collected at a flow rate of 0.5 ml/min. Aliquots of 400 µl from the fractions were analyzed by Western blotting after 20% TCA precipitation. Samples were separated on 12% or 15% SDS-PAGE under reducing conditions and transferred to 0.2 µm PVDF membranes (Biorad). Proteins were detected with the specific antibodies as indicated in the figures, and the blots were developed with the ECL system from Amersham Pharmacia Biotech.

### Ceramide synthase activity assay

For these studies, 75×10^6^ cells were pelleted, washed once with cold PBS, and resuspended in 300 µl of homogenization buffer [25 mM HEPES pH 7.4, 5 mM EGTA, 50 mm NaF, protease inhibitor cocktail (Roche)]. Cells were disrupted on ice by sonication, and lysates centrifuged at 800×*g* for 5 min. The postnuclear supernate was centrifuged at 100,000×*g* for 1 h and the microsomal pellet (P_100_) was resuspended into 1 ml of homogenization buffer. To measure ceramide synthase activity in sub-cellular fractions, mitochondrial-enriched and endoplasmic reticulum-enriched fractions were prepared as described above. Membranes were prepared fresh daily. The assay for ceramide synthase (EC 2.3.1.24) activity was based on the procedure of Harel and Futerman [39]. 75 µg protein were incubated in a 1 ml reaction mixture containing 2 mM MgCl_2_, 20 mM HEPES pH 7.4, 20 µM defatted BSA (Sigma), and varying concentrations (0.2–20 µM) of dihydrosphingosine (sphinganine; Biomol), 70 µM unlabeled palmitoyl-coenzyme A (Sigma), and 3.6 µM (0.2 µCi) [1-^14^C]palmitoyl-coenzyme A (55 mCi/mmol; American Radiolabeled Chemicals). Dihydrosphingosine was dried under N_2_ from a stock solution in 100% ethanol and dissolved by sonication in reaction mixture prior to addition of protein. The reaction was started by addition of palmitoyl-coenzyme A at 37°C, and after 1 h stopped by extraction of lipids using 2 ml of chloroform∶methanol (1∶2, v/v). 500 µl of lower phase was dried under N_2_ and dihydroceramide resolved by thin layer chromatography using a solvent system of chloroform∶methanol∶3.5 N ammonium hydroxide (85∶15∶1). Radiolabeled dihydroceramide was identified by iodine vapor staining based on comigration with ceramide standards (Type III, Sigma), and quantified by liquid scintillation counting. The velocity of the reaction was linear for at least 2 h, and the amount of palmitoyl-coenzyme A consumed did not exceed 5% of total. Ceramide synthase activity was unaffected by CHAPS (0.5–4%) or Triton X-100 (0.05–1%), except at the highest concentrations where both detergents attenuated activity 20–30%.

### Lipid analysis

Ceramide mass was quantified by the diacylglycerol kinase assay as described [32]. Cholesterol was quantified using Amplex Red Cholesterol Assay Kit according to manufacturer's instruction (Invitrogen). Neutral sphingolipids and gangliosides were analyzed by HPTLC or immune-HPTLC as described [40] with modification. Sphingomyelin was analyzed according to Mallikarjuneswara et al. [41]. For these studies, 15.3 µg MCRM was extracted with CHCl_3_∶MeOH∶HCl (100∶100∶1 v/v/v), and the extract was washed once with an equal volume of pre-equilibrated upper phase, followed by resolution on Silica gel 60 Å HPTLC plates (Whatman) using CHCl_3_∶MeOH∶H_2_0 (65∶25∶4) as solvent. Sphingomyelin was visualized with 7% phosphomolybdic acid in ethanol (Sigma) followed by charring, and quantified by comparison to a concomitantly-run standard curve of known quantities of sphingomyelin.

### Cellular ceramide localization by confocal microscopy

HeLa cells, grown on coverslips in 6-well plates, were washed 3 times with cold PBS, and fixed with 4% Formalin in PBS for 15 min. Cells were then permeabilized by incubation with-20°C acetone/methanol (1∶1) for 10 min after 3 times of washing with PBS. After blocking with 3% goat serum and 3% FBS diluted in PBS for 16 h at 4°C, cells were incubated with anti-CoxI-Alexa 488 conjugated (Invitrogen; 1∶75) diluted in cold wash buffer (1% FBS, 0.025% Tween20 in PBS) at 22°C for 45 min. Cells were then washed 3 times for 5 min with cold wash buffer followed by incubation with monoclonal anti–ceramide (Alexis; 1∶30) at 22°C for 45 min. After washing 3 times with cold wash buffer, cells were incubated with Cy3-conjugated goat anti-mouse IgM (Jackson ImmunoResearch; 1∶100) at 22°C. After 45 min, cells were washed 3 times with cold wash buffer, and mounted on slides using the ProLong Antifade Kit (Invitrogen). When indicated, Mitotracker Red CMXRos (50 nM) (Invitrogen) was added to the culture medium at 37°C for 30 min to stain mitochondria before fixation. Images were acquired with a Leica TCS SP2 AOBS confocal microscope equipped with a 63×1.4 NA OIL DIC D Objective combined with 4× scan zoom. Co-localization analysis was performed with MetaMorph 7.5 software. For ER staining, anti-KDEL (Abcam; 1∶50) and anti–ceramide (Alexis; 1∶20) were employed in combination with Texas red-conjugated goat anti-mouse IgG (Jackson ImmunoResearch; 1∶100) and Cy2-conjugated goat anti-mouse IgM (Jackson ImmunoResearch; 1∶100), respectively.

### Preparation of recombinant full length Bax and BaxΔC

Purified recombinant full length Bax was kindly provided by Dr. Richard Youle [see [42]]. Mouse BaxΔC with or without a GST tag was prepared as described [16]. Briefly, 50 ml of an overnight culture of *E. coli BL21* expressing pGEX-4T-2 BaxΔC was inoculated into 1000 ml of LB-medium containing 50 µg/ml of ampicillin and grown at 37°C to an A_600_ 0.6–0.8. After addition of 0.1 mM IPTG, cells were grown for 2 h at 25°C and collected by centrifugation. Cell pellets were resuspended in 30 ml lysis buffer (50 mM Tris pH 8.0, 150 mM NaCl, 1% Tween 20, 0.1% 2-mercaptoethanol, 5 mM EDTA, 1 mM PMSF, protease inhibitor cocktail) and disrupted by sonication. The cell lysate was centrifuged at 28,000×*g* for 20 min at 4°C. GST-BaxΔC was purified using the Bulk GST Purification Kit (Amersham). GST tag was removed from the recombinant GST–BaxΔC proteins bound to glutathione-Sepharose 4B resin by thrombin (10 units, Amersham) according to manufacturer's instructions. Released BaxΔC protein was purified on a Mono-Q Sepharose column using a linear gradient of 0.5 M NaCl, pH 8.0 and dialyzed in dilution buffer (20 mM HEPES pH 7.5, 100 mM KCl, 20 mM MgCl_2_, 1 mM EDTA) for 16 h at 4°C. Ultrafree Centrifuge Filter Devices (Millipore) were used to concentrate purified protein. Recombinant BaxΔC was stored in dilution buffer.

### Preparation of C_16_-ceramide and C_16_-dihydroceramide

C_16_-ceramide (Biomol) or C_16_-dihydroceramide (Toronto Research Chemical Inc.) was dissolved in ethanol∶dodecane (98∶2, v/v) at 37°C with occasional vortexing, and further diluted to a 1% final solvent concentration with experimental reaction buffers.

### Bax insertion into and cytochrome *c* release from HeLa cell mitochondria ex vivo

Bax or tBid (R&D Systems, Inc.) in Bax dilution buffer [20 mM HEPES pH 7.4, 10 mM KCl, 20 mM MgCl_2_, 1 mM EDTA] was incubated with 50 µg of the P_10_-mitochondria-enriched fraction in the presence or absence of C_16_-ceramide in 50 µl MSB buffer [400 mM mannitol, 50 mM Tris-HCl pH 7.2, 4 mM MgCl_2_, 10 mM KH_2_PO_4_, 50 µM rotenone, 5 mM succinate, 5 mg/ml fatty acid free bovine serum albumin (BSA; Sigma)] for 30 min or 1 h at 30°C. Reaction samples were centrifuged at 14,000×*g* for 5 min, and supernatants and mitochondrial pellets were analyzed for cytochrome *c* release. For measurement of Bax insertion, mitochondrial pellets were subjected to alkali extraction as described above and alkaline-sensitive and alkaline-resistant fractions were analyzed by immunoblotting using anti-Bax or anti-GST antibodies.

### Isolation of mitochondria from mouse liver

Mouse liver mitochondria were isolated as described [16] with modification. Livers were surgically removed from 8 week old C57BL/6 black mice (Jackson Laboratory, Bar Harbor, ME) sacrificed by CO_2_ asphyxiation under protocol 09-08-016 in compliance with the guidelines of the Institutional Animal Care and Use Committee (IACUC) of MSKCC. Briefly, isolated liver tissue was washed twice and minced in ice-cold Buffer A (0.25 M sucrose, 10 mM HEPES pH 7.4, 0.5 mM EGTA). Minced tissue was then re-washed in Buffer A and homogenized (0.3 g of liver/ml of buffer) on ice for 3 min using a glass homogenizer with a loose-fitting pestle. Homogenates were centrifuged at 600×*g* for 15 min, and the resulting pellets containing nuclei and undisrupted cells were discarded. Supernatants were centrifuged at 9750×*g* for 15 min, and the resulting pellet was resuspended in Buffer A and centrifuged at 9750×*g* for 10 min. The pellet was washed 3 more times in Buffer A and once in Buffer B (0.25 M sucrose, 10 mM HEPES pH 7.4). The final mitochondrial pellet was resuspended in Buffer B at 50 mg protein/ml. All steps were carried out at 0–4°C. Mice were housed at the animal core facility of Memorial Sloan-Kettering Cancer Center. This facility is approved by the American Association for Accreditation of Laboratory Animal Care and is maintained in accordance with the regulations and standards of the United States Department of Agriculture and the Department of Health and Human Services, National Institutes of Health. The Institutional Animal Care and Use Committee at Memorial Sloan-Kettering Cancer Center approved these mice experiments under Protocol # 09-08-016.

### Uptake of C_16_-ceramide or C_16_-dihydroceramide by isolated mouse liver mitochondria

Isolated mitochondria (1 µg/µl) were incubated with C_16_-ceramide or C_16_-dihydroceramide (0–5 µM; 1% final solvent concentration) in a KCl-based medium [20 mM HEPES, pH 7.4, 150 mM KCl, 25 mM NaHCO_3_, 1 mM MgCl_2_, 3 mM NaH_2_PO_4_, 250 mM sucrose, 1 mM glutamate/malate, 5 mg/ml fatty acid free BSA] for 5 min at 37°C. Thereafter, samples were centrifuged at 10,000×*g* for 10 min at 4°C, the mitochondrial pellet was resuspended in 1×PBS buffer, and ceramide levels in mitochondria (600 µg) were quantified by the diacylglycerol kinase assay.

### Formation of MCRMs within and cytochrome *c* release from mouse liver mitochondria ex vivo

Recombinant full length Bax or BaxΔC in Bax dilution buffer was incubated with or without C_16_-ceramide with isolated mitochondria (1 mg/ml) in KCl-based medium for 5 or 15 min at 37°C. Reaction samples were centrifuged at 14,000×*g* for 5 min at 4°C and the supernatant containing proteins released from isolated mitochondria and pellet containing retained mitochondrial proteins were analyzed for cytochrome *c*. For MCRM isolation, the mitochondrial pellet was subjected to 5–30% continuous sucrose density gradient after incubation with 0.05% Triton X-100 as below.

### Isolation of MCRM by sucrose density gradient

Isolation of HeLa and mouse liver MCRM was based on concepts derived from isolation of detergent-insoluble raft-like plasma membrane macrodomains [43]. Identification of HeLa and mouse liver MCRM initially involved extensive detergent dose titration. Mitochondria (3.3 mg/ml), isolated in SHE buffer, were incubated on ice for 30 min with Triton X-100 (0.01–1%) or CHAPS (0.1–2%), and solubilized mitochondrial proteins were separated from insoluble proteins by centrifugation at 14,000×*g* for 5 min at 4°C. Extent of solubilization of the inner mitochondrial membrane protein COXIV was confirmed by Western blot. The lowest concentrations that induced complete COXIV solubilization, 0.05% Triton X-100 for mouse liver mitochondria and 0.15% Triton X-100 or 1% CHAPS for HeLa mitochondria, were selected for MCRM isolation. For these studies, mitochondria (3.3 µg/µl) were treated on ice with detergent for 30 min in 1×MBS buffer (25 mM MES pH 6.5, 150 mM NaCl), followed by homogenization with 20 strokes of a loose-fitting dounce homogenizer or by sonication (3×10 sec). The homogenate was adjusted with 80% sucrose in MBS buffer to 40% final sucrose concentration and transferred to a 12 ml ultracentrifuge tube. A continuous gradient consisting of 4 ml of 5% and 6 ml of 30% sucrose, or a discontinuous gradient consisting of 5 ml of 5% and 5 ml of 30% sucrose was layered on top of the homogenate, and samples were ultracentrifuged at 182,000×*g* for 16 h in Beckman SW41 rotor at 4°C. As with isolation of plasma membrane platforms, this procedure yielded an opaque detergent insoluble band at fraction 5–7 of the gradient. One ml fractions were collected from atop the gradient and profiled for proteins by Western blotting after 20% TCA precipitation, and for lipids as in Experimental Procedures. For mini-discontinuous sucrose gradient, 80 µl of homogenates adjusted to 40% sucrose were transferred to an ultracentrifuge tube and overlaid successively with 80 µl of 30% sucrose and 80 µl of 5% sucrose. Samples were centrifuged at 182,000×*g* for 18 h in Beckman TLA 100 rotor at 4°C, and three 80 µl fractions were collected from the top. Pellets were resuspended in 80 µl MBS by brief sonication.

### Staining and visualization of MCRMs

MitoTracker was used to outline mitochondrial anatomical boundaries for ease of co-localization, and hence cannot bestow an MCRM artifact. In studies examining MCRM formation upon radiation in HeLa cells, mitochondria were stained by incubating live cells with 100 nM MitoTracker Deep Red 633 for 30 min at 37°C as per manufacturer instructions before mitochondrial isolation. However, for *ex vivo* studies with isolated mitochondria examining the effects of ceramide and Bax on MCRM formation, the isolated mitochondria were stained either before or after fixation. When staining live mitochondria prior to fixation, mitochondria were pre-incubated with 200 nM MitoTracker Deep Red 633 in 2.5% FBS and 2.5% goat serum in PBS for 1 h on ice. MitoTracker is an organic dye with a charge that directs its binding to membranes with a potential, i.e. live mitochondria. However, its hydrophobic nature also permits binding membranes lacking a potential after fixation, if incubation is prolonged. Therefore, the fixed staining approach was used for the majority of *ex vivo* experiments for ease of experimental procedure. For staining of fixed mitochondria, mitochondria were fixed for 45 min with 2% formaldehyde, pelleted by centrifugation at 6000×*g* for 5 min, followed by brief washing with PBS, and incubation with 200 nM MitoTracker Deep Red 633 in 2.5% FBS and 2.5% goat serum in PBS for 16 h at 4°C. Mitochondria, stained with MitoTracker, were washed once in Wash Buffer (1% FBS, 0.025% Tween 20 in 1×PBS), and incubated with anti-ceramide (Alexis, MID15B4; 1∶30) and/or anti-Bax (6A7; 1∶50, Calbiochem or Δ21; 1∶10, Santa Cruz) antibodies in Wash Buffer for 45 min. Mitochondria were then washed, followed by incubation with Cy3-conjugated goat anti-mouse IgM (Jackson ImmunoResearch; 1∶100) and/or Cy2-conjugated goat anti-mouse IgG (Jackson ImmunoResearch; 1∶100) or Alexa Fluor 488-conjugated goat anti-rabbit IgG (Molecular Probes; 1∶100) secondary antibodies, respectively. After 45 min, mitochondria were washed 3 times, and then once in 0.025% Tween 20 in PBS. All staining steps were at 4°C. After staining, mitochondrial pellets were resuspended in 20 µl 0.025% Tween 20 in PBS. 5 µl of mitochondrial suspension was spread on slide using a cover slip and mounted using the ProLong Gold Antifade Reagent (Invitrogen). Images were acquired with a Leica TCS AOBS SP2 confocal microscope equipped with a 100×1.4 NA OIL DIC D Objective combined with 2× scan zoom. Co-localization analysis was performed with MetaMorph 7.5 software.

### Statistical Analysis

Statistical analysis was performed by Student's *t*-test or linear regression analysis with 95% confidence estimation for ED_50_.

## Results

### Mitochondrial ceramide elevation precedes Bax activation in irradiated HeLa cells

**Figure 1:**
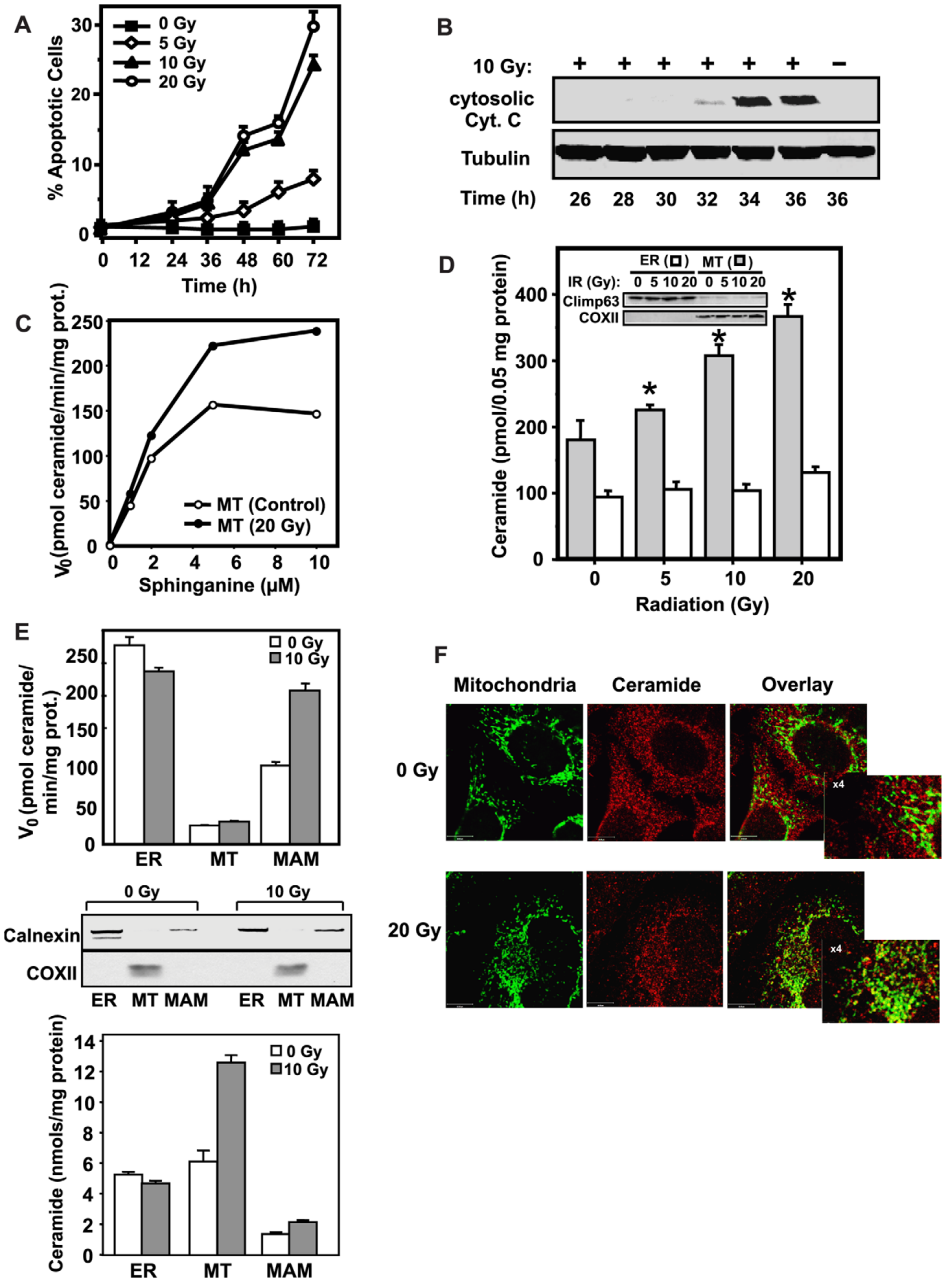
Ionizing radiation-induced ceramide elevation is confined to mitochondria. (A) Time- and dose-dependent induction of apoptosis in response to ionizing radiation. Morphologic changes of nuclear apoptosis were detected using the DNA-specific fluorochrome *bis*-benzimide. Data (mean±SEM) are collated from 3 experiments analyzing 500 cells per point. (B) Time-dependent cytochrome *c* (Cyt. *c*) release after 10 Gy. Cytosolic fractions of HeLa cells, collected at the indicated times post-irradiation were analyzed by immunoblotting using α-Cyt. *c*. Data are from 1 representative of 3 studies. (C) Increased CS activity in isolated mitochondria (MT) after 20 Gy. CS activity was measured at 28 h post-irradiation. Data are from 2 experiments. (D) Radiation increases mitochondrial ceramide content. At 33 h post-irradiation, ceramide was quantified by the diacylglycerol kinase assay in mitochondrial-enriched and ER-enriched fractions from HeLa cells. Inset: The ER-enriched fraction was devoid of mitochondrial contamination based on western blotting with anti-COXII (mitochondrial marker), while the mitochondrial fraction was 10.9±1.7% (mean±SE) ER based on anti-Climp63 (ER marker) blotting. Data (mean±SE) are from 4 experiments performed in triplicate. *, p<0.05 vs. control. (E) Ionizing radiation increases CS activity in MAM and ceramide levels in MAM-free mitochondria (MT). HeLa cells were harvested 33 h post-10 Gy and organelles isolated as in Materials and Methods. ER (P100) was fractionated by differential centrifugation and mitochondria and MAM within the heavy membrane fraction (P10) were further separated from each other by 30% Percoll gradient. Upper panel; CS activity was measured in each fraction using sphinganine and palmitoyl-CoA as substrates as in Materials and Methods. Middle panel: purity of ER, Mitochondria and MAM fractions was analyzed by immunoblotting with antibodies to Calnexin (ER marker) and COXII (MT marker). Based on anti-Calnexin blotting, Percoll-purified mitochondria were 3–4% contaminated with ER. Lower panel; Ceramide levels were quantified by diacylglycerol kinase assay as in Materials and Methods. (F) Co-localization of mitochondrial ceramide and COXI after 20 Gy. At 33 h post-irradiation, HeLa cells were stained with anti-ceramide IgM (red) and anti-COXI IgG (green). Images were acquired with a Leica TCS AOBS SP2 confocal microscope equipped with a 63×1.4NA OIL DIC D objective combined with 4× scan zoom, and co-localization (yellow) was analyzed with MetaMorph 7.5 software. Scale bar; 10 µm. Inset images (rectangles) represent 4× magnification of left upper (0 Gy) and left lower (20 Gy) regions for observation of co-localization. Data are from 1 of 5 experiments.

**Figure 2:**
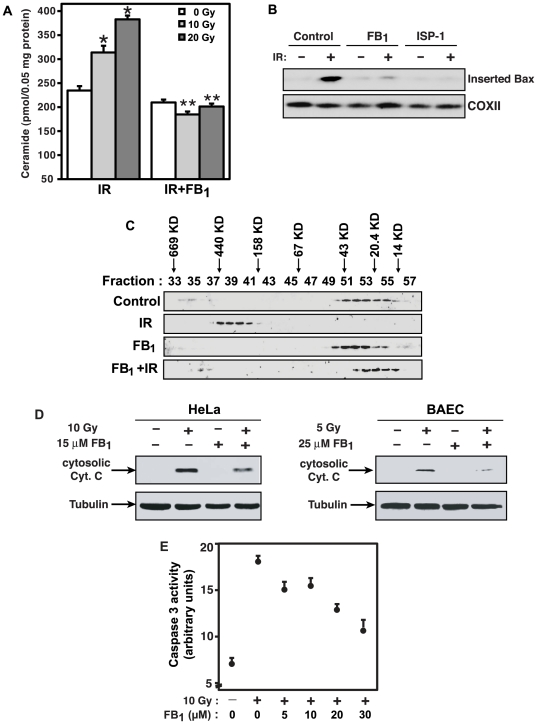
FB_1_ prevents radiation-induced MOMP in HeLa cells. (A) FB_1_ blocks mitochondrial ceramide generation. HeLa cells were irradiated (IR) and treated with 15 µM FB_1_ 20 h post-irradiation. Ceramide in isolated mitochondria was quantified by diacylglycerol kinase assay at 36 h post-irradiation. Data (mean±SEM) are from 2 experiments performed in triplicate. *, p<0.05 vs. control; **, p<0.01 vs. irradiated. (B) FB_1_ prevents radiation-induced Bax insertion into the MOM. Alkali-resistant mitochondrial fractions containing inserted Bax were isolated after 34 h from HeLa cells irradiated with 20 Gy and treated with 25 µM FB_1_ or 75 nM ISP-1 at 20 h post-irradiation. COXII was used as mitochondrial loading control. Data are from 1 of 4 studies. (C) FB_1_ blocks radiation-induced Bax oligomerization. At 34 h post-irradiation, mitochondrial proteins from HeLa cells treated as in (B) were separated by gel filtration. Data are from 2 studies. (D) FB_1_ attenuates radiation-induced cytochrome *c* release in HeLa cells and BAEC. HeLa cells were irradiated with 10 Gy, and 15 µM FB_1_ was added 20 h post-irradiation. BAEC cells were treated with 25 µM FB_1_ 1 h before irradiation with 5 Gy. 36 h (HeLa) and 12 h (BAEC) post-irradiation, cytosolic fractions were analyzed by immunoblotting using mouse monoclonal anti-Cyt. *c* and mouse monoclonal anti-tubulin antibodies. Data are from 1 of 2 studies in HeLa and BAEC each. (E) FB_1_ attenuates radiation-induced caspase activity. FB_1_ was added to cells 20 h after 10 Gy and caspase activity measured at 36 h post-irradiation using the fluorogenic caspase substrate Z-DEVD-AFC. Data (mean±SEM) are from 1 of 2 investigations performed in triplicate.

Morphologic evidence of apoptosis was detected in 5–15 Gy irradiated HeLa cells by 36 h and became significant at 48 h (Figure 1A; p<0.001 vs. control). Increased cytosolic effector caspase 3 activity, measured fluorometrically by cleavage of the substrate Z-DEVD-AFC, was similarly detected by 30 h post-irradiation, peaking at 36 h, remaining elevated for at least 72 h after 10 Gy (see Figure 2E for 36 h data). Cytochrome *c* release from mitochondria into cytosol was detected at 30–32 h after 10 Gy by Western analysis (Figure 1B) and was maximal at 36 h. Consistent with the cytochrome *c* release profile, Bax insertion into the MOM also began at 30 h, peaking at 34 h after 10 Gy (Figure S1A). In agreement with previous studies [44], Bax insertion occurred without apparent translocation from cytosol (not shown), but rather from a Bax pool loosely attached to unirradiated HeLa mitochondria but not inserted into MOM [37], [45]. This pool, which is readily removed by 100 mM Na_2_CO_3_ washing and does not spontaneously induce MOMP [45], [46], represents the large majority of endogenous Bax co-isolated with HeLa mitochondria (estimated at ∼80% in our preparations based on semi-quantitative densitometry normalized for protein recovery; n = 8). At 34 h post-irradiation, the fraction of Bax inserted into the MOM had increased 4–5-fold (Figure S1A, p<0.001 vs. control unirradiated, n = 6). Bax multimerization into low molecular weight (not shown) and higher molecular weight oligomers (M_r_ 360 KD) occurred concomitant with Bax insertion (Figure S1B). Whereas mitochondrial Bax was primarily monomeric in unirradiated HeLa cells (n = 4), a majority of Bax (estimated at ∼60% of the total by densitometry) redistributed into high molecular weight oligomeric fractions post-irradiation (Figure S1B). In contrast, Bak and VDAC were found in both fractions in unirradiated controls, and as previously reported [37] redistributed minimally upon irradiation (Figure S1B).

Ceramide elevation preceded Bax activation. Ceramide levels increased up to 2.6-fold of baseline (350 pmols/10^6^ cells) beginning at 28 h post-20 Gy and remained elevated until at least 36 h post irradiation (not shown; p<0.005 at all times vs. control). Whereas sphingomyelinase activity was not elevated post-irradiation, similar to published data [47], CS activity increased in the post-nuclear membrane fraction (P_100_) at 28 h in the current studies and remained elevated until at least 32 h [47]. Ionizing radiation increased CS maximal reaction velocity (V_max_) of the mitochondrial-enriched fraction specifically, as determined by Michaelis-Menten kinetics (Figure 1C), whereas ER CS activity was not increased (see Figure 1E). Consistent with this observation, the cellular ceramide increase upon irradiation appeared confined to the mitochondrial-enriched fraction, with no change in irradiated ER (Figure 1D).

Previous studies showed mitochondria prepared by differential centrifugation on discontinuous sucrose gradients co-purify with mitochondrial-associated membrane (MAM), an ER-like structure that links to mitochondria via specific MOM contact sites and acts as a conduit for transfer of ER and/or MAM metabolites into mitochondria [48]. We, therefore, separated MAM from mitochondria to further explore the site of radiation-induced CS activity. Precedence exists for transfer of lipids from MAM to MOM as phosphatidylserine (PS), synthesized exclusively in MAM via PS synthases, is transported to MOM, then the inner mitochondrial membrane, where it serves as the sole source of mitochondrial phosphatidylethanolamine, via decarboxylation [49], [50]. Here we show that HeLa cell MAM, but not ER or mitochondria, is the exclusive site of radiation-induced CS activation (Figure 1E, upper panel) with apparent subsequent transfer of ceramide to mitochondria (Figure 1E, lower panel), as 75% of the ceramide mass increase is observed within MAM-free mitochondria. Similar transfer of MAM ceramide to MOM was recently reported by Colombini and co-workers [51]. In contrast, the level of total mitochondrial phospholipid and of sphingosine-1-phosphate, a ceramide metabolite with second messenger function, remained unchanged (not shown). A similar high baseline ceramide concentration and stimulated elevation were recently detected by mass spectroscopy in MAM-free mitochondria isolated from untreated and phorbol ester-treated MCF-7 breast cancer cells [52].

Further evidence for preferential ceramide elevation in mitochondria post-irradiation was derived from confocal microscopic double immunostaining using anti-ceramide IgM, and anti-COXI IgG as mitochondrial marker (Figure 1F). The anti-ceramide antibody used is highly specific, distinguishing C_16_-ceramide from C_16_-dihydroceramide (Figure S2), which differs from ceramide by a double bond in the sphingoid base backbone. While only 18.4±2.7% (mean±SE) of ceramide co-localized with mitochondria (Figure 1F, upper panels, yellow merged signal) in unirradiated cells, the level of ceramide co-localized to mitochondria increased to 31.2±3.3% (Figure 1F, bottom panels) (p<0.001; n = 20) at 33 h post-irradiation. Similar co-localization was displayed with Mitotracker (not shown). Alternately, no radiation-induced co-localization of anti-ceramide with ER-marker (anti-KDEL) was detected (Figure S3). Taken together, these studies indicate that ceramide increases preferentially within HeLa cell mitochondria during evolution of radiation-induced apoptosis.

### The natural CS inhibitor, Fumonisin B_1_, inhibits MOMP

Fumonisin B_1_ (FB_1_) is a natural competitive CS inhibitor [53] with an IC_50_ two orders of magnitude left-shifted to the K_m_ for sphingoid base acylation [53]. A short treatment of HeLa cells with 15 µM FB_1_, beginning 20 h post-irradiation, did not affect basal ceramide levels but abolished radiation-induced mitochondrial ceramide elevation (Figure 2A). FB_1_ also reduced Bax insertion into the MOM (Figure 2B). In five experiments, FB_1_ inhibited Bax insertion by 83±5% (p<0.01 vs. irradiated) and nearly completely inhibited formation of high molecular weight Bax oligomers (Figure 2C). Inhibition of radiation-induced Bax insertion and oligomerization by FB_1_ reduced cytochrome *c* release into cytosol by 72% (Figure 2D, left panel). Similar reduction was observed in bovine aortic endothelial cells (BAEC) (Figure 2D, right panel), previously reported to undergo radiation-induced CS activation [54]. Consequently, FB_1_ also attenuated caspase 3 activation (67% at 30 µM; Figure 2E) and inhibited apoptosis assessed by *bis*-benzimide staining in HeLa cells (not shown) and BAEC [54]. Similar results were obtained using ISP-1 (Figure 2B and not shown), an inhibitor of serine palmitoyl transferase, the enzyme catalyzing synthesis of the sphingoid base substrate for *de novo* ceramide synthesis via CS [55]. These data define a role for ceramide generated via CS in the mitochondrial phase of radiation-induced apoptosis in HeLa cells.

### C_16_-ceramide activates Bax within isolated HeLa mitochondria

**Figure 3:**
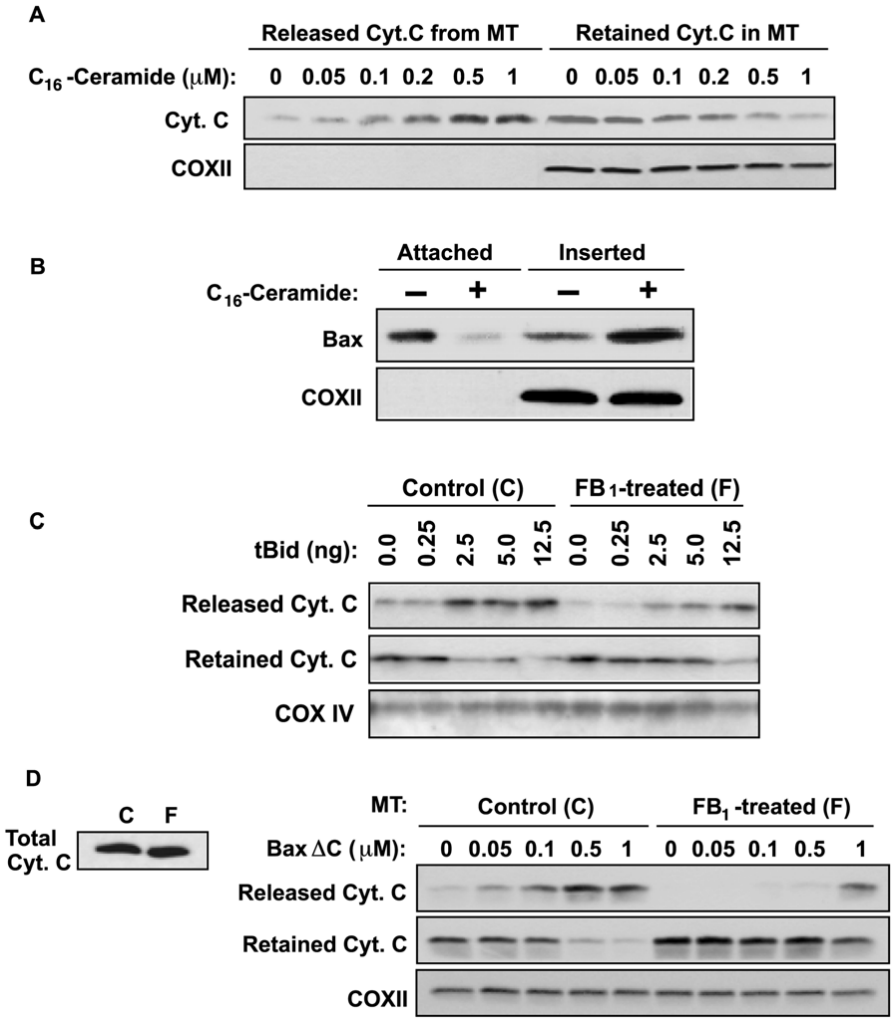
Effect of C_16_-ceramide on MOMP in isolated HeLa mitochondria. (A) Ceramide induces cytochrome *c* release from isolated HeLa mitochondria. C_16_-ceramide (0–1 µM) was incubated with HeLa mitochondria (1 µg/µl) in MSB buffer. After 1 h at 30°C, samples were centrifuged at 14,000×*g* for 5 min at 4°C to separate released (supernatant) and retained (pellet) mitochondrial proteins, and analyzed for cytochrome *c* release by immunoblotting using anti-Cyt.*c* and anti-COXII as loading control. Data are from 1 of 4 investigations. (B) Ceramide induces insertion of endogenous Bax into HeLa mitochondrial membranes. Isolated mitochondria were incubated with 1 µM C_16_-ceramide and mitochondrial pellets were collected after incubation as in (A). Attached and inserted Bax were separated by alkali extraction of mitochondrial pellets as in Figure 2B and analyzed by Western blot with anti-Bax and anti-COXII as loading control. Data are from 1 of 4 investigations. (C) FB_1_ inhibits tBid-induced cytochrome *c* release. Isolated HeLa mitochondria were incubated with 0.25–12.5 ng of caspase-8 cleaved human Bid for 30 min and cytochrome *c* release was analyzed as in (A). Data represent one of three similar studies. (D) FB_1_ inhibits BaxΔC-induced cytochrome *c* release. HeLa mitochondria, replete (control) or depleted of ceramide (from 35 µM FB_1_-pretreated cells), were incubated with BaxΔC (0–1 µM) for 30 min and cytochrome *c* release was analyzed as in (A). The left panel shows the cytochrome *c* content of mitochondria isolated from FB_1_-pretreated cells was not different than that from untreated HeLa cells. Data are from 1 of 5 investigations.

To provide direct evidence that ceramide effects Bax-mediated MOMP, isolated HeLa mitochondria were treated with natural C_16_-ceramide (0.05–1 µM), resulting in dose-dependent cytochrome *c* release (Figure 3A). In contrast, treatment with anti-apoptotic sphingosine-1-phosphate (0.05–1 µM) failed to induce cytochrome *c* release under the same conditions (not shown). 1 µM C_16_-ceramide also rendered the endogenous Bax known to co-purify with HeLa mitochondria (termed attached Bax; [37], [46]) resistant to alkali extraction (Figure 3B), indicating attached Bax had inserted into the MOM. The ED_50_ for cytochrome *c* release of approximately 0.2 µM C_16_-ceramide (Figure 3A) and peak response at 0.5 µM (Figure 3A) were right-shifted from the ED_50_ for Bax insertion (approximately 0.05 µM C_16_-ceramide with maximal insertion at 0.12 µM) (Figure S4).

To provide additional evidence for ceramide function in Bax insertion, HeLa cells were pre-treated for a prolonged time (24 h) with high FB_1_ doses (35–50 µM), reducing baseline mitochondrial ceramide content from 250±40 to 80±40 pmols/0.05 mg mitochondrial protein (mean±SD) without affecting mitochondrial cytochrome *c* content (Figure 3D, left panel). Sensitivity of ceramide-depleted HeLa mitochondria towards tBid-induced cytochrome *c* release, which depends on insertion/activation of the endogenous Bax associated with isolated mitochondria [44], was markedly reduced (Figure 3C). Consistent with published literature [44], tBid-induced dose-dependent insertion of attached endogenous Bax into the MOM (not shown) and cytochrome *c* release, which was maximal at 2.5 ng tBid/50 µg mitochondrial protein (Figure 3C, left). tBid-induced cytochrome *c* release from ceramide-depleted mitochondria, however, was attenuated at least 5-fold (Figure 3C, right), accompanied by inhibition of Bax insertion (not shown).

As there are two distinct events in the Bax activation process in which lipids direct Bax insertional activation, the initial tethering of Bax to the MOM via the α9 C-terminal helical domain and the activation of the lipidic pore through the α5-α6 hairpin, studies were designed to evaluate ceramide impact specifically on the lipidic pore. For these studies we employed BaxΔC, which lacks amino acids 172–192 including the C-terminal TM domain [56]. Similar to what we observed with tBid, cytochrome *c* induced release by BaxΔC was reduced >1 log in ceramide-depleted mitochondria compared to control HeLa mitochondria (Figure 3D). Ceramide depletion also blocked Bax insertion into the MOM of mitochondria isolated from FB_1_-treated BAEC (not shown), and prevented cytochrome *c* release therefrom (Figure S5, lower panel). Together these data indicate ceramide is a significant regulator of Bax-mediated MOMP.

### Ceramide induces formation of a mitochondrial ceramide-rich macrodomain (MCRM)

**Figure 4:**
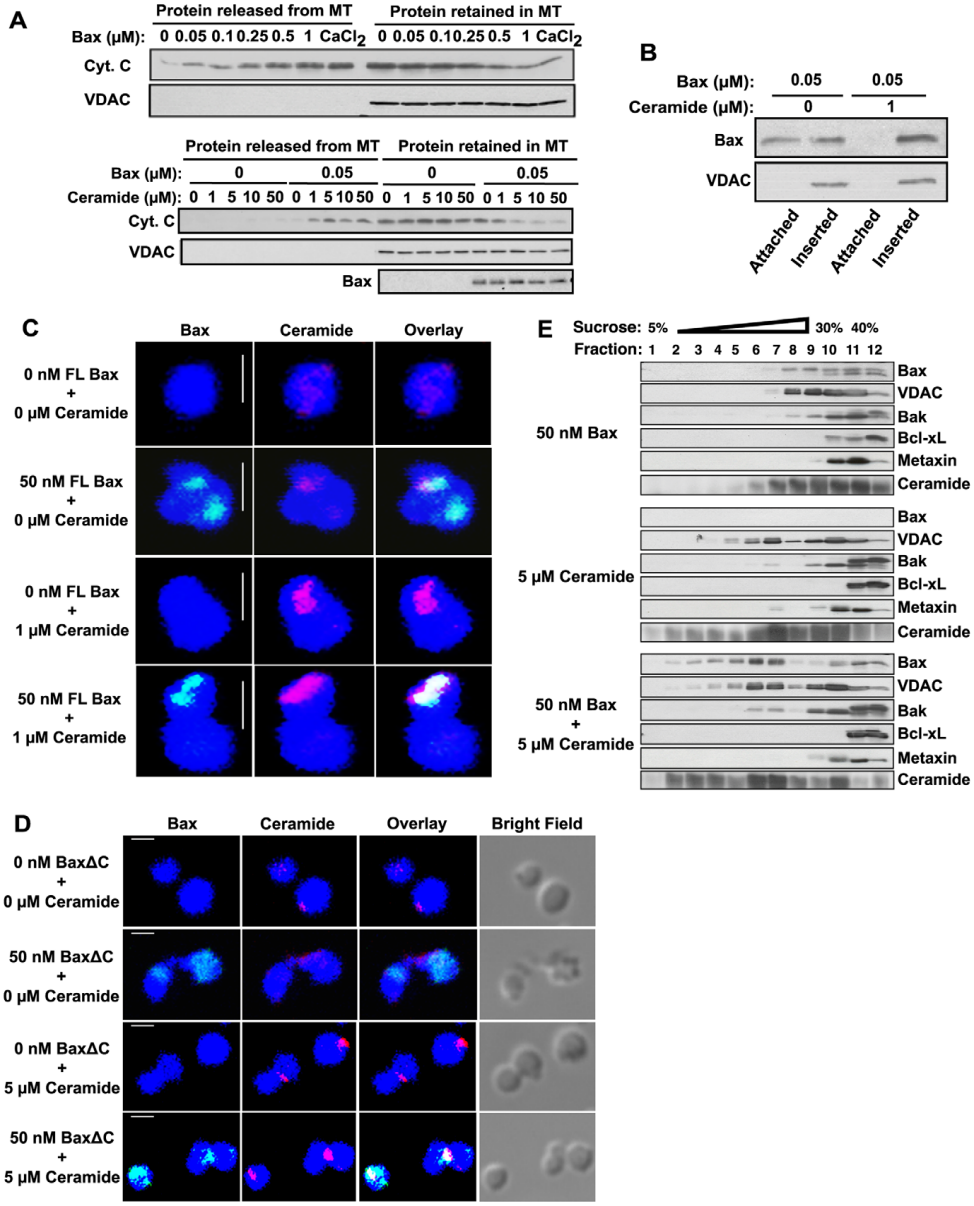
Ceramide is non-apoptogenic by itself and facilitates Bax-induced MOMP. (A) Ceramide facilitates Bax-induced cytochrome *c* release from isolated mouse liver mitochondria *ex vivo*. Recombinant BaxΔC (0–1 µM, upper panel), or C_16_-ceramide (0–50 µM) plus 0.05 µM BaxΔC (lower panel) were incubated with isolated mouse liver mitochondria (1 µg/µl) in KCl buffer for 5 min at 37°C, supernatants and pellets were separated by centrifugation at 14,000×*g* for 5 min at 4°C, and cytochrome *c* release was measured as in Figure 3A. The outer mitochondrial membrane protein VDAC was used as loading control. Data are from 3 studies. (B) Ceramide induces insertion of recombinant BaxΔC into isolated mouse liver mitochondria. Mitochondria were treated as in (A) and attached and inserted BaxΔC analyzed as in Figure 3B. Data are from 2 independent studies. (C) Full-length (FL) Bax co-localizes with MCRMs induced by exogenous C_16_-ceramide. Mouse liver mitochondria were incubated in MSB-based medium in the presence or absence of 50 nM full-length Bax for 5 min at 30°C. Thereafter, 0 or 1 mM C_16_-ceramide [1% final solvent concentration (ethanol∶dodecane, 98∶2 v/v)] was added to the mixture for an additional 10 min. Mitochondria were fixed and stained with MitoTracker (blue), and ceramide and Bax were localized using anti-ceramide IgM (red) or anti-Bax IgG (green), respectively. Images, acquired with a Leica TCS AOBS SP2 confocal microscope equipped with a 100×1.4NA OIL DIC D objective combined with 2× scan zoom, were analyzed with MetaMorph 7.5 software. Control IgM and IgG did not yield detectable signals (not shown). Scale bar; 1 µm. Data represent 1 of 3 similar studies. (D) BaxΔC co-localizes with MCRMs induced by exogenous C_16_-ceramide. Experiments were performed as in (C) using 5 mM C_16_-ceramide [1% final solvent concentration (ethanol∶dodecane, 98∶2 v/v)] in the presence or absence of 50 nM recombinant BaxΔC. Scale bar; 1 µm. Data represent 1 of 4 similar studies. (E) Biophysical isolation of mouse liver MCRMs. After incubation with 50 nM BaxΔC and 5 µM C_16_-ceramide, mitochondria (3.3 mg/ml), pelleted as in Figure 4A were resuspended in cold MBS buffer containing 0.05% Triton X-100. After 30 min on ice, mitochondria were homogenized with 20 strokes of a loose-fitting dounce homogenizer. The mitochondrial homogenate was adjusted to 40% final sucrose concentration and subjected to 5–30% continuous sucrose density gradient as in Materials and Methods. 400 ml of each 1 ml fraction were concentrated by 20% TCA precipitation, and proteins were resolved on a 15% SDS-PAGE gel and identified by immunoblot analysis using antibodies to the indicated proteins. Ceramide was measured using 400 µl aliquots by diacylglycerol kinase assay. Data are from 3 independent studies.

The above studies are consistent with either mitochondrial ceramide acting, like Bax, to directly release cytochrome *c*, or alternatively as a facilitator of Bax action. To resolve this issue, we employed mouse liver mitochondrial preparations, which, in contrast to HeLa mitochondria, are isolated without attached Bax [46] (Figure S6). Hence, they can serve as a model to examine autonomous ceramide action. Treatment of mouse hepatic mitochondria with recombinant BaxΔC induced concentration-dependent cytochrome *c* release (Figure 4A, upper panel). As little as 0.05 µM BaxΔC was effective, and 1 µM BaxΔC was maximal, and equivalent to 150 µM CaCl_2_, a maximally-effective concentration for permeability transition-induced release [46]. Neither stimulus impacted the content of the MOM protein VDAC (Figure 4A, upper panel, lower lane) or the soluble matrix protein Hsp-60 (Figure S7), indicating specificity of the cytochrome *c* release process. In contrast, C_16_-ceramide alone had no impact on cytochrome *c* release up to 50 µM (Figure 4A, lower panel), consistent with ceramide not functioning as a non-specific “detergent” effecting cytochrome *c* release [note ceramide is a non-swelling amphiphile and hence by definition not a detergent [57]]. Furthermore, when C_16_-ceramide was added to mouse hepatic mitochondria prior to 0.05 µM BaxΔC, a minimally-effective dose for BaxΔC alone, cytochrome *c* release was dramatically enhanced. Combination of 1–5 µM C_16_-ceramide and 0.05 µM BaxΔC yielded cytochrome *c*-releasing capacity equivalent to 1 µM BaxΔC alone, a dose-modifying factor of 20-fold. This effect appeared specific for ceramide, as the ceramide precursor C_16_-dihydroceramide was ineffective (Figure S8). Further, the profile of proteins released from mitochondria in response to 50 nM BaxΔC plus C_16_-ceramide was nearly identical to that released by 1 µM BaxΔC alone measured by Coomassie blue staining (Figure S9) and confirmed by mass spectrometry, consistent with ceramide acting via the Bax pore. Similar results were obtained combining C_16_-ceramide with full-length recombinant Bax albeit with lower efficacy for cytochrome *c* release (not shown), confirming published data using isolated rat liver mitochondria [15].

Enhanced BaxΔC function in the presence of 1–5 µM C_16_-ceramide, which increased mitochondrial ceramide content only 2–3 fold (Figure S8), appeared to reflect both increased Bax insertion and effectiveness. Figure 4B shows that at 0.05 µM BaxΔC, half the mitochondrial-associated BaxΔC is inserted, and that C_16_-ceramide confers complete insertion, effectively doubling the quantity of inserted BaxΔC. However, as the dose-modifying factor for cytochrome *c* release is 20-fold, these studies insinuate increased apoptogenic effectiveness of inserted Bax in the presence of ceramide. In contrast, C_16_-dihydroceramide did not impact BaxΔC insertion (Figure S8). Hence, ceramide is not an autonomous apoptotic factor *per se* but rather serves to enhance mitochondrial Bax insertion and function.

To ascertain whether ceramide elevation in mitochondria results in formation of a ceramide-rich macrodomain into which Bax inserts and oligomerizes, we employed confocal microscopy using intact mitochondria isolated from mouse liver. Detection of MCRMs and co-localization of ceramide with Bax were performed using Cy3 and Cy2, probes that emit at 566 nm and 510 nm, respectively. Co-localization analysis of confocal microscopic images can be affected by the resolution limit, defined by the standard equation: 0.4×λ_em_/NA = R (λ_em_: emission wavelength, R: resolution, NA: numerical aperture; 1.4 in this study). Based on this equation, the calculated resolutions for Cy3 and Cy2 are 162 nm and 146 nm, respectively, readily permitting co-localization analysis using these probes in ≈1 µM-sized mitochondria.

**Figure 5:**
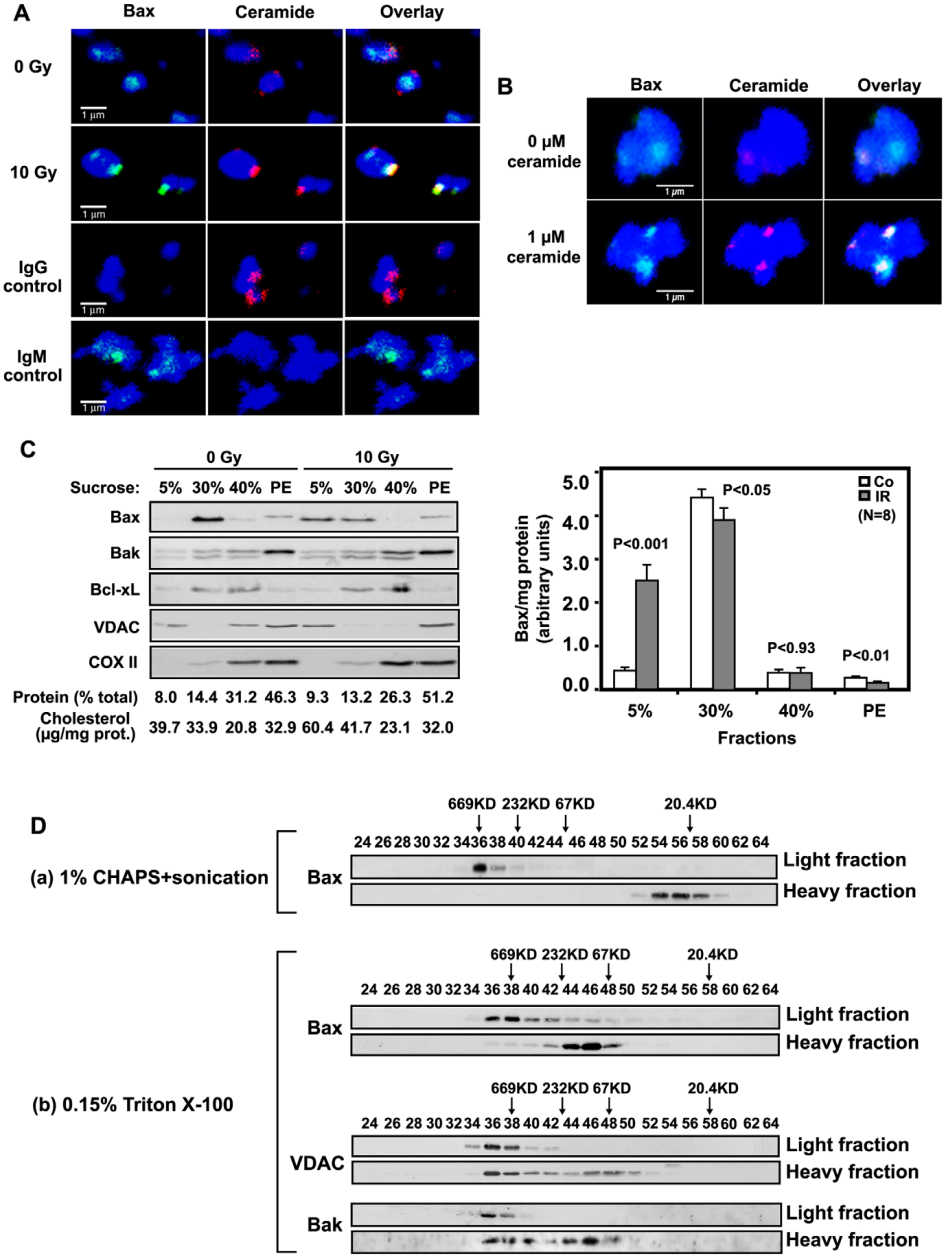
Ceramide induces formation of a mitochondrial ceramide-rich macrodomain (MCRM). (A) Ionizing radiation (10 Gy) induces co-localization of endogenous Bax with MCRMs in HeLa cells. Mitochondria were isolated from HeLa cells 34 h after irradiation and immunostained as described in Supporting Information Text S1. Data represent typical stainings from 1 of 4 similar studies in which 2000 mitochondria were analyzed each. (B) Addition of exogenous C_16_-ceramide induces co-localization of endogenous full-length Bax with MCRMs in HeLa cells. Mitochondria were isolated from HeLa cells using percoll gradient and treated with ceramide as Figure 3A. After 30 min incubation, mitochondria were fixed and stained with MitoTracker (blue), while ceramide and Bax were localized using anti-ceramide IgM (red) or anti-Bax IgG (green), respectively. Control IgM and IgG did not yield detectable signals (not shown). These data represent 1 of 3 similar studies. (C) Bax translocates into a radiation-generated HeLa MCRM. Upper panel: 34 h post-irradiation, HeLa mitochondria were isolated as in Materials and Methods and incubated with 0.15% Triton X-100 in MBS buffer for 30 min on ice. 40 µl mitochondrial homogenate (3.3 µg/µl) were subjected to 5–30% mini-discontinuous sucrose density gradient centrifugation as described in Materials and Methods. 20 µl aliquots of 80 µl fractions were analyzed by immunoblotting using the indicated antibodies. The protein level of each fraction was assessed using the Bio-Rad D_c_ protein assay kit (PE, Pellet). Data are from 1 of 4 studies, consisting of 2 independent gradients per study. The gradient shown displays our clearest example of Bax translocation into light membranes. Lower panel: Bax in each fraction, revealed by immunoblotting and quantified using NIH Image software, was normalized to protein content for all 8 gradients. (D) MCRM Bax exists as high molecular weight oligomers. Mitochondria from 10 Gy-irradiated HeLa cells, disrupted by either (a) 1% CHAPS and sonication or (b) dounce homogenization in 0.15% Triton X-100, were subjected to 5–30% discontinuous sucrose gradient for MCRM isolation as in Experimental Procedures. Light (MCRM; fractions 6,7) and heavy fractions (solubilized proteins; fractions 11,12) were analyzed by gel filtration on Sephacryl S-200 column as in Figure 2C. 500 µl of each eluted fraction were concentrated by 20% TCA precipitation for immunoblotting. Data are from 3 independent studies.

After addition of C_16_-ceramide, and/or recombinant full-length (FL) Bax (Figure 4C) or BaxΔC (Figure 4D), mitochondria were stained with MitoTracker (blue), and mitochondrial ceramide and Bax were localized using anti-ceramide IgM (red) or anti-Bax IgG (green), respectively. Control IgM and IgG did not yield detectable signals (see Figure 5A). Consistent with the Western analysis (Figure S6), Figures 4C and 4D show that endogenous Bax was not immunodetected in naïve mitochondria (top lanes), while barely detectable ceramide-containing domains were observed at low incidence (top lanes). Very little added recombinant full length Bax or BaxΔC co-localized with these small ceramide-rich domains (second lanes in Figures 4C and 4D, respectively). Addition of exogenous C_16_-ceramide alone to naïve mitochondria resulted in formation of large MCRMs in 65–75% of mitochondria (third lanes and bottom lanes in Figures 4C and 4D), with recombinant full length Bax or BaxΔC co-localizing in ∼70% of these newly-formed platforms (identified as whitish-yellow merged signals in the bottom lanes). Further, more than half of the C_16_-ceramide uptake, 60±5% of the total, went to manufacturing these newly-formed MCRMs. MCRM formation was not an artifact of fixation preceding staining as reported for the Forssman antigen on the apical surface of MDCKII cells [58], as reversing this order did not impact MCRM detection (Figure S10).

We next isolated these MCRMs as a detergent-resistant light membrane fraction by continuous sucrose density flotation, a technique commonly employed to biophysically separate GEM platforms from bulk plasma membrane [18]. Figure 4E (middle panel, upper lane) confirms that Bax is absent from naïve mitochondria, and that upon incubation with BaxΔC, most of the BaxΔC (71%) resolves with the high-density bulk membrane-containing fraction (upper panel, upper lane, fractions 10–12). This compartment contains 57% of endogenous mitochondrial ceramide (upper panel, lower lane), and most of the MOM proteins VDAC (56%), Bak (89%), Bcl-xL (97%), and Metaxin (98%). Addition of 5 µM C_16_-ceramide, which alone does not effect cytochrome *c* release, doubled mitochondrial ceramide content from 936 to 1812 pmol/5 mg mitochondrial protein, largely within light sucrose density membranes (middle/lower panels, lower lanes, fractions 1–9), consistent with the confocal data showing that the majority of ceramide added goes to formation of MCRMs. In contrast to studies using BaxΔC alone, upon co-addition of BaxΔC and ceramide, a combination that releases most mitochondrial cytochrome *c* (see Figure 4A), 68% of BaxΔC resolves with the MCRMs (lower panel, upper lane, fractions 1–9). C_16_-dihydroceramide did not induce BaxΔC translocation into the light membrane fraction (Figure S10). A substantial portion of VDAC and a modest amount of Bak (lower panel, middle lanes), cholesterol (Figure S11) and various glycosphingolipids (not shown) also redistributed into this compartment upon C_16_-ceramide treatment, while Bcl-xL and several other membrane proteins remained within the high-density fraction (Figure 4E and Figure S11). Taken together, these data indicate that two distinct approaches, biophysical isolation and confocal microscopy, identify a ceramide-rich macrodomain where Bax and other MOMP-inducing proteins associate. How MCRMs bestow Bax pore-forming capability is not addressed in this study, although the data are consistent with MCRMs constituting an essential element of the proteolipid Bax pore that appears to regulate MOMP [7], [9].

### Ionizing radiation generates an endogenous MCRM in HeLa mitochondrial membranes

A similar approach was used to demonstrate MCRMs in irradiated HeLa mitochondria, which engage endogenous full-length Bax to mediate MOMP. While mitochondria isolated from unirradiated HeLa cells are decorated with attached but uninserted endogenous Bax (see Figure 3B, Figure S4), only 17±4% of these mitochondria manifest ceramide-rich domains, which tend to be small and contain minimal Bax by confocal microscopy (Figure 5A, upper lanes, n = 4). However, at 33 h post-10 Gy, MCRMs were detected in 41±4% of the total mitochondrial population (p<0.001). Up to 80% of the ceramide synthesized *de novo* (in 5 experiments 66±13% mean±SD) upon irradiation was utilized to construct these MCRMs. Hence MCRM ceramide rather than free ceramide would appear to be responsible for functionalizing Bax. Furthermore, the localization of Bax in MCRMs increased 14-fold after irradiation compared to that in the small ceramide-containing domains of control mitochondria (p<0.001), with 50% of the MCRMs displaying a Bax co-signal. Addition of 1 µM C_16_-ceramide to generate MCRMs in mitochondria isolated from control unirradiated HeLa cells, which maximally releases cytochrome *c* (see Figure 3A), mimicked ionizing radiation conferring Bax co-localization (typical image depicted in Figure 5B) in 56% of HeLa MCRMs (p<0.001 vs. control 0 µM ceramide).

Consistent with these confocal observations, biophysical isolation of ceramide-rich domains by either discontinuous (identified as the 5% sucrose light membrane fraction; Figure 5C) or continuous (Figure S12) sucrose density flotation 34 h post-10 Gy revealed that 80.4±12.0% of the radiation-induced mitochondrial ceramide elevation reported in Figure 1E was confined to the HeLa light membrane fraction post-radiation (not shown). These data are consistent with results where exogenous C_16_-ceramide added to isolated mouse liver mitochondria largely localized to this fraction (Figure 4E). Cholesterol was similarly concentrated in ceramide-rich light membranes post-radiation (Figure 5C). While a portion of Bak (20.2±1.1%) and VDAC (16.7±1.6%) constitutively associated with HeLa ceramide-rich light membranes, all other mitochondrial proteins tested, including Bcl-xL, were largely absent (Figure 5C and Figure S12). Little of the Bax (∼5–6%) associated with mitochondria in unirradiated HeLa cells resolved with ceramide-poor light membranes. However, after irradiation a substantial portion of the Bax pool (46%) localized to ceramide-rich light membranes. In eight experiments, we observed a 6.7±0.5 fold increase in light membrane-associated Bax (Figure 5C, lower panel; p<0.001). Thus two separate but complementary approaches, sucrose density floatation and confocal microscopy, confirm radiation-induced generation of mitochondrial MCRMs that coordinates Bax insertion/oligomerization required for MOMP.

### HeLa cell MCRMs contain high molecular weight oligomerized endogenous Bax

Because Bax oligomerization is an essential element of MOMP induction, the multimerization status of endogenous Bax within MCRMs was assessed post-irradiation. To assure that Bax multimerization within MCRMs was not an artifact of membrane lipid isolation, MCRMs were initially isolated either using 1–2% CHAPS, which does not impact low molecular weight homodimerization [37], [59], or 0.15% Triton X-100, known to induce low molecular weight homodimerization [37], [59]. After MCRMs were isolated, the molecular weight profile of associated Bax was defined by size exclusion chromatography. Isolation in CHAPS (Figure 5D, upper panels) revealed that MCRMs contained high molecular weight Bax oligomers exclusively, while the heavy fraction contained Bax monomers. A similar profile was identified using 0.15% Triton X-100 (Figure 5D, lower panels), except the molecular weight of Bax was slightly higher in these heavy fractions consistent with Bax-Bax homotypic interaction, as reported when using Triton X-100 [37]. VDAC and Bak were also found in MCRM high molecular weight oligomers, and in both high and low molecular weight fractions of heavy membranes. These studies define the MCRM as a site of preferential Bax oligomerization.

## Discussion

The present studies show that ceramide, formed upon ionizing radiation exposure, regulates Bax insertion, oligomerization and MOMP. As up to 80% of the radiation-generated ceramide in HeLa mitochondria resides in newly-formed MCRMs, and as a burgeoning literature defines plasma membrane CRMs as sites of protein sorting and oligomerization for transmembrane signaling (for review see [27]), the most parsimonious explanation for the current data set is that MCRMs, and not the small amount of free ceramide, represent the structure that functionalizes Bax. Consistent with this concept, all of the radiation-generated ceramide is found in a different physical state than the ceramide in resting HeLa cells. Whereas ceramide in resting cells is not found in macrodomains by confocal microscopy and associates with the heavy fraction by sucrose density flotation, the ceramide in irradiated cells is detected by confocal microscopy as MCRMs and by biophysical isolation in light membranes. This is identical to the pattern of resolution of plasma membrane CRMs by these same technologies.

An extensive literature exists regarding the capacity of ceramide to spontaneously form macrodomains in model membrane systems [57]. This is a rare property of lipids, resulting primarily from the capacity of the sphingoid base backbone of ceramide to network hydrogen bonds, and also via Van der Waal and hydrophobic forces [60]. Similar properties are detected upon addition of long chain ceramide to the exoplasmic surface of most mammalian cells or upon treatment of these cells with exogenous sphingomyelinase [61]. The current hypothesis in this field is that each membrane has a threshold level of ceramide that will yield a platform once achieved. Published experimental data support this suggestion [62]. Additionally, at least in model membranes, platform formation in an intrinsic property of ceramide, not requiring protein [57]. Our previous studies on plasma membrane responses during UV and Fas stress are consistent with the notion that ceramide primarily functions as membrane structure re-organizer. Those experiments showed that while ceramide elevation conferred plasma membrane platform formation, this event alone did not mimic stress signaling, as protein insertion and oligomerization were required for transmembrane signaling [29]. Here we extend these concepts to the MOM, showing that ceramide addition to mouse liver mitochondria confers an MCRM but not cytochrome *c* release (Figure 4), which additionally requires Bax function. These data differ from those of Colombini and co-workers, who provided evidence that ceramide alone at physiologic concentrations was capable of inducing MOMP [15], [63]. While we do not have an explanation for the difference between the current study and those of Colombini and co-workers, like ourselves, Kronke and co-workers [17] and Farber and co-workers [16] observed that ceramide synergized with Bax to induce MOMP, but did not act alone. Perhaps the use of different methods of delivery could be the source of the observed differences in the results.

The current studies provide a structural explanation for an emerging database on the role of ceramide in MOMP. Prior studies suggested that C_16_-ceramide, generated in the mitochondrial associated membrane [51] via Cers 5 and Cers 6 [47], traffics to the MOM where it synergizes with Bax in regulating cytochrome *c* release ([15] and current study). This is conceptually similar to the role of *de novo* synthesized ceramide at the outer mitochondrial membrane in radiation-induced apoptosis in the *C. elegans* germline [1], consistent with an evolutionary conservation of function. The advance of the current study is that a mitochondrial compartment that forms upon stress can be visualized and isolated (see similar data in [64]), and is pharmacologically-tractable, providing new opportunities for therapeutic manipulation of the death program.

Comparison of the ED_50_ for ceramide-mediated Bax insertion and for cytochrome *c* release indicates MCRMs improve Bax function as transducer of MOMP beyond enhancing Bax insertion. Whether this results from preferential Bax oligomerization within this specialized compartment, or whether MCRMs additionally facilitate Bax conformation as a “pore”, is currently unknown. Although the present studies do not attempt to distinguish the relative contribution of MCRM-mediated Bax insertion versus oligomerization in MOMP induction, it should nonetheless be emphasized that Bax oligomerization is fundamental to the process. Furthermore, although the constituents of a functional MCRM capable of yielding an active Bax pore remain largely unknown, we suggest that there is a core set of lipids and proteins that form the basic MCRM structure, which may be modified in a cell type and stress type manner. Thus it is anticipated that different stresses that utilize different pro-apoptotic Bcl-2 family members to initiate MOMP will accordingly generate MCRMs with distinct profiles.

It is also possible that inserted Bax may contribute to further MCRM organization, enabling recruitment or modification of specific proteins or lipids to form the active pore. This possibility is supported by observations made on pore-forming colicins. The inserted anti-parallel hairpin of colicins, like that of Bax [5], appears too short to span the membrane bilayer requiring membrane alterations upon hairpin insertion to enable pore function [9]. These studies demonstrated a toroidal structure forms within the bilayer upon hairpin insertion [9], [65], where pore walls interact directly with specific membrane lipids, such as cardiolipin, to generate a non-bilayer inverted micellar-like structure, conferred by hexagonal phase transition. This membrane conversion purportedly allows bilayer “thinning” permitting shorter peptides to extend through the bilayer. Relevant to this notion, long-chain ceramide represents one of only few lipids that have the propensity to spontaneously “destabilize” membranes by conferring hexagonal phase transition [66], [67], [68]. While the present data do not address the question whether ceramide directly participates in Bax pore structure, we posit that the biophysical property of ceramide to “destabilize” membranes may be crucial for conversion of inserted Bax into an open pore, regulating MOMP. This hypothesis, and whether the toroidal structure confers oligomerization or vice versa, requires further study.

The present studies also indicate that in the HeLa cell system, radiation activates CS within MAM, with apparent transfer of synthesized ceramide to MOM, as CS elevation is found only in MAM whereas the mass of ceramide increase is in the contiguous MOM. Understanding these events mechanistically is beyond the scope of the present studies, although the present studies do add to a growing literature indicating a role for ceramide in mitochondrial membranes in some forms of apoptosis [11], [15], [16], [47], [64]. Further, while our data indicate ceramide engagement in organizing MCRMs, the data do not preclude ceramide also acting at an earlier “pre-pore” stage of Bax activation. Recent studies reported C_2_-ceramide stimulates Bax conformational changes via PP2A-mediated Ser184 dephosphorylation [69]. However, our use of BaxΔC in the MCRM deconstruction studies, which lacks Ser184, restricts our observations to confirming a direct effect of ceramide on MOM reorganization to facilitate Bax insertion and oligomerization. Profiling for known pro-apoptotic (Bax, Bak, Bim, Bid, PUMA, VDAC), anti-apoptotic (Bcl-xL, Hsp60), and non-apoptotic (COXII, Metaxin, PDH E1a) proteins, which exist in distinct mitochondrial compartments, revealed assembly of Bax, Bak, and VDAC in ceramide-activated HeLa and mouse liver mitochondria MCRMs is selective, as all other proteins tested were confined to heavy membranes. It should be noted that mouse liver mitochondria, which are isolated without attached Bax, display no VDAC or Bak in the light membrane fraction prior to ceramide addition. In contrast, HeLa cell mitochondria, which purify with Bax attached and a minor portion inserted into the MCRM, display VDAC and Bak constitutively within their light membranes pre-radiation. Differences in the pattern of cholesterol concentration within liver and HeLa cell MCRMs were also noted (compare Figure S11 to Figure 5C). While reasons for these differences are currently unknown, ultimately Bax, Bak and VDAC concentrate specifically within MCRMs, segregated from Bcl-xL in both systems. Although the present data does not address the functional implications of MCRM separation of Bax and Bak from Bcl-xL, nonetheless this finding may be of potential significance. Future studies are required to further profile proteins within the MCRM and how they are selected, and to determine how ceramide induces preferential concentration of cholesterol and select glycosphingolipids within this compartment.

Coupling of CS, Bax insertion/oligomerization and MOMP in the pathogenesis of mitochondrial apoptosis sheds new light on CS in cellular and tissue responses to stress. CS has recently emerged as a cell-type specific mediator of apoptosis for diverse stresses *in vitro* [32], [54], [70], [71], [72], and as transactivator of disease pathogenesis in experimental models of human disease, including gastric ulcer in rats [73], radiation-mucositis in hamsters [74], VEGF receptor inhibitor (SU5416)-induced emphysema in rodents [75], and the radiation-induced GI syndrome in *atm^−/−^* mice [76]. In these disease models, CS-mediated tissue damage was significantly attenuated by FB_1_, leading to prevention or recovery from the stress-induced experimental syndrome. The present demonstration of the CS/Bax/MOMP linkage may have substantive implications for understanding normal tissue physiology and disease pathogenesis, potentially providing unique opportunities for targeted therapies.

## Supporting Information

Figure S1 **Ionizing radiation induces Bax insertion and oligomerization.** (A) Time-dependent Bax insertion after 10 Gy. Mitochondria isolated from HeLa cells at the indicated times post-irradiation were resuspended in 0.1 M Na_2_CO_3_, pH 11.5 (1 µg/µl) and incubated on ice for 1 h. 20 µl aliquots were analyzed for total Bax (upper panel, bottom lane). The remaining 80 µl were centrifuged at 100,000×*g* at 4°C for 30 min to separate alkali-sensitive (supernatant) and alkali-resistant (pellet) mitochondrial proteins. The pellet, containing Bax inserted into the MOM, was resuspended in 80 µl SHE buffer with 2% CHAPS and 20 µl aliquots of each fraction were immunoblotted using anti-Bax (N-20) and anti-COXII antibodies (upper panels), and quantified using NIH Image software (lower panel). The Bax/COXII ratio in unirradiated control (Co) at 36 h was arbitrarily valued of 1.0. Data represent 6 independent studies. (B) Bax oligomerization induced by irradiation. 34 h post-10 Gy, mitochondrial proteins extracted with 2% CHAPS buffer were separated by size on a Sephacryl S-200 gel filtration column equilibrated with 1% CHAPS buffer at a flow rate of 0.5 ml/min. 400 µl aliquots of each 1 ml collected fraction were concentrated by 20% TCA precipitation, and immunoblotted using anti-Bax, anti-Bak and anti-VDAC antibodies. Data are from 1 of 4 independent studies.
(TIF)

Figure S2 **Validation of the specificity and sensitivity of the ceramide antibody MID 15B4 by immune TLC.** 1 µmol of C_16_-ceramide, C_16_-dihydroceramide, and sphingomyelin were separated on a silica gel 60 Å TLC plates using chloroform: acetone: methanol: acetic acid: water (10∶4∶3∶2∶1, v/v) as solvent. Plates were blocked overnight with 4% BSA at 4°C. After washing, plates were incubated with the anti-ceramide antibodies MID 15B4 (final concentration 6 µg/ml) at 4°C for 2 h followed by alkaline phosphatase-coupled anti-mouse IgM antibody for 2 h at room temperature. Plates were washed and lipids visualized with Tropix chemiluminescence kit. This figure is representative of 3 similar studies.
(TIF)

Figure S3 **Co-localization of anti-ceramide with anti-KDEL before and after 10 Gy in HeLa cells.** At 36 h post-irradiation, HeLa cells were stained with anti-ceramide IgM (green) and anti-KDEL (red). Images were obtained using a Zeiss LSM 510 confocal microscope fitted with a ×63 objective and co-localization analysis was performed with LSM 510 v.2.8 software. Pixel overlap between two scans (i.e., Texas-Red and Cy2) of the same specimen is presented as a scatter diagram. Two pixels (P1, a pixel from the scan of the red channel and P2, a pixel from the scan of the green channel) with the exact same position in both scans are considered a pair (P1, P2). In the scatter diagram, the brightness level of P1 is plotted on the X coordinate and that of P2 on the Y coordinate. Each dot on the scatter diagram represents the pixel pair. Relative frequency of occurrence of a particular pair of pixels is expressed with different colors (red>orange>yellow>green>blue>black) representing highest to lowest frequency. Thus, two completely identical scans would result in a straight diagonal line from bottom left to top right on the diagram, while the diagram will show scattered dots in case of insignificant co-localization. The specific level of co-localization was quantified after threshold intensities of the two fluorophores were adjusted by subtracting non cell-associated background pixels from the scatter diagram. In control cells (0 Gy) the scatter diagram of ceramide and KDEL staining was detected in the 45 degrees axial position, indicating significant overlap of ceramide and KDEL, while in the irradiated sample the scatter diagram is closer to horizontal plane, indicating reduced co-localization of ceramide and KDEL after irradiation. Data are from 1 of 4 experiments.
(TIF)

Figure S4 **Ceramide induces insertion of endogenous Bax into HeLa mitochondrial membranes.** Isolated mitochondria were incubated with 0–1 µM C_16_-ceramide and mitochondrial pellets were collected after incubation as in Figure 3A. Attached and inserted Bax were separated by alkali extraction of mitochondrial pellet as in Figure 2B and analyzed by Western blot with anti-Bax and anti-COXII as loading control.
(TIF)

Figure S5 **Inhibition of GST-BaxΔC induced cytochrome** ***c*** **release from mitochondria isolated from FB_1_-treated HeLa cells or BAEC.** Upper panel: Mitochondria were isolated from 50 µM FB_1_-pretreated or untreated HeLa cells and incubated in MSB buffer (1 µg mitochondrial protein/µl) with 4 µM GST-BaxΔC for 1 h at 30°C. After incubation, mitochondria were pelleted by centrifugation at 14,000×*g* for 5 min at 4°C, and supernatants analyzed for cytochrome *c* release by Western blotting. Lower panel: Mitochondria were isolated from 50 µM FB_1_-pretreated or untreated BAEC. After incubation with GST-BaxΔC (0–3 µM) in MSB buffer (1 µg mitochondrial protein/µl), mitochondria were pelleted, and the resulting supernatants analyzed for cytochrome *c* release. These data are from 1 representative of 3 independent investigations each.
(TIF)

Figure S6 **Purity of isolated mouse liver mitochondria.** Organelles were isolated from 8-week-old C57BL/6 mouse liver by sequential gravity centrifugation as described in Materials and Methods. All steps were performed at 4°C. Briefly, mouse liver homogenate in Buffer A (0.25 M sucrose, 10 mM HEPES pH 7.4, 0.5 mM EGTA) was centrifuged at 600×*g* for 15 min to remove nuclei, cell debris and unbroken cells. The post-nuclear supernate was then centrifuged at 10,000×*g* for 15 min to separate heavy membranes (P10) from the cytosol and light membrane fraction (S10). This supernatant was further centrifuged at 100,000×*g* for 1 h to separate cytosol (S100) from microsomes (P100). The heavy membrane fraction (P10) was washed 4 more times by centrifugation at 10,000×*g* for 15 min to remove microsomal contamination. 15 µg of each fraction were analyzed by Western blot using specific organelle markers; Na^+^/K^+^-ATPase (plasma membrane), Sec61 (ER), Flotillin-1 (plasma membrane), COX IV (mitochondria). Western blot with anti-Bax (N20) shows that isolated mouse liver mitochondria are free of Bax as reported previously [46].
(TIF)

Figure S7 **C_16_-ceramide enhances Bax-induced cytochrome** ***c*** **release without disrupting the inner mitochondrial membrane of isolated mouse liver mitochondria.** Mitochondria were isolated from mouse liver by differential centrifugation and treated with C_16_-ceramide and 50 nM BaxΔC as described in Materials and Methods. While cytochrome *c* was released by addition of C_16_-ceramide and 50 nM BaxΔC, the soluble mitochondrial matrix protein Hsp60 was retained in mitochondria.
(TIF)

Figure S8 **C_16_-ceramide and C_16_-dihydroceramide uptake by isolated mouse liver mitochondria and their effect on BaxΔC insertion and cytochrome** ***c*** **release.** Upper panel: C_16_-ceramide or C_16_-dihydroceramide was added to 5 mg mitochondria in 1×KCl buffer (1 mg mitochondrial prot./ml) and incubated for 5 min at 37°C. Mitochondrial pellets were collected by centrifugation for 10 min at 10,000×*g* at 4°C, and lipids were extracted and ceramide and dihydroceramide were measured using the diacylglycerol kinase assay as in Materials and Methods. Lower panel: 1 µM C_16_-ceramide or C_16_-dihydroceramide was incubated with or without 50 nM BaxΔC with mitochondria and BaxΔC insertion (left) and cytochrome *c* release (right) were analyzed as in Figure 4A and B.
(TIF)

Figure S9 **Profile of the proteins released from isolated mouse liver mitochondria by exogenous C_16_-ceramide and recombinant BaxΔC.** Mitochondria isolated from mouse liver were incubated with KCl buffer (1 mg mitochondrial protein/ml) without BSA for 5 min at 37°C with C_16_-ceramide and BaxΔC as indicated. After incubation, reaction samples were centrifuged at 14,000×*g* for 5 min at 4°C and the supernatants containing proteins released from mitochondria were collected and analyzed by Coomassie blue staining (15 µg/lane; upper panel) or by immunoblotting (15 µg/lane) with anti-cytochrome *c* (lower panel). The control (CO) profile in the absence of either BaxΔC or ceramide is very similar to that observed in isolated rat liver mitochondria [77]. 50 nM BaxΔC plus 5 µM C_16_-ceramide and 1 µM BaxΔC display a nearly-identical set of additionally released proteins, marked by asterisks (*), indicative of the same release mechanism. Further, no inner mitochondrial space proteins over 116 kD in size were released by 50 nM BaxΔC plus 5 µM C_16_-ceramide or 1 µM BaxΔC, as confirmed by mass spectrometry, consistent with published literature that defines the maximal size of proteins released through the Bax pore as 123 kD [78].
(TIF)

Figure S10 **The effect of fixation on the staining of MCRM using anti-ceramide IgM.** It has been reported by Butor et al. [58] that in some instances secondary antibodies induce clustering of lipids on cell surfaces, rectified if the fixation step is carried out after the first antibody-labeling step. To exclude this possibility, we compared the staining profile of MCRMs using anti-ceramide IgM with or without fixation using 2% formaldehyde. Mouse hepatic mitochondria were isolated and treated with 0 mM or 5 mM ceramide for 5 min at 37°C to induce MCRM formation as described in Materials and Methods. After incubation with blocking solution (3% FBS/3% goat serum) containing 200 nM Mitotracker for 60 min on ice, one set of samples was stained directly with anti-ceramide IgM, followed by fixation with 2% formaldehyde and the other was fixed with 2% formaldehyde, then stained with anti-ceramide. Mitochondria were subsequently incubated with secondary anti-IgM antibody and mounted on slides. Images, acquired with a Leica TCS AOBS SP2 confocal microscope equipped with a 100×1.4NA OIL DIC D objective combined with 2× scan zoom, were analyzed with MetaMorph 7.5 software.
(TIF)

Figure S11 **Distribution of mouse liver mitochondrial proteins in 5–30% continuous sucrose density gradient.** After incubation for 5 min at 37°C with 50 nM BaxΔC with or without 5 µM C_16_-ceramide, mitochondria were pelleted by centrifugation at 10,000×*g* for 10 min at 4°C and resuspended in cold MBS buffer containing 0.05% Triton X-100. After 30 min incubation on ice, mitochondria were homogenized with 20 strokes of a loose-fitting dounce homogenizer. The mitochondrial homogenate was adjusted to 40% final sucrose concentration and subjected to 5–30% continuous sucrose density gradient centrifugation as described in Materials and Methods. 400 µl of each 1 ml fraction was used for immunoblot analysis using the indicated antibodies as in Figure 4E. A set of pro-apoptotic proteins including (Bax, Bak, Bim, Bid, PUMA, VDAC), anti-apoptotic proteins (Bcl-xL, Hsp60), and non-apoptotic proteins (COXII, Metaxin, PDH E1α) that exist in distinct compartments were examined. While Bax, Bak, Bim, Bid, PUMA, VDAC, Metaxin and Bcl-xL are outer membrane proteins, COXII is an inner membrane protein and Hsp60 and PDH E1α are matrix proteins.
(TIF)

Figure S12 **Ionizing radiation (10 Gy) induces specific translocation of Bax into HeLa cell MCRMs.** Mitochondria were isolated from HeLa cells 34 h after irradiation and incubated with 0.15% Triton X-100 in MBS buffer for 30 min on ice. The mitochondrial suspension was homogenized with 20 strokes of a loose-fitting dounce homogenizer, adjusted to 40% final sucrose concentration and subjected to 5–30% continuous sucrose density gradient centrifugation as described in Experimental Procedures. 400 µl aliquots from each fraction were analyzed by Western blotting using the indicated antibodies after 20% TCA precipitation.
(TIF)

Text S1 (DOC)
