# Correction: Mitochondrial Ceramide-Rich Macrodomains Functionalize Bax upon Irradiation

**DOI:** 10.1371/journal.pone.0146210

**Published:** 2015-12-30

**Authors:** Hyunmi Lee, Jimmy A. Rotolo, Judith Mesicek, Tuula Penate-Medina, Andreas Rimner, Wen-Chieh Liao, Xianglei Yin, Govind Ragupathi, Desiree Ehleiter, Erich Gulbins, Dayong Zhai, John C. Reed, Adriana Haimovitz-Friedman, Zvi Fuks, Richard Kolesnick

The authors would like to correct Fig 5, as errors were introduced in the preparation of this figure for publication. In Fig 5D[b], the image for the Light fraction Bak Western blot and the numbering of the fractions are incorrect. The authors have provided a corrected version of Fig 5 here.

The authors confirm that these changes do not alter their findings. The authors have provided the original Western blot and additional underlying data as Supporting Information.

**Fig 5:**
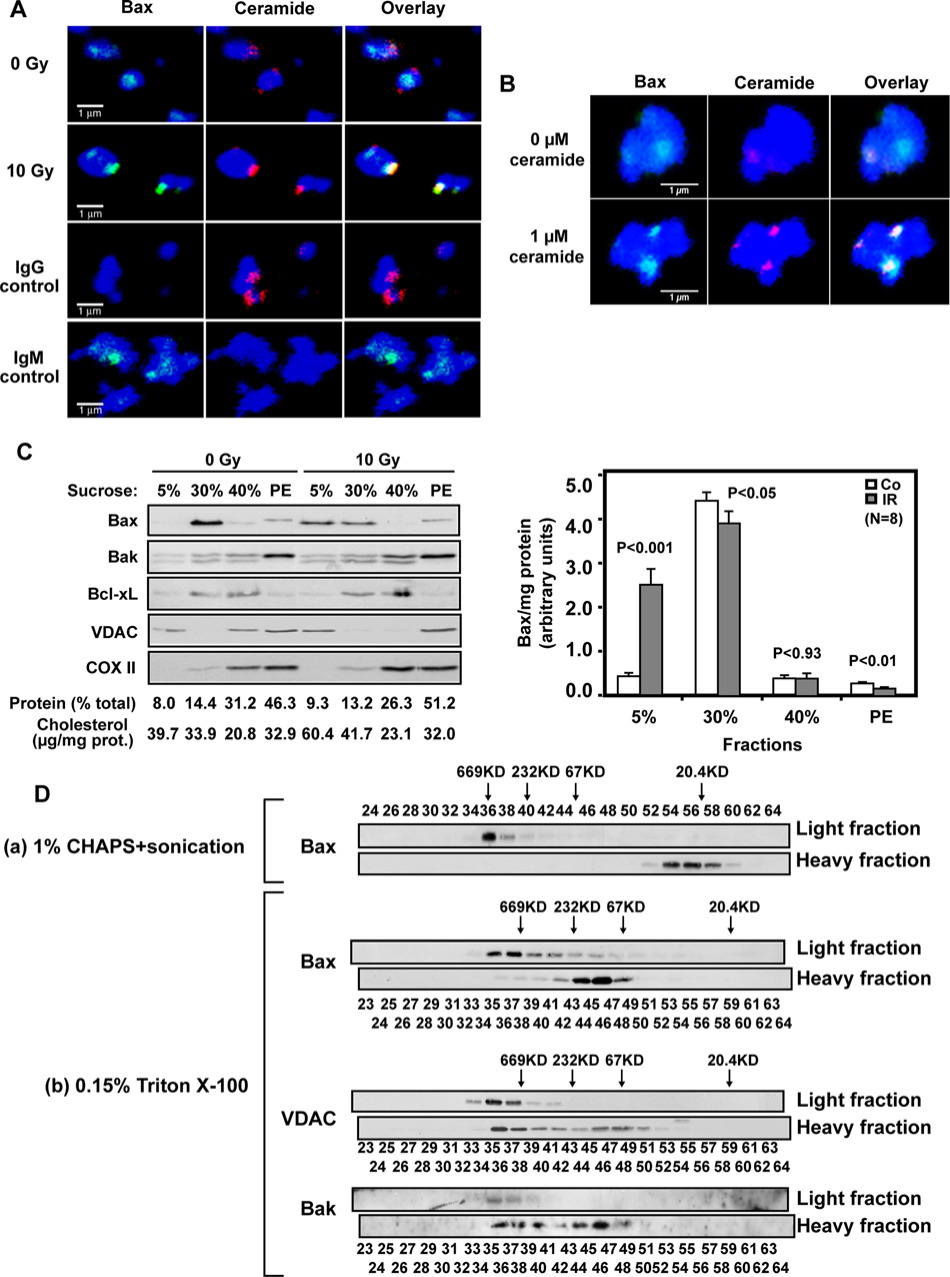
Ceramide induces formation of a mitochondrial ceramide-rich macrodomain (MCRM). (A) Ionizing radiation (10 Gy) induces co-localization of endogenous Bax with MCRMs in HeLa cells. Mitochondria were isolated from HeLa cells 34 h after irradiation and immunostained as described in Supporting Information Text S1. Data represent typical stainings from 1 of 4 similar studies in which 2000 mitochondria were analyzed each. (B) Addition of exogenous C16-ceramide induces co-localization of endogenous full-length Bax with MCRMs in HeLa cells. Mitochondria were isolated from HeLa cells using percoll gradient and treated with ceramide as Figure 3A. After 30 min incubation, mitochondria were fixed and stained with MitoTracker (blue), while ceramide and Bax were localized using anti-ceramide IgM (red) or anti-Bax IgG (green), respectively. Control IgM and IgG did not yield detectable signals (not shown). These data represent 1 of 3 similar studies. (C) Bax translocates into a radiation-generated HeLa MCRM. Upper panel: 34 h post-irradiation, HeLa mitochondria were isolated as in Materials and Methods and incubated with 0.15% Triton X-100 in MBS buffer for 30 min on ice. 40 μl mitochondrial homogenate (3.3 mg/μl) were subjected to 5–30% mini-discontinuous sucrose density gradient centrifugation as described in Materials and Methods. 20 μl aliquots of 80 μl fractions were analyzed by immunoblotting using the indicated antibodies. The protein level of each fraction was assessed using the Bio-Rad Dc protein assay kit (PE, Pellet). Data are from 1 of 4 studies, consisting of 2 independent gradients per study. The gradient shown displays our clearest example of Bax translocation into light membranes. Lower panel: Bax in each fraction, revealed by immunoblotting and quantified using NIH Image software, was normalized to protein content for all 8 gradients. (D) MCRM Bax exists as high molecular weight oligomers. Mitochondria from 10 Gy-irradiated HeLa cells, disrupted by either (a) 1% CHAPS and sonication or (b) dounce homogenization in 0.15% Triton X-100, were subjected to 5–30% discontinuous sucrose gradient for MCRM isolation as in Experimental Procedures. Proteins associated with Light and Heavy Fractions were analyzed by gel filtration on a Sephacryl S-200 column as in Figure 2C. 500 μl of each eluted fraction from Sephacryl S-200 were concentrated by 20% TCA precipitation for immunoblotting. For panel (b), as Light Fraction Bax, VDAC and Bak were resolved from odd-numbered fractions recovered from the Sephacryl S-200 gel filtration column while Heavy Fraction proteins were from even-numbered fractions, lanes have been renumbered and realigned. Heavy and Light Fraction lanes are now offset by one fraction. Furthermore, the Bak Light Fraction lane has been replaced with a version that more accurately reflects the original blot. These revisions do not alter the scientific message of the figure, which is that Bax integrates into a MCRM after radiation that contains VDAC and Bak constitutively. Data are from 3 independent studies.

## Supporting Information

S1 Fig Original Bak Western Blot. This blot, dated 11-30-05, is the original used to prepare Bak protein bands for Fig 5D(b). To analyze oligomerization status of Bax and other mitochondrial proteins such as VDAC and Bak in Mitochondrial Ceramide Rich Macrodomains (MCRMs), mitochondria were isolated from either control (Co) unirradiated HeLa cells or HeLa cells at 34 hours or 36 hours after 10 Gy. Proteins in isolated mitochondria were solubilized in 0.15% Triton X-100 or 1% CHAPS, and subjected to Dounce homogenization or sonication, respectively. Solubilized mitochondrial fractions were adjusted to 40% sucrose, placed in the bottom of a centrifugation tube, and overlaid with a 5–30% discontinuous sucrose gradient. Detergent-insoluble Light Membrane Fractions migrated to the 5%-30% sucrose interface whereas Heavy Fractions were retained in 40% sucrose after overnight centrifugation. Proteins associated with Light and Heavy Fractions were thereafter separated by molecular weight using a Sephacryl S-200 size-exclusion gel filtration chromatography column pre-calibrated with molecular weight markers in the range of 20.4 kDa to 669 KDa. One ml-sized fractions eluted from the gel filtration column were collected, and 500 μl aliquots of every other fraction between 21–65 were concentrated by TCA precipitation. Due to the large number of fractions, two 12–15% discontinuous SDS-PAGE gels were required to resolve proteins eluted in each fraction. For each elution, Gel 1 contained odd-numbered fractions 21–47 and Gel 2 contained odd-numbered fractions 49–65. To minimize variability, proteins, separated in the six gels (2 each for Control, and 34 hours and 36 hours post irradiation), were transferred to a single sheet of PVDF membrane and immunoblotted with anti-Bak antibody. Note that lanes containing molecular weight markers on the sides and the junctions of the two gels (white arrows) display immunoblot signal artifact.
(TIF)

S2 Fig Assembly of panels displaying Bak bands from the original blot. Panels displaying Bak bands isolated by gel filtration as in Figure 1 were generated from lane fractions 23–63 of the original blot and assembled in order of time of radiation. Note deletion of the molecular weight marker lanes and gel junctions for preparation of data for publication. These data show that Bak exists in HeLa mitochondrial Light Membrane Fractions before and after irradiation in a high molecular weight complex. * indicates the region where the two gels were originally apposed.
(TIF)

S3 Fig Corrected Fig 5D(b)—MCRM Bak exists as a high molecular weight oligomer. As Light Fraction Bax, VDAC and Bak from the 34 hour post radiation time point were resolved from odd-numbered fractions recovered from the Sephacryl S-200 gel filtration column while Heavy Fraction proteins were from even-numbered fractions, lanes have been renumbered and realigned. Heavy and Light Fraction lanes are now offset by one fraction. Furthermore, the Bak Light Fraction lane has been replaced with a version that more accurately reflects the Original Blot. These revisions do not alter the scientific message of the figure, which is that Bax integrates into a MCRM after radiation that contains VDAC and Bak constitutively.
(TIF)
