# Supplementary figures and images for: Mitochondrial Ceramide-Rich Macrodomains Functionalize Bax upon Irradiation
Source: PLoS One. 2011 Jun 13;6(6):e19783. doi: 10.1371/journal.pone.0019783 (PMC3113798; doi:10.1371/journal.pone.0019783)

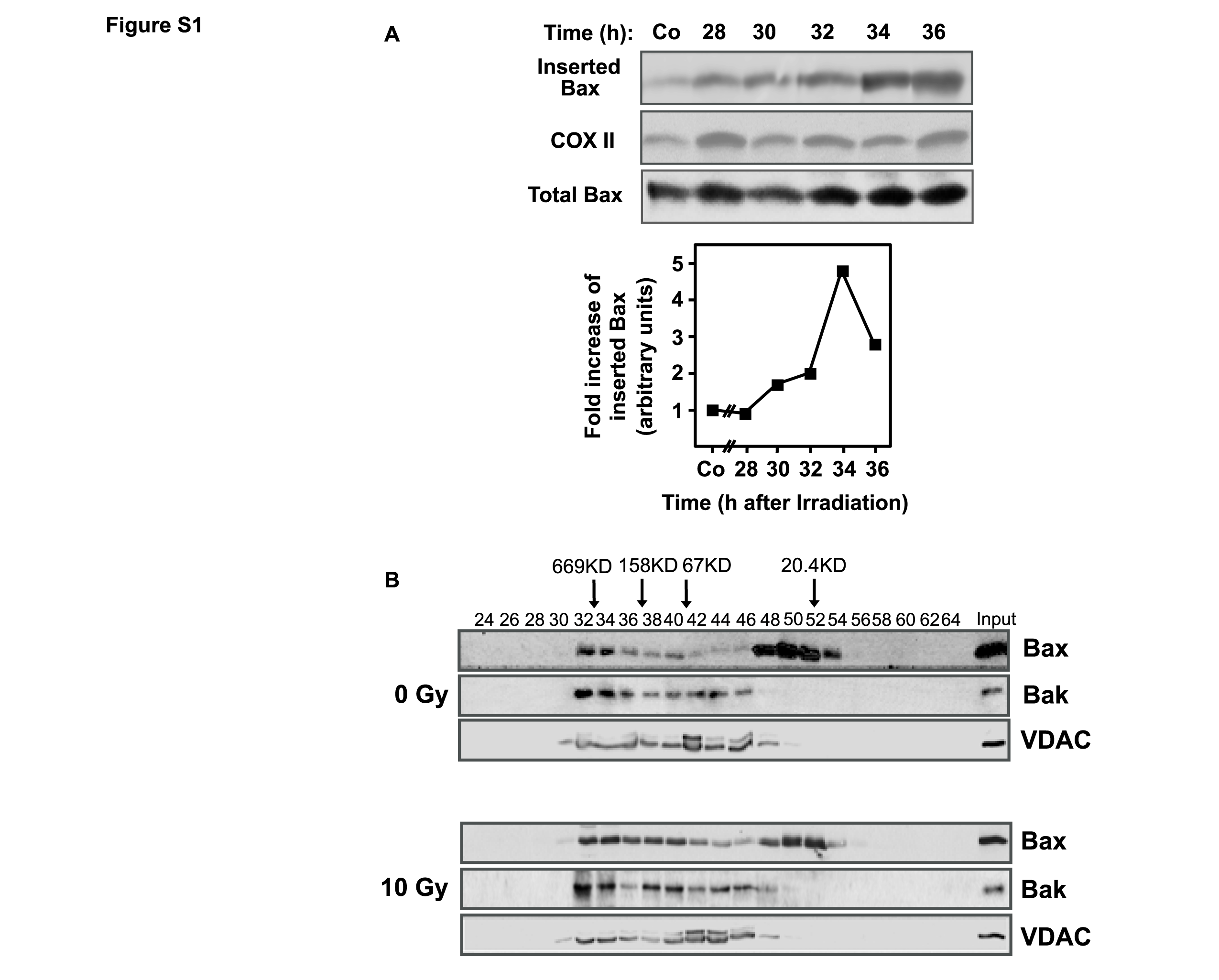

Supplement: Figure S1 — Ionizing radiation induces Bax insertion and oligomerization. (A) Time-dependent Bax insertion after 10 Gy. Mitochondria isolated from HeLa cells at the indicated times post-irradiation were resuspended in 0.1 M Na2CO3, pH 11.5 (1 µg/µl) and incubated on ice for 1 h. 20 µl aliquots were analyzed for total Bax (upper panel, bottom lane). The remaining 80 µl were centrifuged at 100,000×g at 4°C for 30 min to separate alkali-sensitive (supernatant) and alkali-resistant (pellet) mitochondrial proteins. The pellet, containing Bax inserted into the MOM, was resuspended in 80 µl SHE buffer with 2% CHAPS and 20 µl aliquots of each fraction were immunoblotted using anti-Bax (N-20) and anti-COXII antibodies (upper panels), and quantified using NIH Image software (lower panel). The Bax/COXII ratio in unirradiated control (Co) at 36 h was arbitrarily valued of 1.0. Data represent 6 independent studies. (B) Bax oligomerization induced by irradiation. 34 h post-10 Gy, mitochondrial proteins extracted with 2% CHAPS buffer were separated by size on a Sephacryl S-200 gel filtration column equilibrated with 1% CHAPS buffer at a flow rate of 0.5 ml/min. 400 µl aliquots of each 1 ml collected fraction were concentrated by 20% TCA precipitation, and immunoblotted using anti-Bax, anti-Bak and anti-VDAC antibodies. Data are from 1 of 4 independent studies. (TIF) [file pone.0019783.s001.tif]

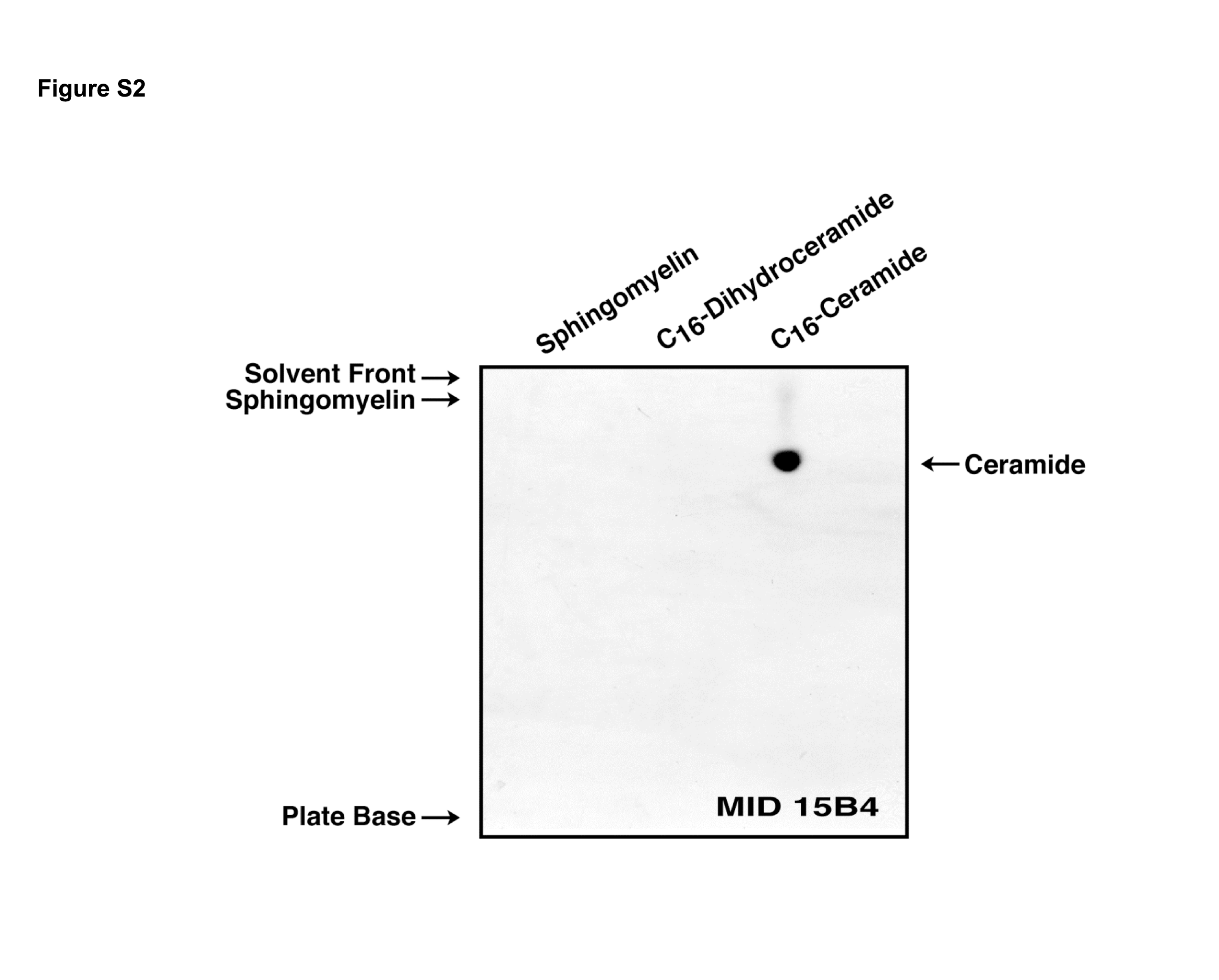

Supplement: Figure S2 — Validation of the specificity and sensitivity of the ceramide antibody MID 15B4 by immune TLC. 1 µmol of C16-ceramide, C16-dihydroceramide, and sphingomyelin were separated on a silica gel 60 Å TLC plates using chloroform: acetone: methanol: acetic acid: water (10∶4∶3∶2∶1, v/v) as solvent. Plates were blocked overnight with 4% BSA at 4°C. After washing, plates were incubated with the anti-ceramide antibodies MID 15B4 (final concentration 6 µg/ml) at 4°C for 2 h followed by alkaline phosphatase-coupled anti-mouse IgM antibody for 2 h at room temperature. Plates were washed and lipids visualized with Tropix chemiluminescence kit. This figure is representative of 3 similar studies. (TIF) [file pone.0019783.s002.tif]

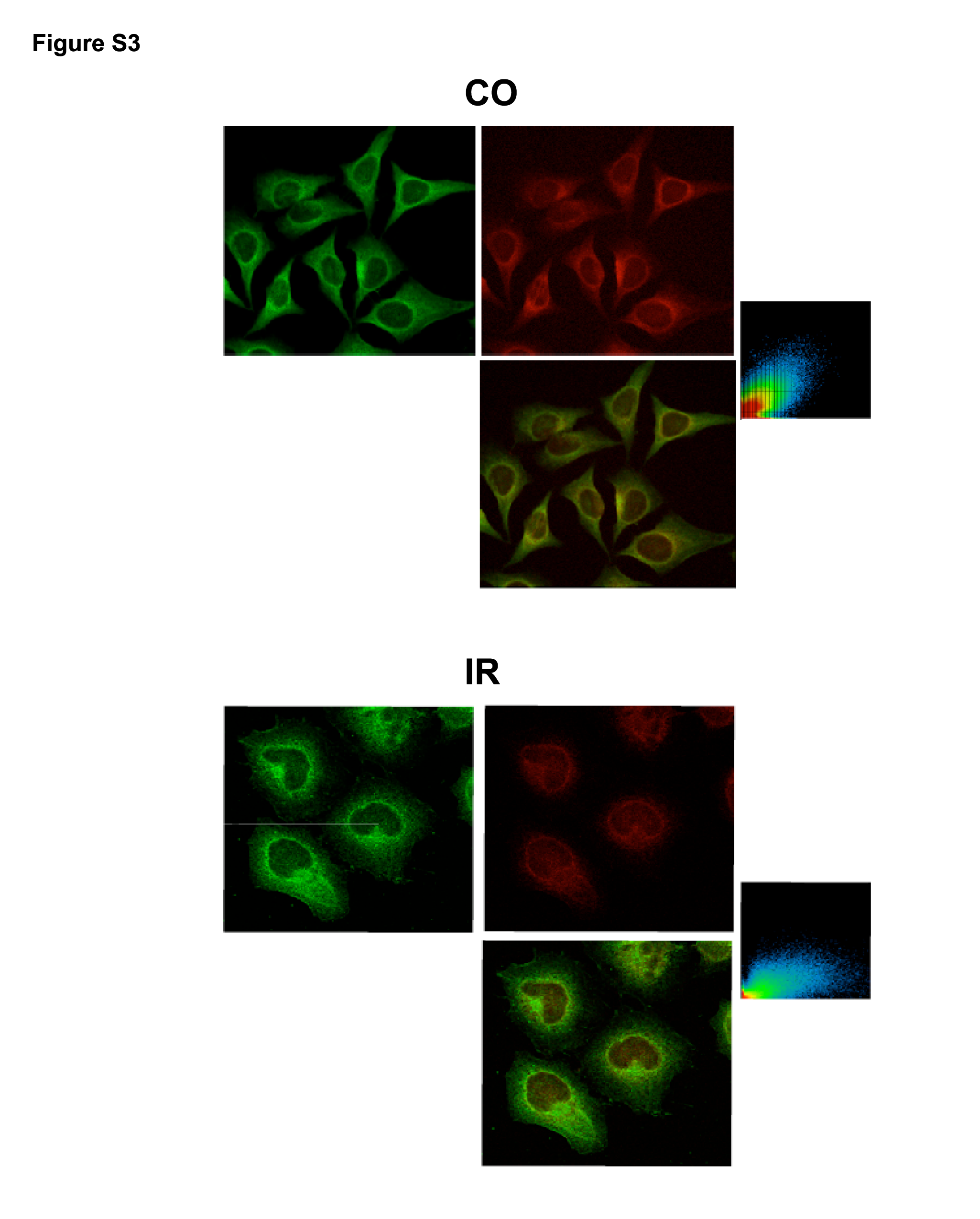

Supplement: Figure S3 — Co-localization of anti-ceramide with anti-KDEL before and after 10 Gy in HeLa cells. At 36 h post-irradiation, HeLa cells were stained with anti-ceramide IgM (green) and anti-KDEL (red). Images were obtained using a Zeiss LSM 510 confocal microscope fitted with a ×63 objective and co-localization analysis was performed with LSM 510 v.2.8 software. Pixel overlap between two scans (i.e., Texas-Red and Cy2) of the same specimen is presented as a scatter diagram. Two pixels (P1, a pixel from the scan of the red channel and P2, a pixel from the scan of the green channel) with the exact same position in both scans are considered a pair (P1, P2). In the scatter diagram, the brightness level of P1 is plotted on the X coordinate and that of P2 on the Y coordinate. Each dot on the scatter diagram represents the pixel pair. Relative frequency of occurrence of a particular pair of pixels is expressed with different colors (red>orange>yellow>green>blue>black) representing highest to lowest frequency. Thus, two completely identical scans would result in a straight diagonal line from bottom left to top right on the diagram, while the diagram will show scattered dots in case of insignificant co-localization. The specific level of co-localization was quantified after threshold intensities of the two fluorophores were adjusted by subtracting non cell-associated background pixels from the scatter diagram. In control cells (0 Gy) the scatter diagram of ceramide and KDEL staining was detected in the 45 degrees axial position, indicating significant overlap of ceramide and KDEL, while in the irradiated sample the scatter diagram is closer to horizontal plane, indicating reduced co-localization of ceramide and KDEL after irradiation. Data are from 1 of 4 experiments. (TIF) [file pone.0019783.s003.tif]

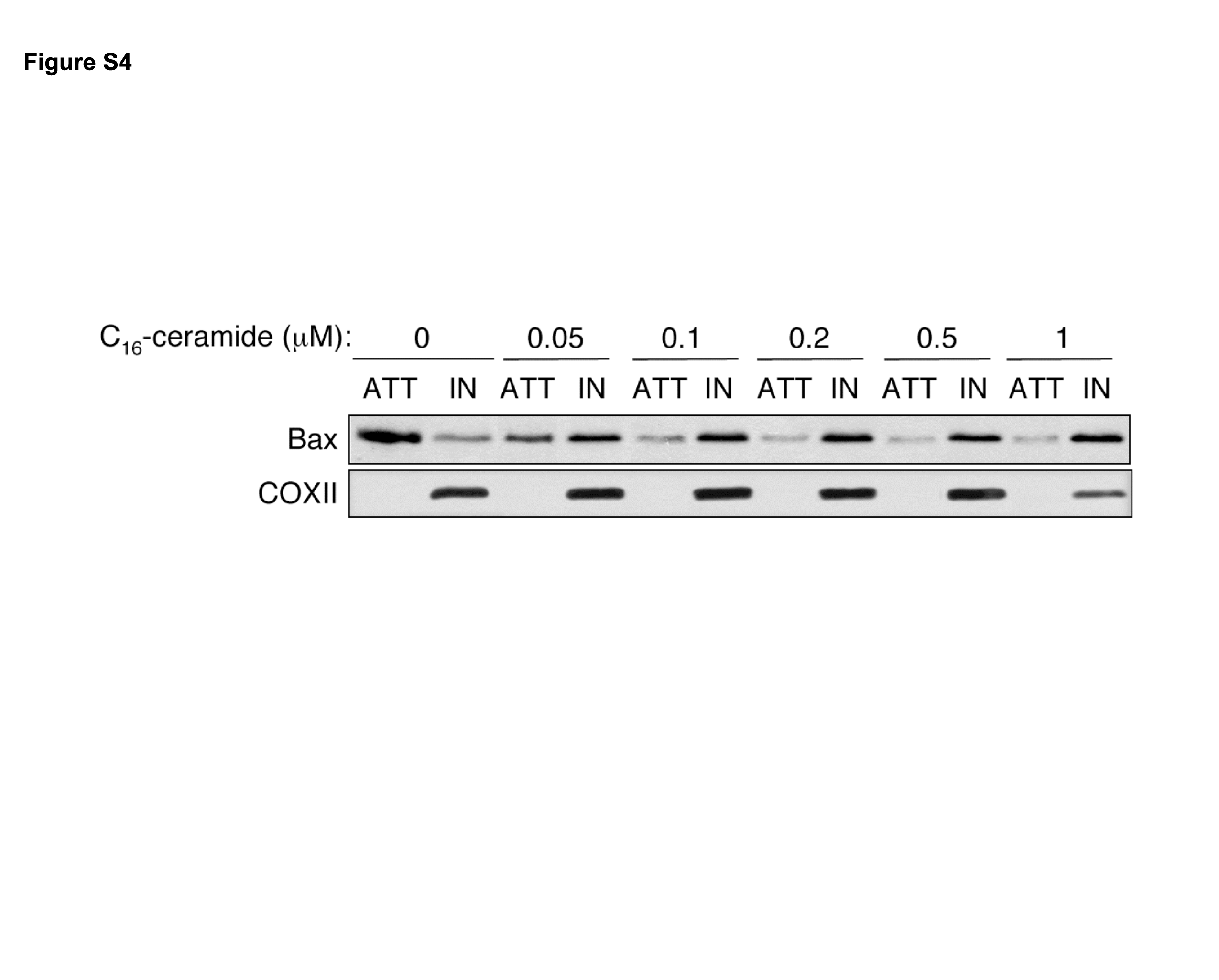

Supplement: Figure S4 — Ceramide induces insertion of endogenous Bax into HeLa mitochondrial membranes. Isolated mitochondria were incubated with 0–1 µM C16-ceramide and mitochondrial pellets were collected after incubation as in Figure 3A. Attached and inserted Bax were separated by alkali extraction of mitochondrial pellet as in Figure 2B and analyzed by Western blot with anti-Bax and anti-COXII as loading control. (TIF) [file pone.0019783.s004.tif]

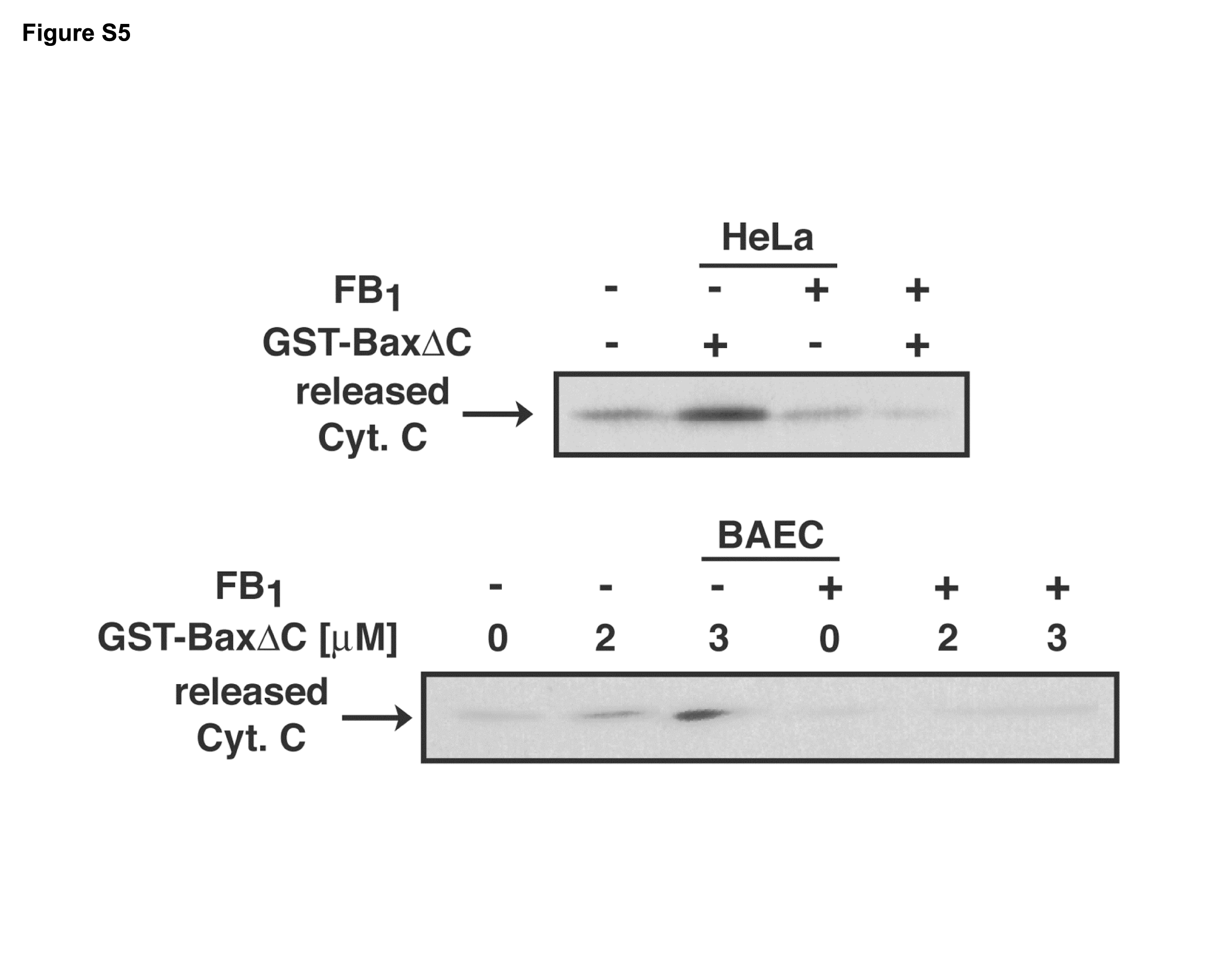

Supplement: Figure S5 — Inhibition of GST-BaxΔC induced cytochrome c release from mitochondria isolated from FB1-treated HeLa cells or BAEC. Upper panel: Mitochondria were isolated from 50 µM FB1-pretreated or untreated HeLa cells and incubated in MSB buffer (1 µg mitochondrial protein/µl) with 4 µM GST-BaxΔC for 1 h at 30°C. After incubation, mitochondria were pelleted by centrifugation at 14,000×g for 5 min at 4°C, and supernatants analyzed for cytochrome c release by Western blotting. Lower panel: Mitochondria were isolated from 50 µM FB1-pretreated or untreated BAEC. After incubation with GST-BaxΔC (0–3 µM) in MSB buffer (1 µg mitochondrial protein/µl), mitochondria were pelleted, and the resulting supernatants analyzed for cytochrome c release. These data are from 1 representative of 3 independent investigations each. (TIF) [file pone.0019783.s005.tif]

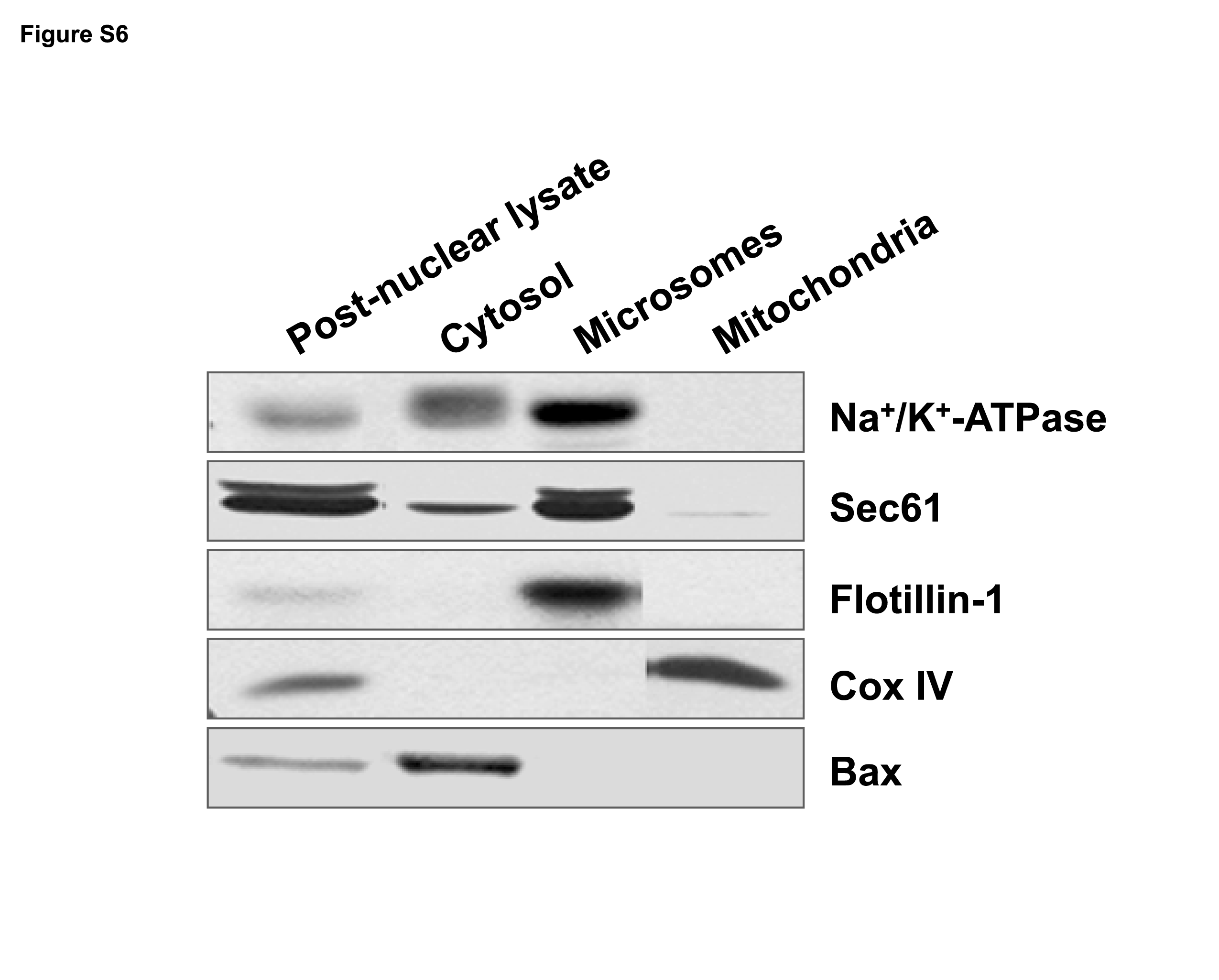

Supplement: Figure S6 — Purity of isolated mouse liver mitochondria. Organelles were isolated from 8-week-old C57BL/6 mouse liver by sequential gravity centrifugation as described in Materials and Methods. All steps were performed at 4°C. Briefly, mouse liver homogenate in Buffer A (0.25 M sucrose, 10 mM HEPES pH 7.4, 0.5 mM EGTA) was centrifuged at 600×g for 15 min to remove nuclei, cell debris and unbroken cells. The post-nuclear supernate was then centrifuged at 10,000×g for 15 min to separate heavy membranes (P10) from the cytosol and light membrane fraction (S10). This supernatant was further centrifuged at 100,000×g for 1 h to separate cytosol (S100) from microsomes (P100). The heavy membrane fraction (P10) was washed 4 more times by centrifugation at 10,000×g for 15 min to remove microsomal contamination. 15 µg of each fraction were analyzed by Western blot using specific organelle markers; Na+/K+-ATPase (plasma membrane), Sec61 (ER), Flotillin-1 (plasma membrane), COX IV (mitochondria). Western blot with anti-Bax (N20) shows that isolated mouse liver mitochondria are free of Bax as reported previously [46]. (TIF) [file pone.0019783.s006.tif]

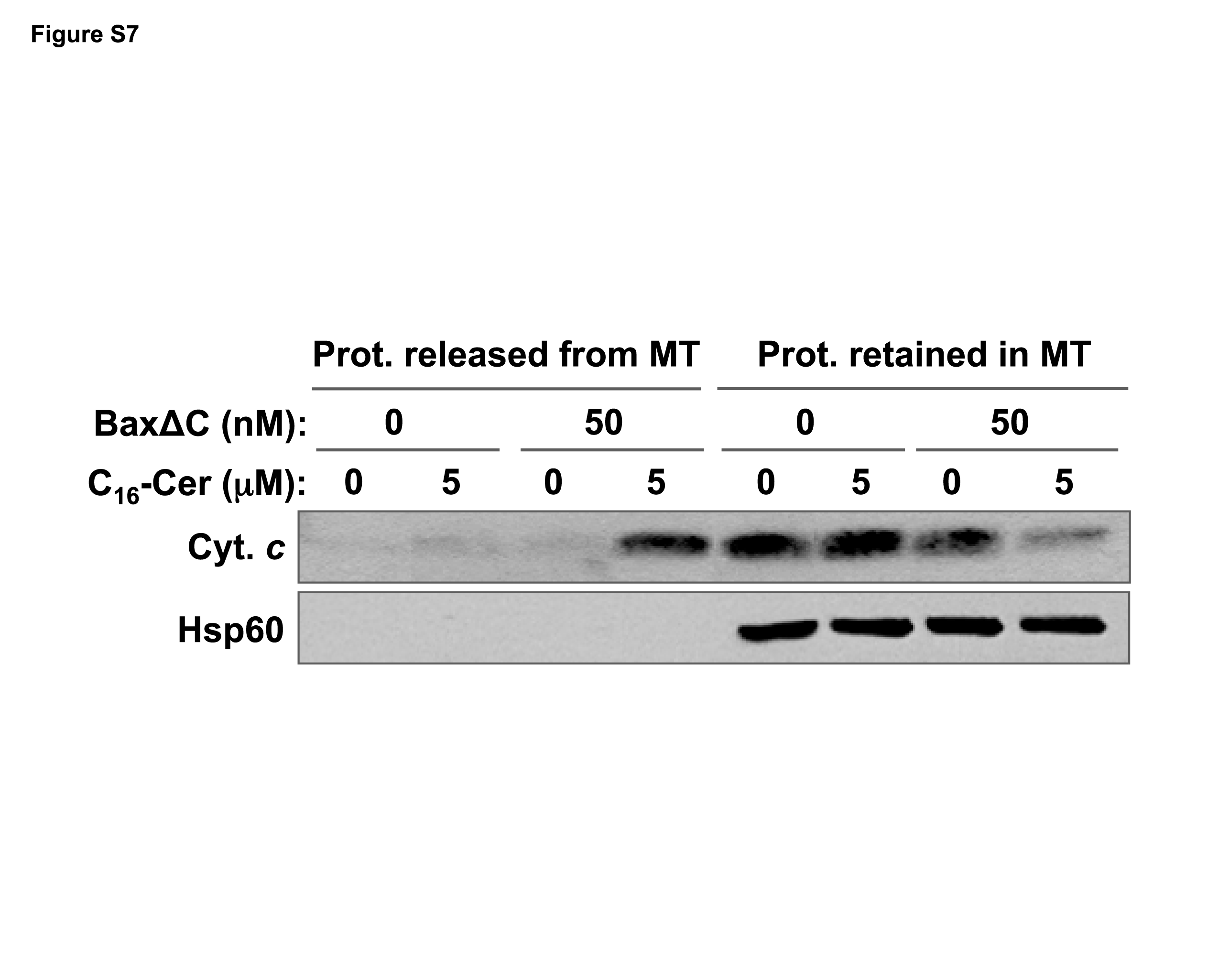

Supplement: Figure S7 — C16-ceramide enhances Bax-induced cytochrome c release without disrupting the inner mitochondrial membrane of isolated mouse liver mitochondria. Mitochondria were isolated from mouse liver by differential centrifugation and treated with C16-ceramide and 50 nM BaxΔC as described in Materials and Methods. While cytochrome c was released by addition of C16-ceramide and 50 nM BaxΔC, the soluble mitochondrial matrix protein Hsp60 was retained in mitochondria. (TIF) [file pone.0019783.s007.tif]

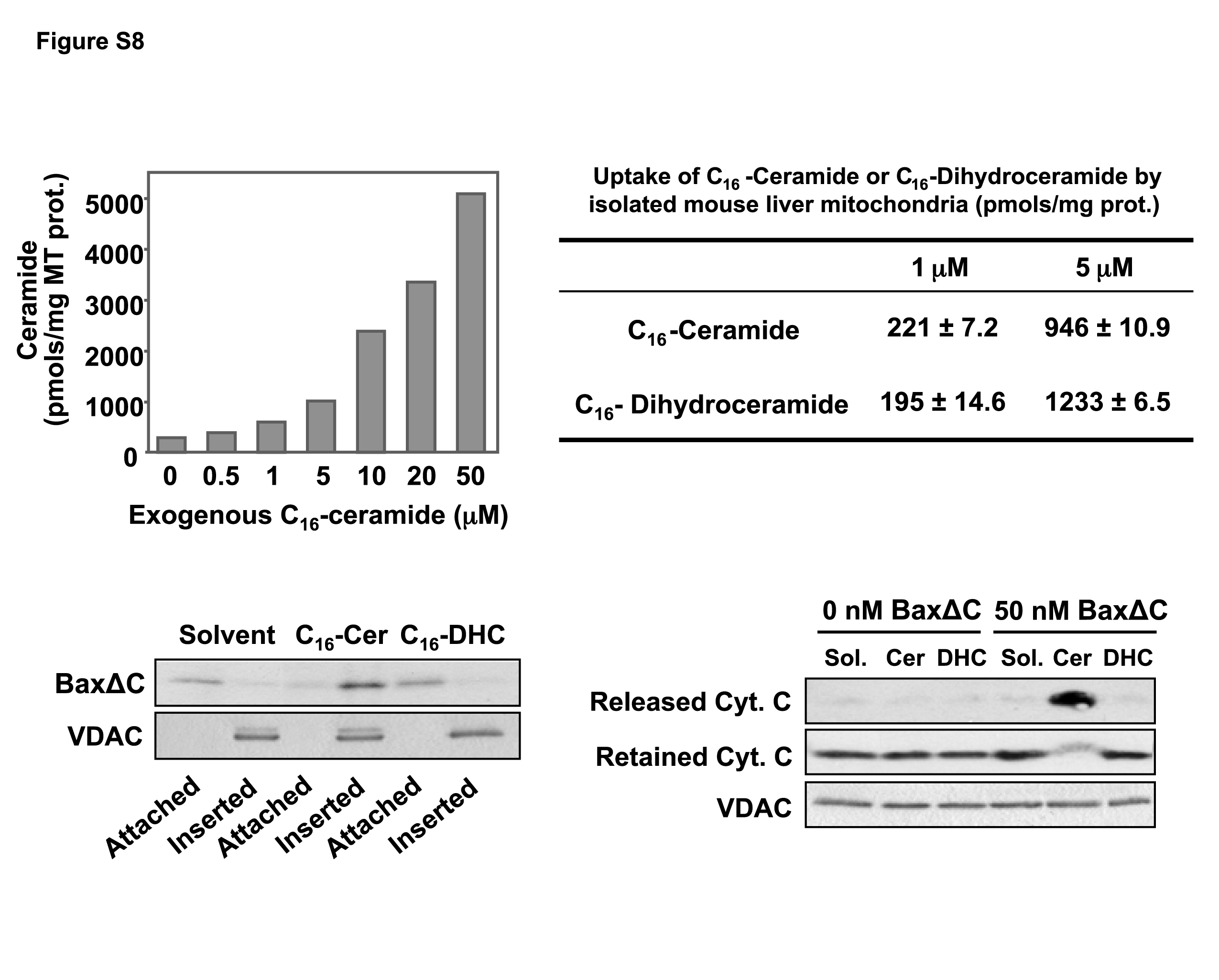

Supplement: Figure S8 — C16-ceramide and C16-dihydroceramide uptake by isolated mouse liver mitochondria and their effect on BaxΔC insertion and cytochrome c release. Upper panel: C16-ceramide or C16-dihydroceramide was added to 5 mg mitochondria in 1×KCl buffer (1 mg mitochondrial prot./ml) and incubated for 5 min at 37°C. Mitochondrial pellets were collected by centrifugation for 10 min at 10,000×g at 4°C, and lipids were extracted and ceramide and dihydroceramide were measured using the diacylglycerol kinase assay as in Materials and Methods. Lower panel: 1 µM C16-ceramide or C16-dihydroceramide was incubated with or without 50 nM BaxΔC with mitochondria and BaxΔC insertion (left) and cytochrome c release (right) were analyzed as in Figure 4A and B. (TIF) [file pone.0019783.s008.tif]

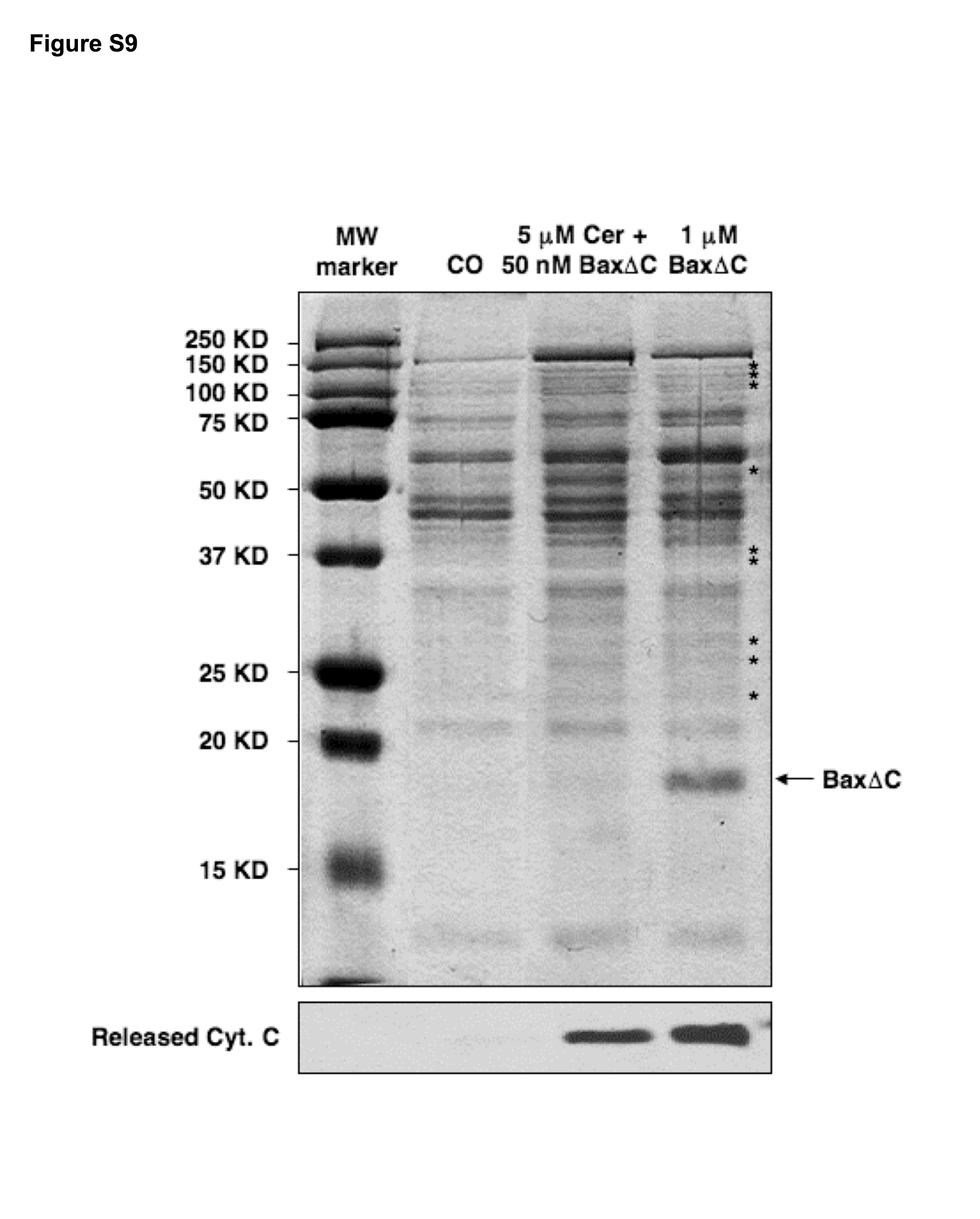

Supplement: Figure S9 — Profile of the proteins released from isolated mouse liver mitochondria by exogenous C16-ceramide and recombinant BaxΔC. Mitochondria isolated from mouse liver were incubated with KCl buffer (1 mg mitochondrial protein/ml) without BSA for 5 min at 37°C with C16-ceramide and BaxΔC as indicated. After incubation, reaction samples were centrifuged at 14,000×g for 5 min at 4°C and the supernatants containing proteins released from mitochondria were collected and analyzed by Coomassie blue staining (15 µg/lane; upper panel) or by immunoblotting (15 µg/lane) with anti-cytochrome c (lower panel). The control (CO) profile in the absence of either BaxΔC or ceramide is very similar to that observed in isolated rat liver mitochondria [77]. 50 nM BaxΔC plus 5 µM C16-ceramide and 1 µM BaxΔC display a nearly-identical set of additionally released proteins, marked by asterisks (*), indicative of the same release mechanism. Further, no inner mitochondrial space proteins over 116 kD in size were released by 50 nM BaxΔC plus 5 µM C16-ceramide or 1 µM BaxΔC, as confirmed by mass spectrometry, consistent with published literature that defines the maximal size of proteins released through the Bax pore as 123 kD [78]. (TIF) [file pone.0019783.s009.tif]

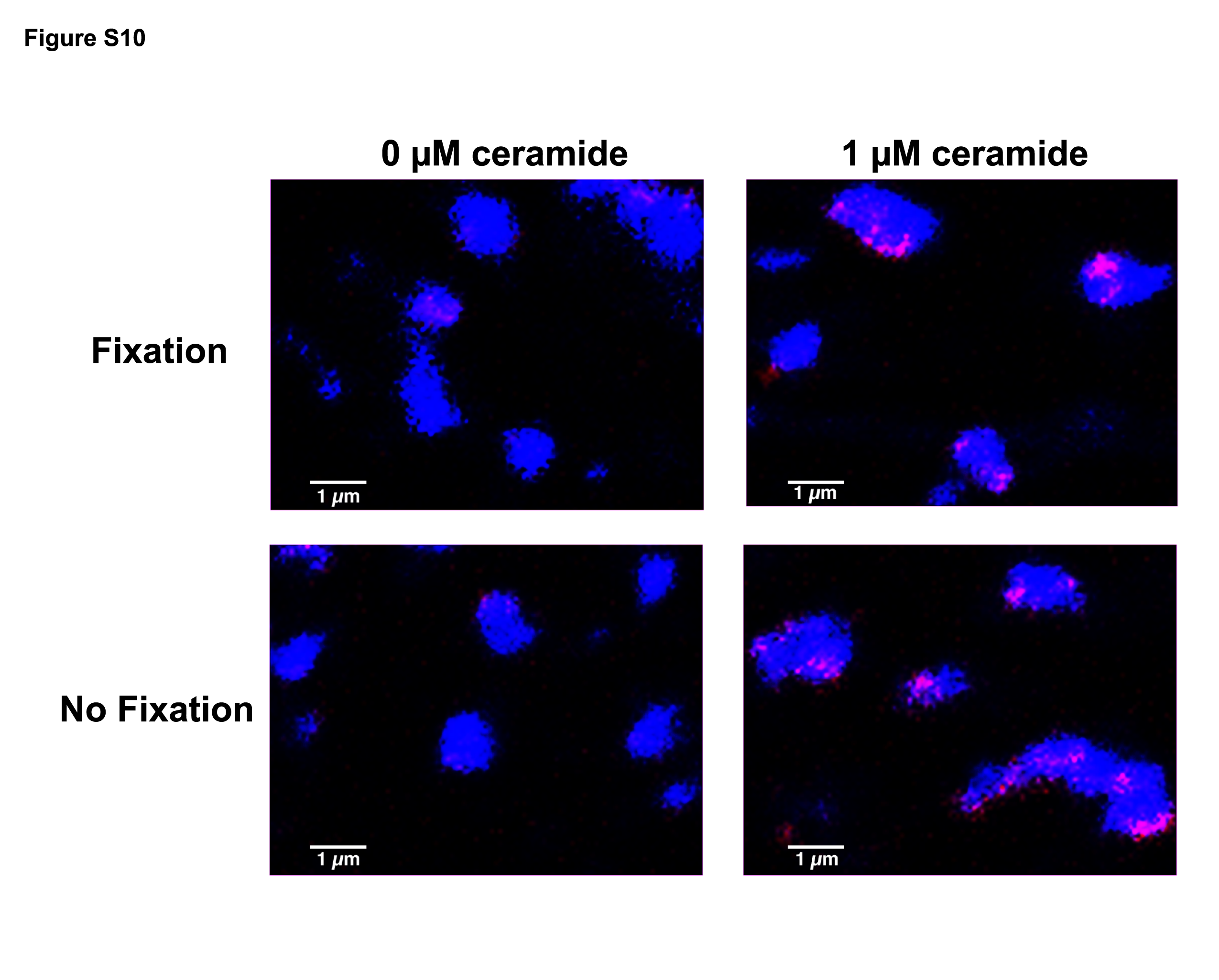

Supplement: Figure S10 — The effect of fixation on the staining of MCRM using anti-ceramide IgM. It has been reported by Butor et al. [58] that in some instances secondary antibodies induce clustering of lipids on cell surfaces, rectified if the fixation step is carried out after the first antibody-labeling step. To exclude this possibility, we compared the staining profile of MCRMs using anti-ceramide IgM with or without fixation using 2% formaldehyde. Mouse hepatic mitochondria were isolated and treated with 0 mM or 5 mM ceramide for 5 min at 37°C to induce MCRM formation as described in Materials and Methods. After incubation with blocking solution (3% FBS/3% goat serum) containing 200 nM Mitotracker for 60 min on ice, one set of samples was stained directly with anti-ceramide IgM, followed by fixation with 2% formaldehyde and the other was fixed with 2% formaldehyde, then stained with anti-ceramide. Mitochondria were subsequently incubated with secondary anti-IgM antibody and mounted on slides. Images, acquired with a Leica TCS AOBS SP2 confocal microscope equipped with a 100×1.4NA OIL DIC D objective combined with 2× scan zoom, were analyzed with MetaMorph 7.5 software. (TIF) [file pone.0019783.s010.tif]

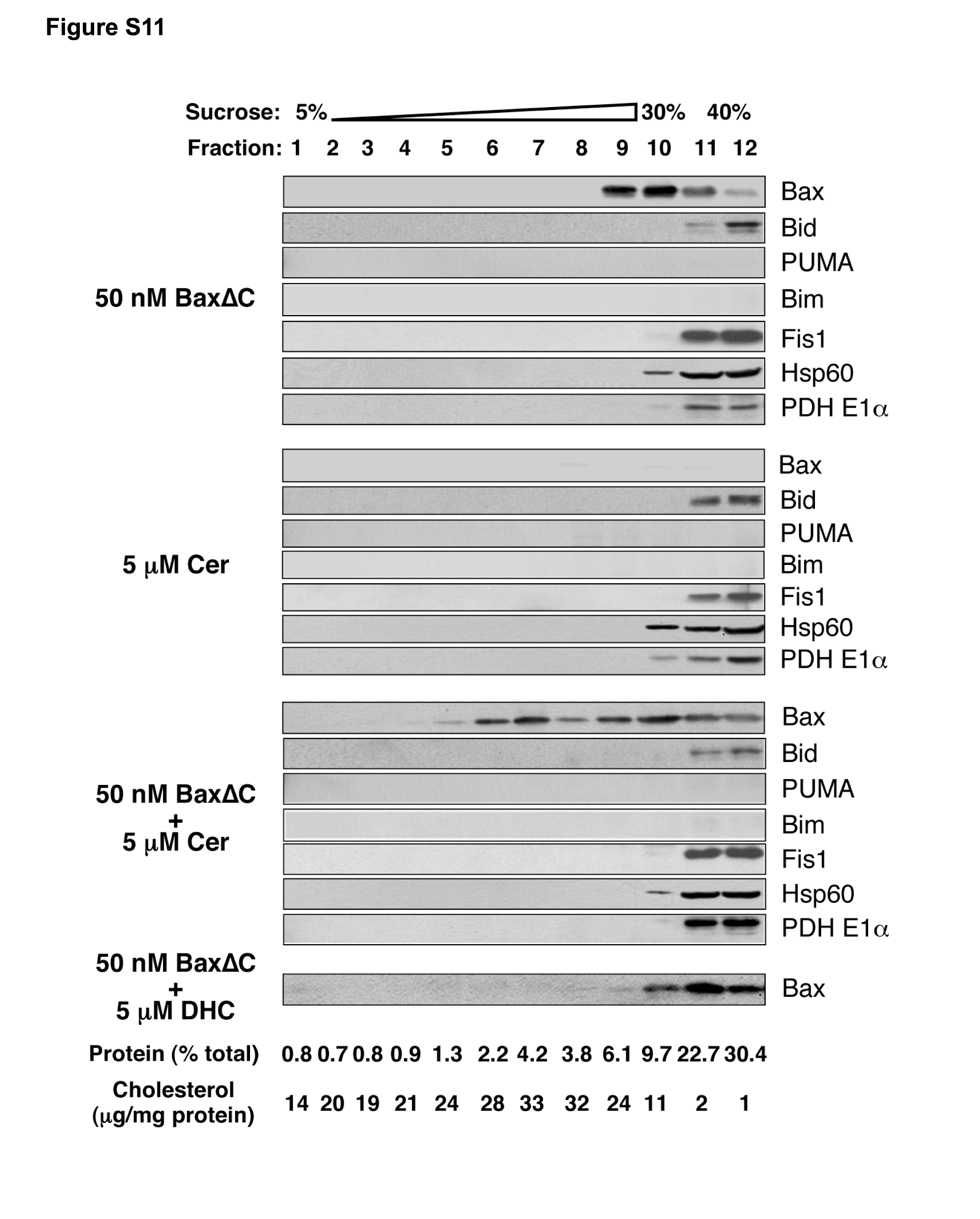

Supplement: Figure S11 — Distribution of mouse liver mitochondrial proteins in 5–30% continuous sucrose density gradient. After incubation for 5 min at 37°C with 50 nM BaxΔC with or without 5 µM C16-ceramide, mitochondria were pelleted by centrifugation at 10,000×g for 10 min at 4°C and resuspended in cold MBS buffer containing 0.05% Triton X-100. After 30 min incubation on ice, mitochondria were homogenized with 20 strokes of a loose-fitting dounce homogenizer. The mitochondrial homogenate was adjusted to 40% final sucrose concentration and subjected to 5–30% continuous sucrose density gradient centrifugation as described in Materials and Methods. 400 µl of each 1 ml fraction was used for immunoblot analysis using the indicated antibodies as in Figure 4E. A set of pro-apoptotic proteins including (Bax, Bak, Bim, Bid, PUMA, VDAC), anti-apoptotic proteins (Bcl-xL, Hsp60), and non-apoptotic proteins (COXII, Metaxin, PDH E1α) that exist in distinct compartments were examined. While Bax, Bak, Bim, Bid, PUMA, VDAC, Metaxin and Bcl-xL are outer membrane proteins, COXII is an inner membrane protein and Hsp60 and PDH E1α are matrix proteins. (TIF) [file pone.0019783.s011.tif]

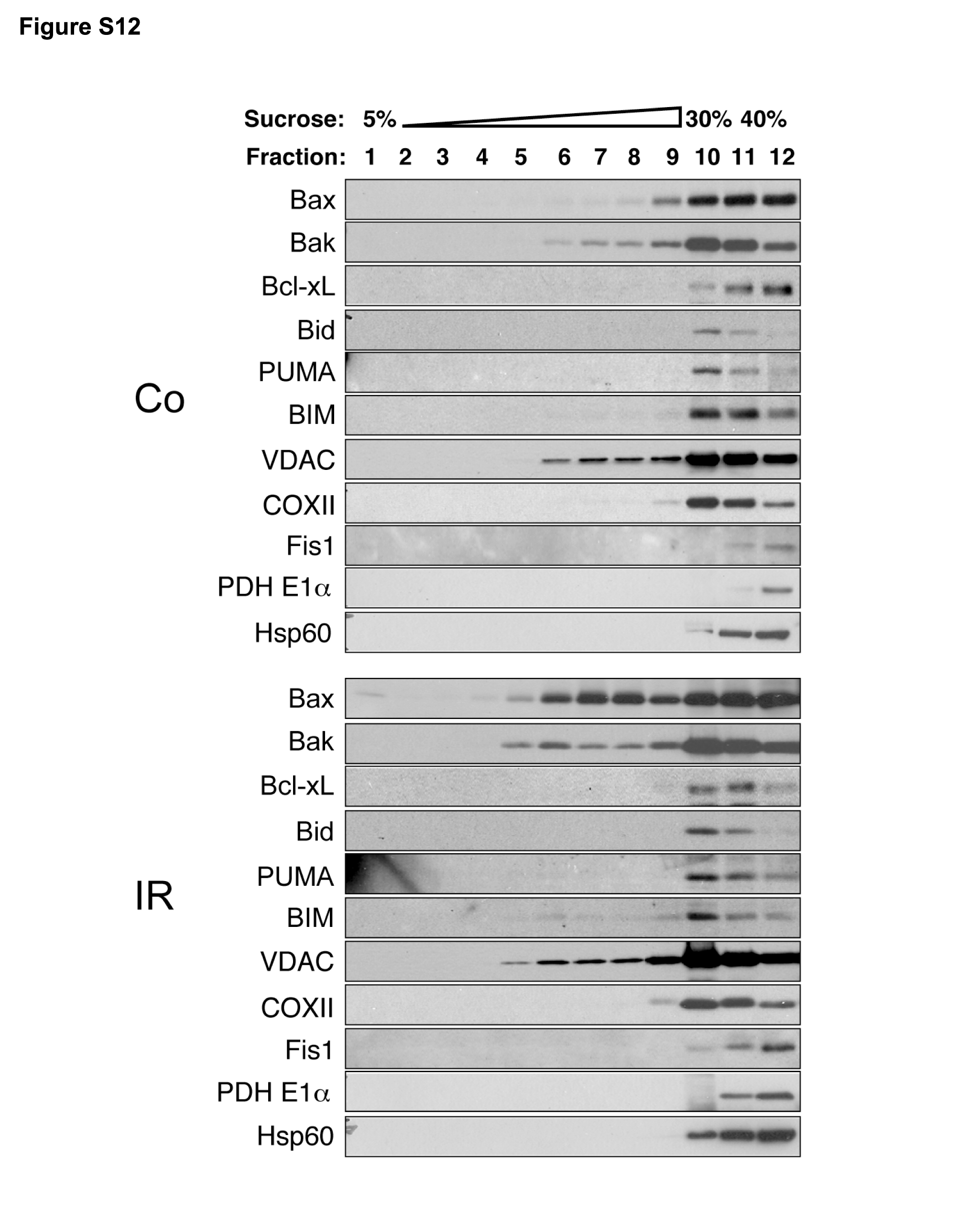

Supplement: Figure S12 — Ionizing radiation (10 Gy) induces specific translocation of Bax into HeLa cell MCRMs. Mitochondria were isolated from HeLa cells 34 h after irradiation and incubated with 0.15% Triton X-100 in MBS buffer for 30 min on ice. The mitochondrial suspension was homogenized with 20 strokes of a loose-fitting dounce homogenizer, adjusted to 40% final sucrose concentration and subjected to 5–30% continuous sucrose density gradient centrifugation as described in Experimental Procedures. 400 µl aliquots from each fraction were analyzed by Western blotting using the indicated antibodies after 20% TCA precipitation. (TIF) [file pone.0019783.s012.tif]
