# Supplementary material for: Mitochondrial Ceramide-Rich Macrodomains Functionalize Bax upon Irradiation
Source: PLoS One. 2011 Jun 13;6(6):e19783. doi: 10.1371/journal.pone.0019783 (PMC3113798; doi:10.1371/journal.pone.0019783)
Supplement: Text S1 — (DOC) [file pone.0019783.s013.doc]

**Supporting Information**

# Mitochondrial ceramide-rich macrodomains functionalize Bax upon irradiation

Hyunmi Lee^1^, Jimmy A. Rotolo^1^, Judith Mesicek^1^, Tuula Penate-Medina^1^, Andreas Rimner^1^, Wen-Chieh Liao^2^, Xianglei Yin^1^, Govind Ragupathi^3^, Desiree Ehleiter^2^, Erich Gulbins^4^,

Dayong Zhai^5^, John C. Reed^5^, Adriana Haimovitz-Friedman^2^, Zvi Fuks^2^, and Richard Kolesnick^1*^

**Experimental Procedures**

**Cell culture, irradiation and FB_1_-treatment -** HeLa cells (ATCC) were cultured in low glucose Dulbecco’s Modified Eagle’s Medium (DMEM from Gibco BRL) containing 10% fetal bovine serum (FBS), penicillin (50 units/ml), streptomycin (50 µg/ml) and 2 mM glutamine. 1 h prior to irradiation, HeLa cells were changed to medium containing 0.2% FBS. BAEC were cultured until confluent in low glucose DMEM supplemented with 10% normal calf serum, 1 ng/ml basic FGF (Scios Inc.), penicillin (50 units/ml), streptomycin (50 µg/ml) and 2 mM glutamine. After confluence, BAEC were maintained in DMEM with 5% heat inactivated normal calf serum. At 24 h before irradiation, medium was changed to DMEM with 0.2% human albumin (HA) containing 50 µg/ml pentosan polysulfate (PPS; Sigma). FB_1_ (Sigma or Biomol) was solubilized in phosphate-buffered saline (PBS; 10 mM KH_2_PO_4_, 150 mM NaCl, 3 mM NaH_2_PO_4_·7H_2_O) at a concentration of 5 mM and added to cells at final concentration of 10-50 µM FB_1_. Note commercially-available FB_1_ is a biologic isolate from *Fusarium verticillioides* and *Fusarium proliferatum* that displays batch-to-batch variation. Hence, each batch must be tested empirically for effectiveness. Irradiation was carried out at 22°C using a Cs-137 irradiator (Shepherd Mark-I, model 68, SN 643) at a dose rate of 240 cGy/min.

**Antibodies and Immunoblotting -** Anti-Bax (N20, Santa Cruz; 0.4 µg/ml or YTH-6A7, Trevigen; 0.2 µg/ml), anti-Bak (NT, Upstate Biotechnology; 2 µg/ml), anti-Bcl-xL (Transduction Laboratories; 0.5 µg/ml), anti-BID (AF860 R&D; 1 µg/ml), anti-BIM (Calbiochem; 1:1000), anti-PUMA (N-terminal, Sigma; 1 µg/ml), anti-GST (Z-5, Santa Cruz; 0.2 µg/ml), anti-Fis1 (Imgenex; 1:1000), anti-COX II (12C4, Molecular Probes; 0.2 µg/ml), anti-COX IV (20E8, Molecular Probes; 0.2 µg/ml), anti-PDH-E1α (9H9, Molecular Probes; 0.2 µg/ml), anti-Hsp60 (LK-1, Calbiochem; 0.05 µg/ml), anti-KDEL (10C3, Abcam; 1:50), anti-VDAC [31HL(Ab-3), Calbiochem; 2 µg/ml], anti-Climp63 (G1/296, Alexis; 1 µg/ml), anti-Calnexin (H-70, Santa Cruz; 0.2 µg/ml) or anti-metaxin (Transduction laboratories; 0.5 µg/ml) was used as indicated. Proteins were separated on a 12-15% SDS-PAGE gel and transferred to 0.2 µm PVDF membrane (Biorad). Then the blot was incubated with 5% Blotto (Biorad) in TBST (20 mM Tris–HCl, pH 7.5, 500 mM NaCl, 0.1 % Tween 20) for 1 h at room temperature. After brief washing with TBST, the blot was incubated with primary antibodies in TBST containing 2.5% BSA for 16 h at 4°C. This was followed by extensive washing with TBST and incubation with the appropriate secondary antibodies conjugated to HRP (Amersham) in TBST containing 5% Blotto for 1 h at room temperature. After extensive washing with TBST, immunolabeled proteins were revealed using ECL kit (Amersham).

### Apoptosis assay - Apoptotic HeLa cells were stained with *bis-*benzimide Trihydrochloride (Hoechst #33258; Sigma) and quantified by fluorescence microscopy, as described (Bose et al., 1995).

**Caspase assay -** Caspase 3 activity was measured fluorospectrometrically using Z-DEVD-AFC as substrate as described (Kluck et al., 1997b).

**Cytochrome *c* release in intact cells -** Cytosolic cytochrome *c* content was measured according to Newmeyer (Kluck et al., 1997a) by either immunoblotting using mouse monoclonal anti-cytochrome *c* antibody (7H8.2C12, PharMingen; dilution 1:500) or by ELISA (Human Cytochrome *c* Quantikine ELISA Kit, R&D).

**Isolation of subcellular fractions from HeLa cells and BAEC -** Mitochondria and endoplasmic reticulum from HeLa cells and BAEC were isolated by differential centrifugation via a discontinuous sucrose gradient as described (Tafani et al., 2001), with modification. All procedures were conducted at 4°C. For these studies, cell monolayers were washed twice and scraped into ice-cold PBS. Cells were collected by centrifugation at 500x*g* for 10 min, then washed and resuspended in SHE buffer [250 mM sucrose, 10 mM HEPES-KOH pH 7.4, 1 mM EGTA, protease inhibitor cocktail (Roche)], and homogenized twice by 20 strokes in a loose-fitting Dounce homogenizer. Cell debris and nuclei were pelleted by centrifugation at 800x*g* for 5 min. The postnuclear supernatant was collected and centrifuged at 10,000x*g* for 10 min and the pellet containing heavy membrane fractions (P_10_) was resuspended in 20 ml SHE buffer and centrifuged through an underlayed cushion of 5 ml HE medium containing 750 mM sucrose at 10,000x*g* for 30 min. The resulting mitochondrial-enriched pellet was resuspended in SHE buffer and further purified by a second discontinuous sucrose gradient centrifugation as above. MAM-free mitochondria were isolated by Percoll gradient according to the method of Vance (1990) with slight modification. For these studies, heavy membrane fractions (P_10_) were layered on top of 20 ml of medium consisting of 2.2 ml of 2.5 M sucrose, 6.55 ml of Percoll and 12.25 ml of 10 mM HEPES, pH 7.4, 1 mM EGTA, and centrifuged at 100,000x*g* for 1h in a Beckman Ti-55.2 rotor. Two bands were recovered from the gradient; a lower band corresponding to mitochondria (R_f_: 0.48) and an upper band containing MAM (R_f_: 0.23) (Vance, 1990). Mitochondria were collected using a Pasteur pipette, diluted 10-fold with SHE buffer and washed twice by centrifugation at 10,000xg for 10 min to remove the Percoll. The MAM fraction was diluted with SHE buffer and pelleted by centrifugation at 100,000x*g* for 1 h in a Beckman Ti-55.2 rotor. To isolate the ER-enriched membrane fraction, the supernatant of the P_10_ fraction was centrifuged at 100,000x*g* for 1 h. Fractionation was monitored by immunoblotting using mouse monoclonal anti-COX II (mitochondrial marker) and rabbit polyclonal anti-Climp63 or anti-Calnexin (ER Markers).

**Bax insertion status by alkali extraction of the mitochondria -** For measurement of Bax insertion, mitochondria, subjected to alkali extraction by incubation in freshly prepared 0.1 M Na_2_CO_3_ (pH 11.5) for 60 min at 4°C, were pelleted by centrifugation at 100,000x*g* for 30 min at 4°C (Antonsson et al., 2001; Mikhailov et al., 2003). Supernatant (alkaline-sensitive fraction) and mitochondrial pellet (alkaline-resistant fraction) were separated, mitochondrial membranes were lysed in RIPA buffer, and fractions were analyzed by immunoblotting using anti-Bax antibody.

**Bax oligomerization status by gel filtration -** Gel filtrations were performed at 4°C on a 1.5x55 cm Sephacryl 200 column equilibrated in 25 mM HEPES-KOH pH 7.4, 137 mM NaCl, 2 mM EDTA, 10% Glycerol, 1% or 2% (w/v) CHAPS and calibrated with thyroglobulin (669 KD), ferritin (440 KD), aldolase (158 KD), bovine serum albumin (67 KD), ovalbumin (43 KD), chymotrypsinogen A (25 KD), and ribonuclease A (13.7 KD) (Amersham Biosciences). A 1.5 ml sample was loaded onto the column and fractions of 1 ml were collected at a flow rate of 0.5 ml/min. Aliquots of 400 µl from the fractions were analyzed by Western blotting after 20% TCA precipitation. Samples were separated on 12% or 15% SDS-PAGE under reducing conditions and transferred to 0.2 µm PVDF membranes (Biorad). Proteins were detected with the specific antibodies as indicated in the figures, and the blots were developed with the ECL system from Amersham Pharmacia Biotech.

**Ceramide synthase activity assay -** For these studies, 75x10^6^ cells were pelleted, washed once with cold PBS, and resuspended in 300 µl of homogenization buffer [25 mM HEPES pH 7.4, 5 mM EGTA, 50 mm NaF, protease inhibitor cocktail (Roche)]. Cells were disrupted on ice by sonication, and lysates centrifuged at 800x*g* for 5 min. The postnuclear supernate was centrifuged at 100,000x*g* for 1 h and the microsomal pellet (P_100_) was resuspended into 1 ml of homogenization buffer. To measure ceramide synthase activity in sub-cellular fractions, mitochondrial-enriched and endoplasmic reticulum-enriched fractions were prepared as described above. Membranes were prepared fresh daily. The assay for ceramide synthase (EC 2.3.1.24) activity was based on the procedure of Harel and Futerman (Harel and Futerman, 1993). 75 µg protein were incubated in a 1 ml reaction mixture containing 2 mM MgCl_2_, 20 mM HEPES pH 7.4, 20 µM defatted BSA (Sigma), and varying concentrations (0.2-20 µM) of dihydrosphingosine (sphinganine; Biomol), 70 µM unlabeled palmitoyl-coenzyme A (Sigma), and 3.6 µM (0.2 µCi) [1-^14^C]palmitoyl-coenzyme A (55 mCi/mmol; American Radiolabeled Chemicals). Dihydrosphingosine was dried under N_2_ from a stock solution in 100% ethanol and dissolved by sonication in reaction mixture prior to addition of protein. The reaction was started by addition of palmitoyl-coenzyme A at 37°C, and after 1 h stopped by extraction of lipids using 2 ml of chloroform:methanol (1:2, v/v). 500 µl of lower phase was dried under N_2_ and dihydroceramide resolved by thin layer chromatography using a solvent system of chloroform:methanol:3.5 N ammonium hydroxide (85:15:1). Radiolabeled dihydroceramide was identified by iodine vapor staining based on comigration with ceramide standards (Type III, Sigma), and quantified by liquid scintillation counting. The velocity of the reaction was linear for at least 2 h, and the amount of palmitoyl-coenzyme A consumed did not exceed 5% of total. Ceramide synthase activity was unaffected by CHAPS (0.5-4%) or Triton X-100 (0.05-1%), except at the highest concentrations where both detergents attenuated activity 20-30%.

**Lipid analysis -** Ceramide mass was quantified by the diacylglycerol kinase assay as described (Bose et al., 1995). Cholesterol was quantified using Amplex Red Cholesterol Assay Kit according to manufacturer’s instruction (Invitrogen). Neutral sphingolipids and gangliosides were analyzed by HPTLC or immune-HPTLC as described (Hamilton et al., 1993) with modification. Sphingomyelin was analyzed according to Mallikarjuneswara et al. (Mallikarjuneswara, 1975). For these studies, 15.3 μg MCRM was extracted with CHCl_3_:MeOH:HCl (100:100:1 v/v/v), and the extract was washed once with an equal volume of pre-equilibrated upper phase, followed by resolution on Silica gel 60 Å HPTLC plates (Whatman) using CHCl_3_:MeOH:H_2_0 (65:25:4) as solvent. Sphingomyelin was visualized with 7% phosphomolybdic acid in ethanol (Sigma) followed by charring, and quantified by comparison to a concomitantly-run standard curve of known quantities of sphingomyelin.

**Cellular ceramide localization by confocal microscopy -** HeLa cells, grown on coverslips in 6-well plates, were washed 3 times with cold PBS, and fixed with 4% Formalin in PBS for 15 min. Cells were then permeabilized by incubation with-20°C acetone/methanol (1:1) for 10 min after 3 times of washing with PBS. After blocking with 3% goat serum and 3% FBS diluted in PBS for 16h at 4°C, cells were incubated with anti-CoxI-Alexa 488 conjugated (Invitrogen; 1:75) diluted in cold wash buffer (1% FBS, 0.025% Tween20 in PBS) at 22°C for 45 min. Cells were then washed 3 times for 5 min with cold wash buffer followed by incubation with monoclonal anti–ceramide (Alexis; 1:30) at 22°C for 45 min. After washing 3 times with cold wash buffer, cells were incubated with Cy3-conjugated goat anti-mouse IgM (Jackson ImmunoResearch; 1:100) at 22°C. After 45 min, cells were washed 3 times with cold wash buffer, and mounted on slides using the ProLong Antifade Kit (Invitrogen). When indicated, Mitotracker Red CMXRos (50 nM) (Invitrogen) was added to the culture medium at 37°C for 30 min to stain mitochondria before fixation. Images were acquired with a Leica TCS SP2 AOBS confocal microscope equipped with a 63x 1.4 NA OIL DIC D Objective combined with 4x scan zoom. Co-localization analysis was performed with MetaMorph 7.5 software. For ER staining, anti-KDEL (Abcam; 1:50) and anti–ceramide (Alexis; 1:20) were employed in combination with Texas red-conjugated goat anti-mouse IgG (Jackson ImmunoResearch; 1:100) and Cy2-conjugated goat anti-mouse IgM (Jackson ImmunoResearch; 1:100), respectively.

**Preparation of recombinant full length Bax and BaxΔC -** Purified recombinant full length Bax was kindly provided by Dr. Richard Youle [see (Suzuki et al., 2000)]. Mouse BaxΔC with or without a GST tag was prepared as described (Pastorino et al., 1999). Briefly, 50 ml of an overnight culture of *E. coli* *BL21* expressing pGEX-4T-2 BaxΔC was inoculated into 1000 ml of LB-medium containing 50 µg/ml of ampicillin and grown at 37°C to an A_600_ 0.6-0.8. After addition of 0.1 mM IPTG, cells were grown for 2 h at 25°C and collected by centrifugation. Cell pellets were resuspended in 30 ml lysis buffer (50 mM Tris pH 8.0, 150 mM NaCl, 1% Tween 20, 0.1% 2-mercaptoethanol, 5 mM EDTA, 1mM PMSF, protease inhibitor cocktail) and disrupted by sonication. The cell lysate was centrifuged at 28,000x*g* for 20 min at 4°C. GST-BaxΔC was purified using the Bulk GST Purification Kit (Amersham). GST tag was removed from the recombinant GST–BaxΔC proteins bound to glutathione-Sepharose 4B resin by thrombin (10 units, Amersham) according to manufacturer’s instructions. Released BaxΔC protein was purified on a Mono-Q Sepharose column using a linear gradient of 0.5 M NaCl, pH 8.0 and dialyzed in dilution buffer (20 mM HEPES pH 7.5, 100 mM KCl, 20 mM MgCl_2_, 1 mM EDTA) for 16 h at 4°C. Ultrafree Centrifuge Filter Devices (Millipore) were used to concentrate purified protein. Recombinant BaxΔC was stored in dilution buffer.

**Preparation of C_16_-ceramide and C_16_-dihydroceramide -** C_16_-ceramide (Biomol) or C_16_-dihydroceramide (Toronto Research Chemical Inc.) was dissolved in ethanol:dodecane (98:2, v/v) at 37°C with occasional vortexing, and further diluted to a 1% final solvent concentration with experimental reaction buffers.

**Bax insertion into and cytochrome *c* release from HeLa cell mitochondria ex vivo -** Bax or tBid (R&D Systems, Inc.) in Bax dilution buffer [20 mM HEPES pH 7.4, 10 mM KCl, 20 mM MgCl_2_, 1 mM EDTA] was incubated with 50 µg of the P_10_-mitochondria-enriched fraction in the presence or absence of C_16_-ceramide in 50 µl MSB buffer [400 mM mannitol, 50 mM Tris-HCl pH 7.2, 4 mM MgCl_2_, 10 mM KH_2_PO_4_, 50 μM rotenone, 5 mM succinate, 5 mg/ml fatty acid free bovine serum albumin (BSA; Sigma)] for 30 min or 1 h at 30°C. Reaction samples were centrifuged at 14,000x*g* for 5 min, and supernatants and mitochondrial pellets were analyzed for cytochrome *c* release. For measurement of Bax insertion, mitochondrial pellets were subjected to alkali extraction as described above and alkaline-sensitive and alkaline-resistant fractions were analyzed by immunoblotting using anti-Bax or anti-GST antibodies.

**Isolation of mitochondria from mouse liver –** Mouse liver mitochondria were isolated as described (Pastorino et al., 1999) with modification. Livers were surgically removed from 8 week old C57BL/6 black mice (Jackson Laboratory, Bar Harbor, ME) sacrificed by CO_2_ asphyxiation under protocol 09-08-016 in compliance with the guidelines of the Institutional Animal Care and Use Committee (IACUC) of MSKCC. Briefly, isolated liver tissue was washed twice and minced in ice-cold Buffer A (0.25 M sucrose, 10 mM HEPES pH 7.4, 0.5 mM EGTA). Minced tissue was then re-washed in Buffer A and homogenized (0.3 g of liver/ml of buffer) on ice for 3 min using a glass homogenizer with a loose-fitting pestle. Homogenates were centrifuged at 600x*g* for 15 min, and the resulting pellets containing nuclei and undisrupted cells were discarded. Supernatants were centrifuged at 9750x*g* for 15 min, and the resulting pellet was resuspended in Buffer A and centrifuged at 9750x*g* for 10 min. The pellet was washed 3 more times in Buffer A and once in Buffer B (0.25 M sucrose, 10 mM HEPES pH 7.4). The final mitochondrial pellet was resuspended in Buffer B at 50 mg protein/ml. All steps were carried out at 0-4°C. Mice were housed at the animal core facility of Memorial Sloan-Kettering Cancer Center. This facility is approved by the American Association for Accreditation of Laboratory Animal Care and is maintained in accordance with the regulations and standards of the United States Department of Agriculture and the Department of Health and Human Services, National Institutes of Health. The Institutional Animal Care and Use Committee at Memorial Sloan-Kettering Cancer Center approved these mice experiments under Protocol # 09-08-016.

**Uptake of C_16_-ceramide or C_16_-dihydroceramide by isolated mouse liver mitochondria -** Isolated mitochondria (1 μg/μl) were incubated with C_16_-ceramide or C_16_-dihydroceramide (0-5 μM; 1% final solvent concentration) in a KCl-based medium [20 mM HEPES, pH 7.4, 150 mM KCl, 25 mM NaHCO_3_, 1 mM MgCl_2_, 3 mM NaH_2_PO_4_, 250 mM sucrose, 1 mM glutamate/malate, 5 mg/ml fatty acid free BSA] for 5 min at 37°C. Thereafter, samples were centrifuged at 10,000xg for 10 min at 4°C, the mitochondrial pellet was resuspended in 1xPBS buffer, and ceramide levels in mitochondria (600 μg) were quantified by the diacylglycerol kinase assay.

**Formation of MCRMs within and cytochrome *c* release from mouse liver mitochondria ex vivo -** Recombinant full length Bax or BaxΔC in Bax dilution buffer was incubated with or without C_16_-ceramide with isolated mitochondria (1 mg/ml) in KCl-based medium for 5 or 15 min at 37°C. Reaction samples were centrifuged at 14,000x*g* for 5 min at 4°C and the supernatant containing proteins released from isolated mitochondria and pellet containing retained mitochondrial proteins were analyzed for cytochrome *c*. For MCRM isolation, the mitochondrial pellet was subjected to 5-30% continuous sucrose density gradient after incubation with 0.05% Triton X-100 as below.

**Isolation of MCRM by sucrose density gradient -** Isolation of HeLa and mouse liver MCRM was based on concepts derived from isolation of detergent-insoluble raft-like plasma membrane macrodomains (Bock et al., 2003). Identification of HeLa and mouse liver MCRM initially involved extensive detergent dose titration. Mitochondria (3.3 mg/ml), isolated in SHE buffer, were incubated on ice for 30 min with Triton X-100 (0.01-1%) or CHAPS (0.1-2%), and solubilized mitochondrial proteins were separated from insoluble proteins by centrifugation at 14,000xg for 5 min at 4°C. Extent of solubilization of the inner mitochondrial membrane protein COXIV was confirmed by Western blot. The lowest concentrations that induced complete COXIV solubilization, 0.05% TritonX-100 for mouse liver mitochondria and 0.15% Triton X-100 or 1% CHAPS for HeLa mitochondria, were selected for MCRM isolation. For these studies, mitochondria (3.3 μg/μl) were treated on ice with detergent for 30 min in 1xMBS buffer (25 mM MES pH 6.5, 150 mM NaCl), followed by homogenization with 20 strokes of a loose-fitting dounce homogenizer or by sonication (3x10 sec). The homogenate was adjusted with 80% sucrose in MBS buffer to 40% final sucrose concentration and transferred to a 12 ml ultracentrifuge tube. A continuous gradient consisting of 4 ml of 5% and 6ml of 30% sucrose, or a discontinuous gradient consisting of 5 ml of 5% and 5 ml of 30% sucrose was layered on top of the homogenate, and samples were ultracentrifuged at 182,000x*g* for 16 h in Beckman SW41 rotor at 4°C. As with isolation of plasma membrane platforms, this procedure yielded an opaque detergent insoluble band at fraction 5-7 of the gradient. One ml fractions were collected from atop the gradient and profiled for proteins by Western blotting after 20% TCA precipitation, and for lipids as in Experimental Procedures. For mini-discontinuous sucrose gradient, 80 µl of homogenates adjusted to 40% sucrose were transferred to an ultracentrifuge tube and overlaid successively with 80 µl of 30% sucrose and 80 µl of 5% sucrose. Samples were centrifuged at 182,000x*g* for 18 h in Beckman TLA 100 rotor at 4°C, and three 80 µl fractions were collected from the top. Pellets were resuspended in 80 µl MBS by brief sonication.

**Staining and visualization of MCRMs -** MitoTracker was used to outline mitochondrial anatomical boundaries for ease of co-localization, and hence cannot bestow an MCRM artifact. In studies examining MCRM formation upon radiation in HeLa cells, mitochondria were stained by incubating live cells with 100 nM MitoTracker Deep Red 633 for 30 min at 37°C as per manufacturer’ instructions before mitochondrial isolation. However, for *ex vivo* studies with isolated mitochondria examining the effects of ceramide and Bax on MCRM formation, the isolated mitochondria were stained either before or after fixation. When staining live mitochondria prior to fixation, mitochondria were pre-incubated with 200 nM MitoTracker Deep Red 633 in 2.5% FBS and 2.5% goat serum in PBS for 1 h on ice. MitoTracker is an organic dye with a charge that directs its binding to membranes with a potential, i.e. live mitochondria. However, its hydrophobic nature also permits binding membranes lacking a potential after fixation, if incubation is prolonged. Therefore, the fixed staining approach was used for the majority of *ex vivo* experiments for ease of experimental procedure. For staining of fixed mitochondria, mitochondria were fixed for 45 min with 2% formaldehyde, pelleted by centrifugation at 6000xg for 5 min, followed by brief washing with PBS, and incubation with 200 nM MitoTracker Deep Red 633 in 2.5% FBS and 2.5% goat serum in PBS for 16 h at 4°C. Mitochondria, stained with MitoTracker, were washed once in Wash Buffer (1% FBS, 0.025% Tween 20 in 1xPBS), and incubated with anti-ceramide (Alexis, MID15B4; 1:30) and/or anti-Bax (6A7; 1:50, Calbiochem or Δ21; 1:10, Santa Cruz) antibodies in Wash Buffer for 45 min. Mitochondria were then washed, followed by incubation with Cy3-conjugated goat anti-mouse IgM (Jackson ImmunoResearch; 1:100) and/or Cy2-conjugated goat anti-mouse IgG (Jackson ImmunoResearch; 1:100) or Alexa Fluor 488-conjugated goat anti-rabbit IgG (Molecular Probes; 1:100) secondary antibodies, respectively. After 45 min, mitochondria were washed 3 times, and then once in 0.025% Tween 20 in PBS. All staining steps were at 4°C. After staining, mitochondrial pellets were resuspended in 20 μl 0.025% Tween 20 in PBS. 5 μl of mitochondrial suspension was spread on slide using a cover slip and mounted using the ProLong Gold Antifade Reagent (Invitrogen). Images were acquired with a Leica TCS AOBS SP2 confocal microscope equipped with a 100x 1.4 NA OIL DIC D Objective combined with 2x scan zoom. Co-localization analysis was performed with MetaMorph 7.5 software.

**Statistical Analysis -** Statistical analysis was performed by Student's *t*-test or linear regression analysis with 95% confidence estimation for ED_50_.

**Supplemental References**

Antonsson, B., Montessuit, S., Sanchez, B., and Martinou, J.C. (2001). Bax is present as a high molecular weight oligomer/complex in the mitochondrial membrane of apoptotic cells. J Biol Chem *276*, 11615-11623.

Bock, J., Szabo, I., Gamper, N., Adams, C., and Gulbins, E. (2003). Ceramide inhibits the potassium channel Kv1.3 by the formation of membrane platforms. Biochem Biophys Res Commun *305*, 890-897.

Bose, R., Verheij, M., Haimovitz-Friedman, A., Scotto, K., Fuks, Z., and Kolesnick, R. (1995). Ceramide synthase mediates daunorubicin-induced apoptosis: an alternative mechanism for generating death signals. Cell *82*, 405-414.

Eskes, R., Antonsson, B., Osen-Sand, A., Montessuit, S., Richter, C., Sadoul, R., Mazzei, G., Nichols, A., and Martinou, J.C. (1998). Bax-induced cytochrome C release from mitochondria is independent of the permeability transition pore but highly dependent on Mg2+ ions. J Cell Biol *143*, 217-224.

Hamilton, W.B., Helling, F., Lloyd, K.O., and Livingston, P.O. (1993). Ganglioside expression on human malignant melanoma assessed by quantitative immune thin-layer chromatography. Int J Cancer *53*, 566-573.

Harel, R., and Futerman, A.H. (1993). Inhibition of sphingolipid synthesis affects axonal outgrowth in cultured hippocampal neurons. J Biol Chem *268*, 14476-14481.

Kluck, R.M., Bossy-Wetzel, E., Green, D.R., and Newmeyer, D.D. (1997a). The release of cytochrome c from mitochondria: a primary site for Bcl-2 regulation of apoptosis. Science *275*, 1132-1136.

Kluck, R.M., Martin, S.J., Hoffman, B.M., Zhou, J.S., Green, D.R., and Newmeyer, D.D. (1997b). Cytochrome c activation of CPP32-like proteolysis plays a critical role in a Xenopus cell-free apoptosis system. Embo J *16*, 4639-4649.

Mallikarjuneswara, V.R. (1975). Lecithin---sphingomyelin ratio in amniotic fluid, as assessed by a modified thin-layer chromatographic method in which a commercial pre-coated plate is used. Clin Chem *21*, 260-263.

Mikhailov, V., Mikhailova, M., Degenhardt, K., Venkatachalam, M.A., White, E., and Saikumar, P. (2003). Association of Bax and Bak homo-oligomers in mitochondria. Bax requirement for Bak reorganization and cytochrome c release. J Biol Chem *278*, 5367-5376.

Pastorino, J.G., Tafani, M., Rothman, R.J., Marcinkeviciute, A., Hoek, J.B., and Farber, J.L. (1999). Functional consequences of the sustained or transient activation by Bax of the mitochondrial permeability transition pore. J Biol Chem *274*, 31734-31739.

Siskind, L.J., Kolesnick, R.N., and Colombini, M. (2002). Ceramide channels increase the permeability of the mitochondrial outer membrane to small proteins. J Biol Chem *277*, 26796-26803.

Suzuki, M., Youle, R.J., and Tjandra, N. (2000). Structure of Bax: coregulation of dimer formation and intracellular localization. Cell *103*, 645-654.

Tafani, M., Minchenko, D.A., Serroni, A., and Farber, J.L. (2001). Induction of the mitochondrial permeability transition mediates the killing of HeLa cells by staurosporine. Cancer Res *61*, 2459-2466.

Van Loo, G., Demol, H., van Gurp, M., Hoorelbeke, B., Schotte, P., Beyaert, R., Zhivotovsky, B., Gevaert, K., Declercq, W., Vandekerckhove, J.*, et al.* (2002). A matrix-assisted laser desorption ionization post-source decay (MALDI-PSD) analysis of proteins released from isolated liver mitochondria treated with recombinant truncated Bid. Cell Death Differ *9*, 301-308.

Vance, J.E. (1990). Phospholipid synthesis in a membrane fraction associated with mitochondria. J Biol Chem *265*, 7248-7256.
