# Supplementary figures and images for: Correction: Mitochondrial Ceramide-Rich Macrodomains Functionalize Bax upon Irradiation
Source: PLoS One. 2015 Dec 30;10(12):e0146210. doi: 10.1371/journal.pone.0146210 (PMC4696794; doi:10.1371/journal.pone.0146210)

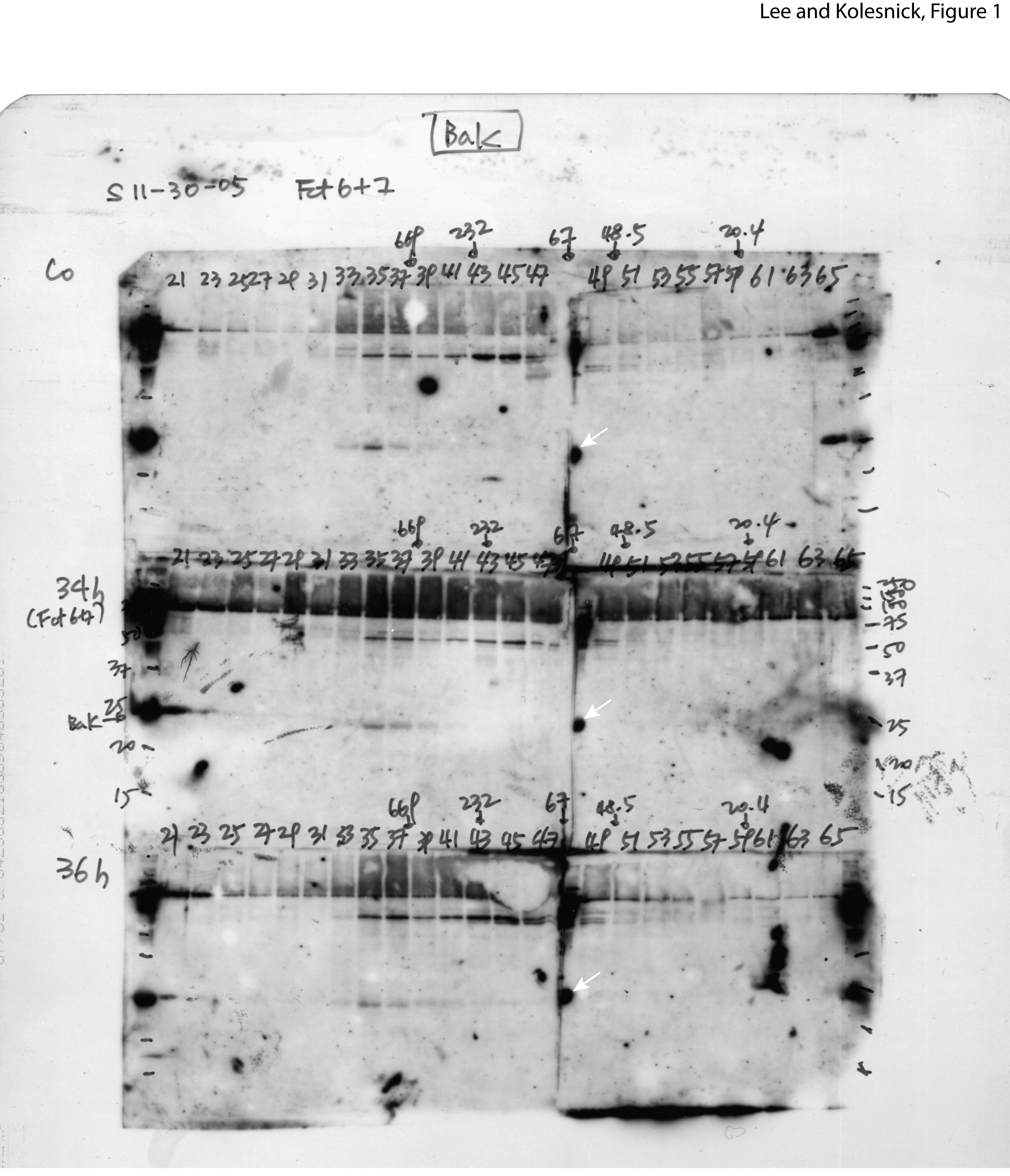

Supplement: S1 Fig — This blot, dated 11-30-05, is the original used to prepare Bak protein bands for Fig 5D(b). To analyze oligomerization status of Bax and other mitochondrial proteins such as VDAC and Bak in Mitochondrial Ceramide Rich Macrodomains (MCRMs), mitochondria were isolated from either control (Co) unirradiated HeLa cells or HeLa cells at 34 hours or 36 hours after 10 Gy. Proteins in isolated mitochondria were solubilized in 0.15% Triton X-100 or 1% CHAPS, and subjected to Dounce homogenization or sonication, respectively. Solubilized mitochondrial fractions were adjusted to 40% sucrose, placed in the bottom of a centrifugation tube, and overlaid with a 5–30% discontinuous sucrose gradient. Detergent-insoluble Light Membrane Fractions migrated to the 5%-30% sucrose interface whereas Heavy Fractions were retained in 40% sucrose after overnight centrifugation. Proteins associated with Light and Heavy Fractions were thereafter separated by molecular weight using a Sephacryl S-200 size-exclusion gel filtration chromatography column pre-calibrated with molecular weight markers in the range of 20.4 kDa to 669 KDa. One ml-sized fractions eluted from the gel filtration column were collected, and 500 μl aliquots of every other fraction between 21–65 were concentrated by TCA precipitation. Due to the large number of fractions, two 12–15% discontinuous SDS-PAGE gels were required to resolve proteins eluted in each fraction. For each elution, Gel 1 contained odd-numbered fractions 21–47 and Gel 2 contained odd-numbered fractions 49–65. To minimize variability, proteins, separated in the six gels (2 each for Control, and 34 hours and 36 hours post irradiation), were transferred to a single sheet of PVDF membrane and immunoblotted with anti-Bak antibody. Note that lanes containing molecular weight markers on the sides and the junctions of the two gels (white arrows) display immunoblot signal artifact. (TIF) [file pone.0146210.s001.tif]

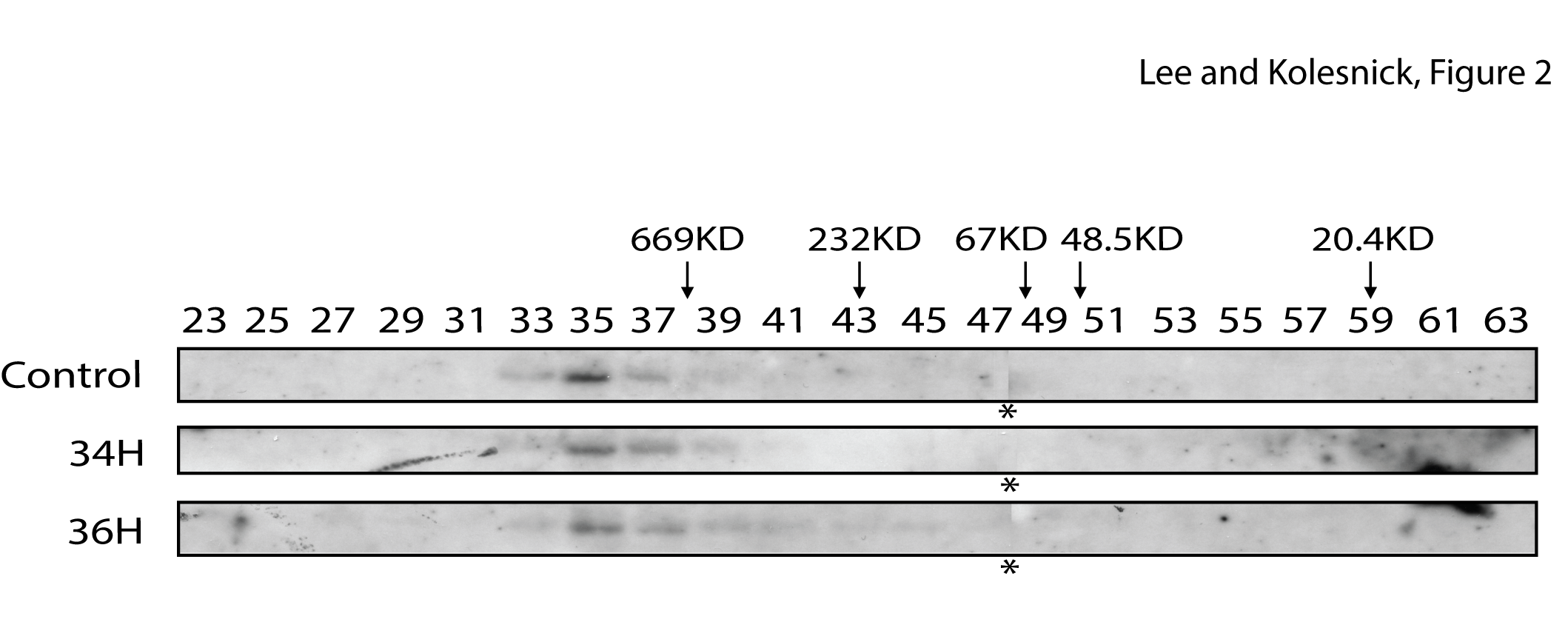

Supplement: S2 Fig — Panels displaying Bak bands isolated by gel filtration as in Figure 1 were generated from lane fractions 23–63 of the original blot and assembled in order of time of radiation. Note deletion of the molecular weight marker lanes and gel junctions for preparation of data for publication. These data show that Bak exists in HeLa mitochondrial Light Membrane Fractions before and after irradiation in a high molecular weight complex. * indicates the region where the two gels were originally apposed. (TIF) [file pone.0146210.s002.tif]

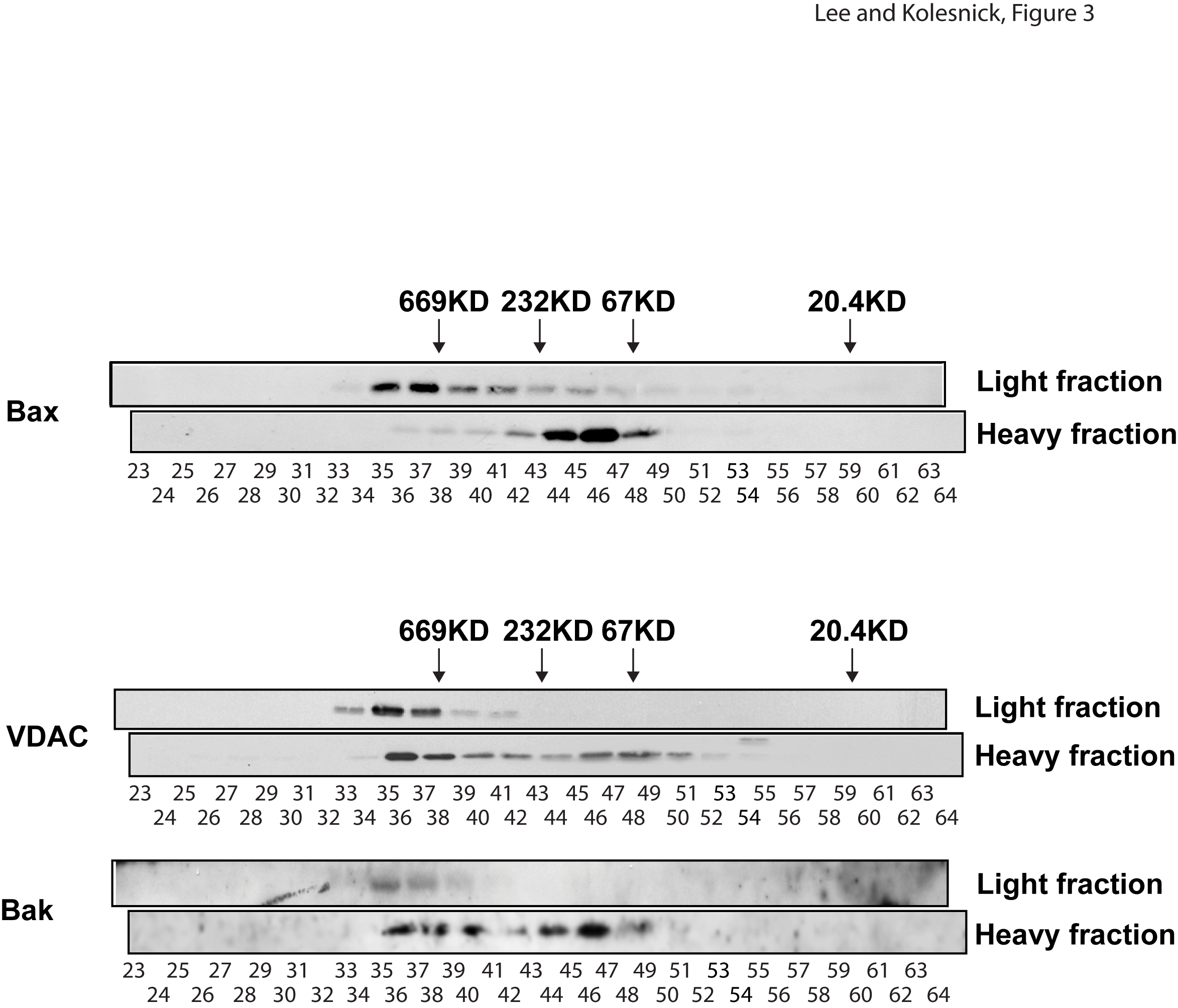

Supplement: S3 Fig — As Light Fraction Bax, VDAC and Bak from the 34 hour post radiation time point were resolved from odd-numbered fractions recovered from the Sephacryl S-200 gel filtration column while Heavy Fraction proteins were from even-numbered fractions, lanes have been renumbered and realigned. Heavy and Light Fraction lanes are now offset by one fraction. Furthermore, the Bak Light Fraction lane has been replaced with a version that more accurately reflects the Original Blot. These revisions do not alter the scientific message of the figure, which is that Bax integrates into a MCRM after radiation that contains VDAC and Bak constitutively. (TIF) [file pone.0146210.s003.tif]
